# Supplementary material for: LAGOS-NE: a multi-scaled geospatial and temporal database of lake ecological context and water quality for thousands of US lakes
Source: Gigascience. 2017 Oct 19;6(12):1–22. doi: 10.1093/gigascience/gix101 (PMC5721373; doi:10.1093/gigascience/gix101)
Supplement: Reviewer-1_Original-Submission-(Attachment).pdf [file gix101_reviewer-1_original-submission-(attachment).pdf]

GigaScience

LAGOS-NE: A multi-scaled geospatial and temporal database of lake ecological context and water quality for thousands of U.S. lakes

--Manuscript Draft--

**Manuscript Number:**	GIGA-D-17-00112
**Full Title:**	LAGOS-NE: A multi-scaled geospatial and temporal database of lake ecological context and water quality for thousands of U.S. lakes
**Article Type:**	Data Note
**Abstract:**	Background<br>Understanding the factors that affect water quality and the ecological services provided by freshwater ecosystems is an urgent global environmental issue. Predicting how water quality will respond to global changes not only requires water quality data, but also information about the ecological context of individual water bodies across broad spatial extents. Because lake water quality is usually sampled in limited geographic regions, often for limited time periods, assessing the environmental controls of water quality requires compilation of many datasets across broad regions and across time into an integrated database. LAGOS-NE accomplishes this goal for lakes in the northeastern-most 17 U.S. states.<br><br>Findings<br>LAGOS-NE contains data for 51,101 lakes and reservoirs larger than 4 ha in 17 lake-rich U.S. states. The database includes three data modules for: lake location and physical characteristics for all lakes; ecological context (i.e., the land use, geologic, climatic, and hydrologic setting of lakes) for all lakes; and in situ measurements of lake water quality for a subset of the lakes from the past three decades for approximately 2,600-12,000 lakes depending on the variable. The database contains over 200,000 measures of chlorophyll concentration and almost 900,000 measures of Secchi depth. The water quality data were compiled from 87 lake water quality datasets from federal, state, tribal, and non-profit agencies, university researchers, and citizen scientists.<br><br>Conclusions<br>This database is one of the largest and most comprehensive databases of its type because it includes both in situ measurements and ecological context data. Because ecological context can be used to study a variety of other questions about lakes, streams, and wetlands, this database can also be used as the foundation for other studies of freshwaters at broad spatial and ecological scales.
**Additional Information:**	
**Question**	**Response**
Are you submitting this manuscript to a special series or article collection?	No
**Experimental design and statistics**<br><br>Full details of the experimental design and statistical methods used should be given in the Methods section, as detailed in our Minimum Standards Reporting Checklist. Information essential to interpreting the data presented should be made available in the figure legends.<br><br>Have you included all the information requested in your manuscript?	Yes
**Resources**	Yes

Powered by Editorial Manager® and ProduXion Manager® from Aries Systems Corporation

A description of all resources used, including antibodies, cell lines, animals and software tools, with enough information to allow them to be uniquely identified, should be included in the Methods section. Authors are strongly encouraged to cite Research Resource Identifiers (RRIDs) for antibodies, model organisms and tools, where possible.<br><br>Have you included the information requested as detailed in our Minimum Standards Reporting Checklist?	
**Availability of data and materials**<br><br>All datasets and code on which the conclusions of the paper rely must be either included in your submission or deposited in publicly available repositories (where available and ethically appropriate), referencing such data using a unique identifier in the references and in the "Availability of Data and Materials" section of your manuscript.<br><br>Have you have met the above requirement as detailed in our Minimum Standards Reporting Checklist?	Yes

Manuscript

Click here to download Manuscript Soranno_etal_LAGOS-datapaper-16MAY17.docx

For submission as a ***Data Note*** *to* ***GigaScience***

LAGOS-NE: A multi-scaled geospatial and temporal database of lake ecological context and water quality for thousands of U.S. lakes

Authors:

Patricia A. Soranno[1], Linda C. Bacon[2], Michael Beauchene[3], Karen E. Bednar[4], Edward G. Bissell[1], Claire K. Boudreau[1], Marvin G. Boyer[5], Mary T. Bremigan[1], Stephen R. Carpenter[6], Jamie W. Carr[7], Kendra S. Cheruvelil[1], Samuel T. Christel[6], Matt Claucherty[8], Sarah M.Collins[6], Joseph D. Conroy[9], John A. Downing[10], Jed Dukett[11], C. Emi Fergus[12], Christopher T. Filstrup[10], Clara Funk[13], Maria J. Gonzalez[14], Linda T. Green[15], Corinna Gries[6], John D. Halfman[16], Stephen K. Hamilton[17], Paul C. Hanson[6], Emily N. Henry[18], Elizabeth M. Herron[19], Celeste Hockings[20], James R. Jackson[21], Kari Jacobson-Hedin[22], Lorraine L. Janus[23], William W. Jones[24], John R. Jones[25], Caroline M. Keson[26], Katelyn B.S. King[1], Scott A. Kishbaugh[27], Jean-Francois Lapierre[28], Barbara Lathrop[29], Jo A. Latimore[1], Yuehlin Lee[30], Noah R. Lottig[31], Jason A. Lynch[13], Leslie J. Matthews[33], William H. McDowell[34], Karen E.B. Moore[35], Brian P. Neff[36], Sarah J. Nelson[37], Samantha K. Oliver[6], Michael L. Pace[38], Donald C. Pierson[39], Autumn C. Poisson[1], Amina I. Pollard[40], David M. Post[41], Paul O. Reyes[30], Donald O. Rosenberry[42], Karen M. Roy[43], Lars G. Rudstam[44], Orlando Sarnelle[1], Nancy J. Schuldt[45], Caren E. Scott[46], Nicholas K. Skaff[1], Nicole J. Smith[1], Nick R. Spinelli[47], Joseph J. Stachelek[1], Emily H. Stanley[6], John L. Stoddard[48], Scott B. Stopyak[49], Craig A. Stow[50], Jason M. Tallant[51], Pang-Ning Tan[52], Anthony P. Thorpe[25], Michael J. Vanni[53], Tyler Wagner[54], Gretchen Watkins[4], Kathleen C. Weathers[56], Katherine E. Webster[57], Jeffrey D. White[58], Marcy K. Wilmes[59], Shuai Yuan[52]

[1]Department of Fisheries and Wildlife, Michigan State University, East Lansing, MI 48824, USA
[2]Department of Environmental Protection, State of Maine, Augusta, ME 04330, USA
[3]Department of Energy and Environmental Protection, State of Connecticut, Hartford, CT 06106, USA
[4]Water Resources Program, Lac du Flambeau Tribal Natural Resources, Lac du Flambeau, WI, USA
[5]Environmental Planning, US Army Corps of Engineers, Kansas City, MO 64106, USA
[6]Center for Limnology, University of Wisconsin Madison, Madison, WI 53706 USA
[7]Office of Watershed Management, Massachusetts Department of Conservation and Recreation, West Boylston, MA 10583, USA
[8]Watershed Protection, Tipp of the Mitt Watershed Council, Petoskey, MI 49770, USA
[9]Division of Wildlife, Inland Fisheries Research Unit, Ohio Department of Natural Resources, Hebron, OH 43025, USA
[10]Large Lakes Observatory, University of Minnesota, Duluth, MN 55812 USA
[11]Adirondack Lake Survey Corporation, Ray Brook, NY 12977 USA
[12]National Research Council, US Environmental Protection Agency, Corvallis, OR 97333, USA
[13]Office of Air and Radiation, US Environmental Protection Agency, Washington, DC 20460, USA
[14]Department of Biology, Miami University, Oxford, OH 45056, USA
[15]Natural Resource Science, University of Rhode Island, Kingston, RI 02892 USA
[16]Geoscience, Hobart & William Smith Colleges, Geneva, NY 14456 USA
[17]Kellogg Biological Station, Michigan State University, Hickory Corners, MI 49060, USA
[18]Outreach and Engagement, Oregon State University, Corvallis, OR 97331, USA
[19]Watershed Watch, University of Rhode Island, Kingston, RI 02881, USA
[20]Natural Resource Department, Lac du Flambeau Band of Lake Superior Chippewa Indians, Lac du Flambeau, WI 54538, USA
[21]Department of Natural Resources, Cornell University, Bridgeport, NY, USA

[22]Office of Water Protection, Fond du Lac Reservation, Cloquet, MN 55720 USA
[23]Bureau of Water Supply, New York City Department of Environmental Protection, Valhalla, NY 10560, USA
[24]School of Public and Environmental Affairs, Indiana University, Bloomington, IN 47408, USA
[25]School of Natural Resources, University of Missouri, Columbia, MO, USA
[26]Natural Resource Department, Little Traverse Bay Bands of Odawa Indians, Harbor Springs, MI 49740, USA
[27]Division of Water, New York State Department of Environmental Conservation, Albany, NY 12233, USA
[28]Department of Biological Science, University of Montreal, Montreal Quebec, Canada, H3C 3J7
[29]Pennsylvania Department of Environmental Protection, State of Pennsylvania, Harrisburg, PA 17101 USA
[30]Office of Watershed Management, Massachusetts Department of Conservation and Recreation, Belchertown, MA 01007, USA
[31]Trout Lake Research Station, University of Wisconsin, Boulder Junction, WI 54512, USA
[33]Lakes and Ponds Program, Vermont Department of Environmental Conservation, Montpelier, VT 05620, USA
[34]Natural Resources and the Environment, University of New Hampshire, Durham, NH 03824, USA
[35]Water Quality Science and Research, New York City Department of Environmental Protection, Kingston, NY 12401, USA
[36]Denver Federal Center, USGS, Lakewood, CO 80225, USA
[37]School of Forest Resources, University of Maine, Orono, ME, USA
[38]Department of Environmental Science, University of Virginia, Charlottesville, VA 22904, USA
[39]Department of Ecology and Genetics, Uppsala University, Uppsala, Sweden
[40]Office of Water, US EPA, Washington, DC 20460, USA
[41]Ecology and Evolutionary Biology, Yale University, Connecticut 06511, USA
[42]National Research Program, USGS, Denver, CO 80225, USA
[43]Division of Air Resources, New York State Department of Environmental Conservation, Ray Brook, NY 12977, USA
[44]Department of Natural Resources, Cornell University, Ithaca, NY 14850, USA
[45]Environmental Program, Fond du Lac Band of Lake Superior Chippewa Indians, Cloquet, MN 55720, USA
[46]Aquatic Science, NEON, Boulder, CO 80301, USA
[47]Watershed Management, Lake Wallenpaupack Watershed Management District, Hawley, PA, USA
[48]Western Ecology Division, Office of Research and Development, US EPA, Corvallis, OR 97333, USA
[49]Technology Services, Eaton County, Charlotte, MI, USA
[50]Great Lakes Environmental Research Lab, NOAA, Ann Arbor, MI 47176, USA
[51]Biological Station, University of Michigan, Pellston, MI 49769, USA
[52]Computer Science and Engineering, Michigan State University, East Lansing, MI 48824, USA
[53]Department of Zoology, Miami University, Oxford, OH 45056 USA
[54]Pennsylvania Cooperative Fish and Wildlife Research Institute, Pennsylvania State University, University Park, PA 16802, USA
[56] Cary Institute of Ecosystem Studies, Millbrook, NY, USA
[57]School of Natural Sciences, Trinity College, Dublin, Ireland
[58]Biology Department, Framingham State University, Framingham, MA 01702, USA
[59]Department of Environmental Quality, State of Michigan, Lansing, MI 48909, USA

ABSTRACT

Background

Understanding the factors that affect water quality and the ecological services provided by freshwater ecosystems is an urgent global environmental issue. Predicting how water quality will respond to global changes not only requires water quality data, but also information about the ecological context of individual water bodies across broad spatial extents. Because lake water quality is usually sampled in limited geographic regions, often for limited time periods, assessing the environmental controls of water quality requires compilation of many datasets across broad regions and across time into an integrated database. LAGOS-NE accomplishes this goal for lakes in the northeastern-most 17 U.S. states.

Findings

LAGOS-NE contains data for 51,101 lakes and reservoirs larger than 4 ha in 17 lake-rich U.S. states. The database includes three data modules for: lake location and physical characteristics for all lakes; ecological context (i.e., the land use, geologic, climatic, and hydrologic setting of lakes) for all lakes; and in situ measurements of lake water quality for a subset of the lakes from the past three decades for approximately 2,600-12,000 lakes depending on the variable. The database contains over 200,000 measures of chlorophyll concentration and almost 900,000 measures of Secchi depth. The water quality data were compiled from 87 lake water quality datasets from federal, state, tribal, and non-profit agencies, university researchers, and citizen scientists.

Conclusions

This database is one of the largest and most comprehensive databases of its type because it includes both in situ measurements and ecological context data. Because ecological context can be used to study a variety of other questions about lakes, streams, and wetlands, this database can also be used as the foundation for other studies of freshwaters at broad spatial and ecological scales.

KEYWORDS

Lake eutrophication, Nutrients, Water quality, lake trophic state, Ecological context, LAGOS-NE, Open science, Lake database

1. Data Description

A major concern for water quality in freshwaters globally is cultural eutrophication, or excess nutrient inputs from human activities that lead to increased plant and algal growth. In many parts of the world, runoff from land, or nonpoint source pollution, has replaced discharges of sewage, or point-source pollution, as the primary driver of lake and reservoir eutrophication [1]. In lakes and reservoirs, eutrophication is expected to become more widespread in the coming decades as the human population increases and climate and land use change commensurately, placing increasing pressures on freshwaters [2,3,4]; although, there is also recognition that eutrophication or its response to management actions does not progress the same in all lakes (e.g., [5,6,7]). Most research to understand lake nutrients and their effects on algae, plants, and aquatic food webs has been conducted in individual or small groups of lakes by studying the complex within-lake mechanisms that control responses to nutrients (e.g., [8,9]). Such relationships and interactions have also been found to be influenced by the ecological context of lakes (i.e., the land use, geologic, climatic, and hydrologic setting of lakes), which varies by lake and region, and is multi-scaled. In fact, it is not always clear whether local or regional ecological context matters more for predicting lake eutrophication (e.g., [10,11,12]). Therefore, determining the current extent of lake eutrophication and predicting how eutrophication will respond to future global change requires water quality data (e.g., nutrients, water clarity, and chlorophyll concentrations) and measures of lake ecological context across regions, the continent, and the globe (e.g., [13,14,15]).

In practice, measures of water quality are often collected from a relatively small number of lakes within individual regions. In the U.S., large investments have been made in water quality monitoring by federal, state, local, and tribal governments; and, many, but not all, of the datasets have been placed in government data repositories such as the USGS National Water Information System (NWIS) and the USEPA Storage and Retrieval (STORET) database. Unfortunately, these data repositories do not currently allow us to study lake water quality at broad scales. Despite the large number of water quality records in these systems, a recent analysis of their stream nutrient data found that over half of the data records lacked the most critical metadata necessary to make the data usable (e.g., chemical form, parameter name, units; [16]); and, we would expect a similar result with lake data because they are typically treated similarly to stream nutrient data. In addition, STORET and NWIS do not include any measures of lake ecological context. Therefore, to study the controls of eutrophication specifically, and water quality in general, requires development of a comprehensive database for lake water quality that is integrated with measures of lake ecological context and sufficient metadata for robust analysis.

We created such a database for thousands of inland lakes in 17 of the most lake-rich states in the upper midwest and northeastern U.S. named LAGOS-NE, the 'lake multi-scaled geospatial and temporal database' (Figure 1). We avoided the problem of lack of metadata for the water quality data by contacting the original data providers for water quality data, asking for metadata, and only including data for which sufficient metadata were available. We addressed the problem of lack of ecological-context data by creating our own database of lake ecological context. The detailed methods and approach for building this database have been published previously [17]; here we publish and describe the database for the 51,101 lakes and reservoirs ≥ 4 ha in the study area (1,800,000 km^2).

We had three related motivations for developing this database: (1) to facilitate further development of our basic understanding of lake water quality at broad scales using water quality data on thousands of lakes collected over the last several decades (see [11,17] for details); (2) to build the capacity to apply this scientific understanding to environmental management and policy of inland waters; and, (3) to foster broad-scale research by designing an open-science database that is extensible for future uses and by making the data and methods publicly accessible.

Figure 1. Map of the study extent of LAGOS-NE. Map includes 17 states in the upper midwest and northeastern U.S. outlined in white and 51,101 lakes ≥ 4 ha shown as blue polygons. Some lakes extend beyond state borders and are included in the database if it was possible to delineate their watersheds. Watershed boundaries rather than state boundaries were used for all analyses of lakes, streams and wetlands. The map is modified from [17].

LAGOS-NE is composed of three data modules that, although integrated in the same database, were derived using different data sources and data integration methods, and thus must be version-controlled separately. LAGOS-NE$_{LOCUS}$ v1.01 includes lake location and physical characteristics based on an existing national-scale database of lake and streams in the U.S. for all lakes. LAGOS-NE$_{GEO}$ v1.05 includes measures of land, water, and air (ecological context) obtained from existing national scale GIS (geographic information system) datasets and measured in multiple zones (delineated by different spatial classifications) around all lakes. This module also contains some temporal data for climate, land use/cover, and atmospheric deposition variables. LAGOS-NE$_{LIMNO}$ v1.087.1 is composed of in-situ measurements of lake water quality for a subset of the above lakes. These 87 datasets of lake water quality were obtained from a combination of sources including government, tribal agencies, university researchers, citizen scientists, and non-profit agencies. Samples were taken during any season of the year from the ~~most recent decades up until about 2012~~.

The largest challenge in building LAGOS-NE was the heterogeneity of the dataset formats, variable conventions and units, and metadata, none which were standardized. Many steps of data integration required manual input from experts in diverse fields and close collaboration among specialists in ecoinformatics, database design, freshwater ecology, and geography; all combined, the effort took six years and involved 10-20 individuals at any one time, spread across numerous institutions.

We designed the database using principles of open science so futures users could ask new research questions by using the existing database or adding new data modules to the database. To ensure users could do this, we documented the major steps of dataset integration and carefully integrated metadata directly into the database itself, we emphasized data provenance, and we used a database versioning system. In this data paper, we make the following research products available: (1) data tables with the data that make up LAGOS-NE and an R package for accessing the data and integrating the tables; (2) for each of the 87 water quality datasets, we provide the EML (ecological metadata language) metadata files that we authored after receiving the data, the data files that we processed to import into LAGOS-NE, and the R-script that we wrote to process the data; and (3) GIS coverages of the underlying freshwater geographic features (lakes, streams and wetlands) that are linked to the data tables for GIS processing by researchers.

2. Study site: Midwest and Northeast U.S. lakes

We selected an area of the U.S. known to have large numbers of lakes, well-developed lake water quality sampling programs, and that spans diverse geographic conditions and thus gradients of ecological context (Table 1). Our study area of 17 U.S. states includes 51,101 lakes ≥ 4 ha (Figure 1). These states are in the north temperate climatic zone, which experience cold winters and warm, humid summers. The study area includes part of the Interior Plains, Laurentian Uplands, Appalachian Highlands, and Atlantic Plain geological provinces, and thus encapsulate a range of geological ages, glacial histories, and topography. Land use/cover is highly variable, ranging from regions of intense agriculture in the corn belt that spans portions of Minnesota, Wisconsin, Iowa, Missouri, Indiana, and Ohio, to predominantly forested or urban regions of the northeastern U.S., including the states of Maine, New Hampshire, New Jersey, and parts of New York, and primarily forested regions of northern Minnesota, Wisconsin, and Michigan.

Although the majority of the data that we provide are for lakes ≥ 4 ha (see below for reasons for using this threshold), we do include some data on lakes ≥ 1 ha and < 4 ha if data were available. Although there may be water quality data for some lakes in this smaller size range, ecological context variables are not available for these lakes.

Table 1: Summary statistics for LAGOS-NE study area.

State	Area (km2)	Number of lakes (≥4 ha)	Mean annual temperature (°C)	Mean annual precipitation (mm)	% Agricultural land	% Urban land	% Forested land	% Wetland
Connecticut	12,878	763	9.7	1253	7.2	24.4	54.5	9.0
Illinois	145,920	2,819	11.3	1005	68.9	11.9	15.0	1.7
Indiana	93,717	1,874	11.2	1072	62.0	10.8	22.5	1.5
Iowa	145,736	903	9.1	881	78.0	7.5	6.9	1.9
Maine	84,123	2,645	5.1	1149	3.7	3.5	66.9	12.1
Massachusetts	21,013	1,698	8.9	1235	5.8	25.2	50.1	12.2
Michigan	150,489	6,511	7.2	841	26.2	10.6	35.5	19.2
Minnesota	218,543	13,984	5.3	709	44.7	5.7	19.7	19.0
Missouri	180,537	1,858	12.7	1100	50.7	7.0	36.6	2.1
New Hampshire	23,980	1,109	6.5	1209	3.8	7.9	74.5	6.4
New Jersey	19,599	1,143	11.8	1188	13.8	31.1	27.9	21.4
New York	126,070	4,461	7.6	1094	21.9	9.3	54.1	7.2
Ohio	106,917	1,279	10.6	1003	50.0	14.7	30.9	1.0
Pennsylvania	117,293	1,755	9.3	1109	22.7	12.3	59.5	1.6
Rhode Island	2,809	253	10.0	1246	4.9	29.5	44.6	13.6
Vermont	24,913	528	5.9	1176	13.3	5.5	70.0	4.7
Wisconsin	145,295	6,009	6.6	831	36.7	7.5	35.5	13.7

This table includes numbers of lakes and geophysical setting of each state and state averages for climate and land use/cover characteristics. Temperature and precipitation data are 30 year climate normals (1981-2010); land use/cover data are from the 2011 National Land Cover Database (NLCD). Note, border lakes are only counted in one state.

3. Overview of LAGOS-NE

LAGOS-NE includes some data on all lakes in a study area (above the minimum lake area threshold, which was 4 ha), which we call the 'census' population of lakes. The census population of lakes is a critical feature of LAGOS-NE because it allows us to characterize the ecological context of every lake in our study population and to identify whether the lakes for which we have water quality data are biased in any way. LAGOS-NE includes three main categories of variables: (1) variables that describe the physical characteristics and location of lakes themselves; (2) variables that describe in-situ water quality; and (3) variables that describe a lake's ecological context at multiple scales, and across multiple dimensions (such as hydrology, geology, land use, climate, etc.) based on the principles of landscape limnology [18,19,20,12]. Three factors dictated which data were included: past research and theory about the spatial and temporal controls of lake water quality, data availability and quality, and the time and resources necessary to compile, integrate, or process the original data. In other words, data that were especially time- and resource-intensive to collate, integrate, or process were given lowest priority and in some cases, were not ultimately incorporated into the database.

There were a number of constraints for each of the categories of data that had to be considered. For creating the census population of lakes (i.e., their geospatial location, perimeter, and surface area), we relied on a single source of data (the 1:24,000 National Hydrography Dataset (NHD) [21]). For the in-situ water quality data, we incorporated data only if they were in a digitally-accessible format such as a text or spreadsheet file. Finally, for the ecological-context variables, we included only data for which we could obtain a GIS or raster coverage at the national or state scale for all 17 states.

We organized these three categories of data into database 'modules' that had similar data types and sources so that we could develop procedures and set standards for each module (Figure 2). The module structure also facilitates data reuse and extension by accommodating future data modules related to any other lake or ecological-context feature.

Figure 2. LAGOS-NE data modules and version numbers. The data modules and versions that are included in LAGOS-NE and are available with this paper include: LAGOS-NE$_{GEO}$ v.1.05, LAGOS-NE$_{LOCUS}$ v.1.01 (note, that in Soranno et al. [17], this module was called LAGOS-lakes), and LAGOS-NE$_{LIMNO}$v.1.087.1. We include descriptions of the type of data that are included in each module; with the major categories of variables the same as those describing the data tables in Additional File 1. The black connectors among the modules show that the modules are connected to each other through common unique identifiers through the LAGOS-NE$_{LOCUS}$ module (either the unique lake ID or the zone ID). P is phosphorus, N is nitrogen, C is carbon, S is sulfur, atm is atmospheric, NHD is the National Hydrography Dataset, IWS is the interlake watershed, WBD is the Watershed Boundary Dataset, EDU is Ecological Drainage Unit. Figure is modified from Figure 1 in Soranno et al. [17].

The design of LAGOS-NE and the workflow for its construction has been described previously in detail [17]. In particular, the database design is based on the CUAHSI ODM as described in [17]. Here, we provide a brief overview. One important guiding principle in creating LAGOS-NE was to ensure data provenance, i.e., that we could trace the original source data through to the final LAGOS-NE database. Because each data module had different types of source data, we developed different procedures for data provenance for each module, described in Soranno et al. [17] and in this paper. The database model is based on the Consortium of Universities for the Advancement of Hydrologic Science, Inc. (CUAHSI) Community Observations Data Model (ODM) because it is a flexible data model (i.e., allows the incorporation of wide range of types of data) that allows for the incorporation of controlled vocabulary and, importantly, allows for extensive documentation through a relational database structure of linked tables containing metadata [17]. The database was created and is maintained in PostgreSQL v9.1. However, for researchers to use the database for analysis and modeling, it is necessary to export the data into tables that can be processed by statistical packages or computer code. Therefore, we exported the data into a series of tables (of similar data) that are needed to conduct research on either the census population of lakes, the lakes for which there are water quality data, or some combination. These are the data files that have been used to conduct research on LAGOS-NE to date, and that we make available in this data paper (see Additional File 1 for a list of the tables and associated data that we are making available). Further, we also make our GIS datasets available to facilitate geospatial analyses of lakes, streams, and wetlands used to create some of the major components of LAGOS-NE.

4. Description of LAGOS-NE$_{LOCUS}$ v1.01 data module

The LAGOS-NE$_{LOCUS}$ module includes data on the physical location, some features and unique identifiers for all lakes in the study area $\geq$ 1 ha, which means this data file has information on 141,378 lakes. Note, that because we detected errors in the digitization of lakes between 1 and 4 ha, we have chosen to define our census population of lakes as only those $\geq$ 4 ha, but we still make data available for lakes smaller than 4 ha when available in this and the LAGOS-NE$_{LIMNO}$ data module. However, we recommend caution in analyses, interpretation, and inference for lakes < 4 ha in this database that depend on NHD's spatial representation and detection of water bodies. The data in this module include: lake unique identifiers, perimeter, area, latitude and longitude, GNIS name, and the zone IDs that the lake is located within (e.g., state, county, the hydrologic unit at each level (HU4, HU8, and HU12). The GIS datasets that we also make available provide the lake polygon features associated with this module, as well as coverages for: lake watersheds, streams, wetlands, spatial classifications, and glaciation history.

Definition of lakes: We defined lakes previously in Soranno et al. [17] as follows. A 'lake' in LAGOS-NE is a perennial body of relatively still water. We include lakes and reservoirs that range from being completely natural to highly modified: lake basins can be entirely natural, modified natural (i.e., a water control structure on a natural lake), or a fully impounded stream or river (i.e., a reservoir). We explicitly exclude: sewage treatment ponds, aquaculture ponds, and detention ponds that are known to contain basins that are entirely artificial and were built for high-intensity human use. In addition, due to their unusual nature and size, we do not include the five Great Lakes in our database. This definition of

'lake' for LAGOS-NE has been developed only for the purpose of this database and its applications (e.g., to answer questions about lake water quality). The intent of LAGOS-NE is not to document and measure the total number of water bodies in our study area, although we are able to perform this calculation for lakes ≥ 4 ha, with an acceptable level of uncertainty (see below).

Definition of lake watersheds: We calculated lake watersheds as 'inter-lake watersheds' (IWSs) defined as the area of land draining directly into the lake as well as the area that drains into upstream-connected streams and lakes < 10 ha (Figure 3). We defined lake watersheds this way to define the drainage basin of lakes that includes connected streams and their drainage basins. However, because research has shown that large upstream lakes can trap nutrients flowing into them, these large lakes can block nutrient transport of nutrients that originate upstream of them to downstream lakes in a connected lake chain (e.g., [22]). Therefore, to calculate a drainage basin for a lake with large upstream connected lakes, we did not include the drainage basins of upstream lakes > 10 ha. See Soranno et al. [17] for full details on how lake IWSs were calculated and the section on LAGOS-NE_{GEO} for further details.

Lakes near and beyond the state borders*:* For some of our analyses, we delineated boundaries in other ways than political boundaries that were more ecologically relevant, which resulted in the inclusion of some lakes outside of the exact 17 state border. This fact allowed us to include more in situ data collected by state and citizen sampling programs which do not always follow strict state borders and may include lakes that are outside of state lines. Although most of these border lakes have hydrological (i.e., lake connectivity measures) and topographic (i.e., lake watershed delineations) calculations or water quality data, some measures of ecological context may be missing. For example, for lakes in Canada, we were not able to estimate any data that relied on national datasets that stopped at the Canadian border; one exception is the NHD, which extends into Canada to retain hydrologic boundaries.

Data sources of the LAGOS-NE_{LOCUS} module

Detailed information on data sources are found in 'Additional File 5' in Soranno et al. [17]. Briefly, the data source for lakes and streams in the 17 state area was the NHD [21]. The hydrologic boundaries (i.e., for three of the spatial classifications, HUC12, HUC8, HUC4) came from the Watershed Boundary Dataset (WBD; [23]). In addition, we used the digital raster dataset of elevation for watershed delineation from the National Elevation Dataset [24]. All download dates for these data sources are provided in 'Additional File 5' in the above citation.

Data-integration methods of the LAGOS-NE_{LOCUS} module

All methods to create this module are described in Soranno et al. [17]. The most challenging and time-consuming part of building this module was connecting the sampling locations from the lake water quality datasets (which each contained different types of unique identifiers, and sometimes only lake names) to a georeferenced location in the NHD. When data providers included the lake latitude and longitude, we were able to mostly automate the procedure. Nevertheless, even when coordinates were available, there were many cases where the latitude and longitude did not intersect the NHD lake polygon boundary, requiring manual interpretation.

Figure 3. Examples lake watersheds (IWS) in LAGOS-NE. The watersheds are coded by hydrologic class to which its lake belongs. Data are from the LAGOS-NE_{GEO} v.1.01 data module and the GIS data coverages.

Quality Control of the LAGOS-NE_{LOCUS} module

The full description of error analysis for this module is described in Soranno et al. [17]. However, here we briefly describe our efforts to determine the minimum area of a lake that we could confidently represent using the NHD (further details located in Additional File 9 in Soranno et al. [17]). Although the NHD is a national dataset, it is updated and edited regionally (often at the state level) by local

practitioners familiar with each study region. As a result, there are regional differences in the resolution and digitization of water bodies, particularly for small water bodies, making it difficult to quantify or document even nominal error rates, or rather, the minimum lake size that is well-represented in the NHD. It has been documented previously that the NHD may not successfully identify small water bodies due to a variety of reasons including the resolution of the original underlying data of the NHD database, errors in digitization, hydrologic changes since the time of map creation (e.g., [25, 26]). Because of these documented issues, some programs have set minimum lake area cutoffs for sampling lakes. Most notable is the EPA-National Lakes Assessment of 2007, which chose a minimum size of 4 ha; although a smaller size cutoff was chosen for the EPA-National Lakes Assessment of 2012 [27]. To determine an appropriate size cutoff for our purposes, we conducted an analysis to identify the lakes that are best represented by the NHD across the LAGOS-NE study area.

We selected four states (WI, MI, IA, ME) in which to evaluate error rates of water body identification for lakes ≥ 1 ha and seven states (WI, MI, IA, ME, MO, NH, OH) in which to evaluate error rates for lakes ≥ 4 ha. We randomly selected three 100 km2 rectangles from each state then compared the number of lakes occurring in the NHD GIS coverage to the number of lakes in the best available aerial imagery from a range of sources to calculate the percentage of lakes missing from the NHD. The average percentage of lakes missing from the NHD was 58% for the ≥ 1 ha four-state test and 13% for the ≥ 4 ha seven-state test. Because an average of 87% of lakes ≥ 4 ha that are present in high-resolution aerial imagery are also present in the NHD, we chose this surface area as our cut-off and accepted this error rate.

Data in the LAGOS-NE$_{LOCUS}$ module

Figure 1 shows the census population of all lakes ≥4 ha in the 17-state area, including border areas beyond the 17-state boundary. As expected, the lakes are not evenly distributed, with higher densities in the northern parts of the study area. For those lakes with known lake depth (9,808 lakes with maximum depth values, and 4,090 lakes with mean depth values), there is little regional pattern of lake depth; shallow and deep lakes are found throughout the study area (see [28] for further details). Watershed size varies greatly across the study extent, reflecting the wide range of different lake hydrologic types and connections to upstream water bodies (Figure 3). In fact, the proportion of lakes in different lake hydrologic connectivity classes varies regionally across our study extent (Table 2; see [29] for further details).

Table 2. Numbers of lakes in each state by lake hydrologic class

State	Lakes ≥ 4 ha (#)	Isolated Lakes (#)	Headwater lakes (#)	Drainage lakes (#)	Drainage lakes with upstream lakes (#)
Connecticut	770	40	119	424	187
Illinois	2,831	1,417	279	952	183
Indiana	1,883	760	244	697	182
Iowa	915	339	87	402	87
Maine	2,661	94	619	1,211	737
Massachusetts	1,716	210	269	751	486
Michigan	6,531	2,649	1,087	1,672	1,123
Minnesota	14,031	6,609	1,894	2,673	2,855
Missouri	1,865	435	179	1,113	138
New Hampshire	1,118	70	224	581	243
New Jersey	1,148	219	129	521	279
New York	4,477	629	1,210	1,915	723
Ohio	1,282	543	105	520	114
Pennsylvania	1,757	316	397	840	204
Rhode Island	266	35	40	115	76
Vermont	531	14	74	364	79
Wisconsin	6,026	2,982	823	1,236	985
**Total**	**49,808**	**17,361**	**7,779**	**15,987**	**8,681**

The number of lakes ≥ 4 ha in each of the lake hydrologic classes by state, as well as the total numbers of lakes by hydrologic class calculated for the study extent. Note, in this table, lakes are counted for each state in which they occur (i.e., lakes that straddle two states are counted in both states).

5. Description of LAGOS-NE$_{LIMNO}$ v1.087.1 data module

The LAGOS-NE$_{LIMNO}$ module includes in situ measurements of lake water quality. We included variables that are most commonly measured by state agencies and researchers for studying eutrophication (Figure 2, variables labelled as **Water quality variables**). For each water quality data value, we also include metadata as additional columns in the exported data table (Figure 2, variables labelled as **Metadata**) including: the date of the sample, the name of the sampling program, the analytical methods, qualifiers with data flags from the original program (*qual*, which is not standardized for LAGOS-NE), detection limits (if available), and standardized censor codes from our quality control procedures (*censorcode*, standardized for LAGOS-NE).

Data sources of the LAGOS-NE$_{LIMNO}$ module

We acquired individual water quality datasets for LAGOS-NE$_{LIMNO}$ by contacting individuals at each of the 17 state and 5 tribal agencies. These contacts helped us to identify the state-agency collected dataset required by the Clean Water Act and which is most likely to be in the public domain. In this way, we were able to acquire at least one (and typically more) dataset from each of the 17 states. Because state and tribal agencies vary in sampling approach and intensity (see below for details), we sought to supplement these datasets with other known sources of water quality data, including university researchers, federal agencies, and non-profit groups to integrate into the LAGOS-NE$_{LIMNO}$ module. The full list of data sources acquired is in Soranno et al. [17] in 'Additional File 17'; however, we incorporated a subset of these datasets in LAGOS-NE$_{LIMNO}$ v1.087.1 (the data file *LAGOSNE_source_program_10871.csv* contains the list of sources for this version of LAGOS-NE).

Data-integration methods of the LAGOS-NE$_{LIMNO}$ module

All methods to create this module are described in Soranno et al. [17]. Briefly, for each dataset acquired, we authored LAGOS-NE metadata in EML to aid in data provenance (included in this paper). We also incorporated key metadata features (e.g., methods used, censor codes (if applicable)), and sampling program information) into the database so that future users could easily identify these important attributes. Because each dataset was unique in structure, file format, and naming conventions, we manually processed each dataset and its metadata so that they could be translated into the standard LAGOS-NE vocabulary and data model. Although labor-intensive, we created customized R scripts to process and load each dataset separately (included in this data paper).

Quality control of the LAGOS-NE$_{LIMNO}$ module

The full description of our QAQC procedures for this module are described in Additional File 2. Here, we provide a brief overview of our approach. Our goal for this effort was to identify egregiously high values and values that might be too low, both defined below. Note that our quality control procedures were not designed to identify statistical outliers, which individual users are expected to perform themselves because such analyses depend on the subsequent statistical analysis of each user. There were three major phases in the quality assurance/quality control (QAQC) procedure for LAGOS-NE$_{LIMNO}$. Phases I and II were designed to identify the egregious values that we defined as those that: 1) did not make ecological sense, 2) were far beyond what has been detected in previous studies, 3) were not technically feasible (e.g., SRP > TP), or 4) were a result of a data or file corruption or error in the data loading stage. For these egregious values, we explored the issues that might be underlying the values and removed them from the LAGOS-NE$_{LIMNO}$ data export provided in this data paper because we did had sufficient evidence that they were not scientifically valid data values. We were very conservative in these assessments to avoid removing data values that were high, yet still valid. Phase III was designed to identify and flag values that were lower than analytically possible (i.e., below detection limits) when there was sufficient metadata; however, note that these data are still provided in this data paper because it is not appropriate to remove data that are below detection.

For all versions of LAGOS-NE_{LIMNO}, Phase I and II are conducted on the entire cumulative dataset to leverage as large of a sample size as possible to detect problem values. In other words, because many of the QAQC analyses outlined here make use of all information from an individual lake or variable, incorporating new data may result in a better assessment of the data than when there is less data. Thus, for each new version of LAGOS-NE_{LIMNO}, new decisions are made about egregious values. In this data paper and this document, we describe the procedures for assessing all major versions of LAGOS-NE_{LIMNO}, but we present the results only for this version of LAGOS-NE_{LIMNO} (v1.087.1).

Because there are few accepted practices for conducting such quality control on a large, integrated database, we created our own procedures for Phase I and II by creating tests to identify egregious values that leverage a large, integrated database with multiple measures of water quality and well-established expected relationships among variables. The database that we used to identify egregious values was based on data in the full LAGOS-NE_{LIMNO} database for samples taken from all lake depths provided by the source datasets (note, our data exports in this data paper are only for epilimnetic or surface samples). While the quality control procedures that we implemented here were designed to help resolve the large and egregious errors in a combined dataset such as this, there are likely additional extreme values in the database due to the size and heterogeneity of the data. Users may want to check for additional issues in the data values specific to their proposed analyses.

Data in the LAGOS-NE_{LIMNO} module

All data in LAGOS-NE_{LIMNO} v1.087.1 are from samples that we identified as being collected from either the lake surface or the epilimnion (the well-mixed surface layer of a thermally-stratified lake during the period of stratification). Because we did not have lake temperature data to quantify the exact epilimnion depth in all lakes, we used information from the source datasets to either determine epilimnion depth, or to select data from only the top water layers. Although we received data from different depths in lakes, the majority of the samples were from the surface or epilimnion. The database includes samples from any season of the year. However, most of the published analyses to date have focused on the summer stratified period.

Lakes are not sampled the same way by all individuals, groups, or agencies; there are differences in the variables measured, the frequency and timing of sampling, and the proportion of lakes sampled. For example, for total phosphorus, the four states with the largest number of unique lakes with at least one value for total phosphorus per state include: Wisconsin (1,920 lakes), Minnesota (1,588), New York (1,289), and Michigan (1,109) (Table 3). However, the states with the highest proportion of their lakes with total phosphorus samples are the smaller states with fewer numbers of lakes, such as New Hampshire (64%), Vermont (58%), and Rhode Island (42%). Notably, there are some states with intermediate numbers of lakes that still have quite large percentages of their lakes with total phosphorus values, including Maine (35% of 2,645 lakes), Wisconsin (32% of 6,009 lakes), and New York (29% of the 4,461 lakes).

The most commonly measured variable in LAGOS-NE_{LIMNO} is water clarity measured as Secchi depth (a relatively easy and cost-effective measure of water quality), with 897,724 measurements taken from 12,034 unique lakes in the 17 states from mostly the mid 1980's to 2011 (Table 3). The second and third most sampled measures of water quality are chlorophyll *a* and total phosphorus, respectively. Although it appears that total nitrogen is sampled far less frequently than total phosphorus, some labs measure total nitrogen directly and report that single value, whereas other labs measure the constituents that make up total nitrogen (total Kjeldahl nitrogen and nitrate+nitrite), and sum them together to calculate total nitrogen. All of our analyses conducted on total nitrogen have used such calculated and measured values of nitrogen together, which increase the sample sizes for total nitrogen markedly.

Most of our data came from state agencies, either alone or as part of joint programs with citizen scientists or university researchers (Table 4); which highlights the importance of citizen science programs for monitoring lake water quality in this lake-rich area of the U.S.

Table 3. Summary of the water quality variables and the number of values per variable by state.

State	Number of lakes (≥4 ha)	Variable	Total phoshporus	Secchi depth	Chlorophyll a	True color	Apparent color	Dissolved organic carbon	Total nitrogen	Total Kjeldahl nitrogen	Nitrate + nitrite
Connecticut	763	# of samples:	1294	1943	1160	53	0	74	853	55	397
		# of sampled lakes:	143	168	149	37	0	49	99	26	81
		sample years:	1972-2010	1937-2010	1937-2013	1984-2007	n/a	1984-2007	1973-2010	1999-2009	1976-2010
Illinois	2819	# of samples:	2816	2317	1438	20	0	20	43	1526	2351
		# of sampled lakes:	191	185	167	17	0	17	18	155	188
		sample years:	1999-2011	1999-2011	2000-2011	2007	n/a	2007	2001-2009	1999-2006	1999-2009
Indiana	1874	# of samples:	1232	1303	909	57	0	57	57	1183	1237
		# of sampled lakes:	341	340	320	51	0	51	51	322	341
		sample years:	1988-2010	1986-2010	1990-2009	2007	n/a	2007	2007	1988-2009	1988-2009
Iowa	903	# of samples:	2873	2836	2711	18	0	18	2244	6	2229
		# of sampled lakes:	111	111	103	12	0	16	111	1	111
		sample years:	1997-2011	1997-2011	1997-2011	2007	n/a	2007	2001-2011	2008-2009	2001-2011
Maine	2645	# of samples:	17314	83472	12480	1927	1676	3321	1260	8	1577
		# of sampled lakes:	933	1047	793	601	466	848	461	3	347
		sample years:	1971-2011	1952-2011	1974-2011	1983-2011	1972-2011	1984-2011	1995-2011	1978-1993	1978-2011
Massachusetts	1698	# of samples:	570	760	326	277	228	300	69	69	351
		# of sampled lakes:	211	249	122	122	89	140	37	4	132
		sample years:	1978-2013	1978-2010	1986-2010	1984-2013	1978-2010	1984-2010	2000-2010	1978-2013	1978-2013
Michigan	6511	# of samples:	10143	95283	12243	1811	69	987	749	2651	4850
		# of sampled lakes:	1109	1233	862	836	69	353	200	713	948
		sample years:	1965-2013	1925-2013	1959-2013	1973-2010	2002-2003	1984-2013	1959-2011	1980-2010	1973-2012
Minnesota	13984	# of samples:	10974	497646	81925	406	6683	3382	7717	43054	7725
		# of sampled lakes:	1588	4118	2755	253	1368	811	619	2018	1522
		sample years:	1944-2011	1938-2012	1970-2012	1981-2009	1949-2011	1984-2012	1945-2012	1944-2012	1945-2012
Missouri	1858	# of samples:	11619	11794	11578	27	0	27	11340	0	27
		# of sampled lakes:	208	207	201	23	0	23	207	0	23
		sample years:	1978-2013	1978-2013	1978-2013	2007	n/a	2007	1978-2013	n/a	2007
New Hampshire	1109	# of samples:	9289	2958	154	237	3044	390	22	1209	2445
		# of sampled lakes:	710	618	21	111	603	143	17	535	704
		sample years:	1975-2013	1975-2011	1983-2012	1984-2010	1975-2010	1984-2010	2004-2010	1975-1994	1975-2013
New Jersey	1143	# of samples:	421	461	446	27	0	44	10	443	472
		# of sampled lakes:	175	174	157	25	0	36	8	157	175
		sample years:	1984-2009	1984-2009	2005-2009	1984-2007	n/a	1984-2007	2007	2005-2009	1984-2009
New York	4461	# of samples:	21356	21235	21000	27297	2287	13036	8259	944	27796
		# of sampled lakes:	1289	693	545	1421	47	1158	258	279	1279
		sample years:	1975-2012	1975-2012	1975-2012	1981-2012	1984-2011	1982-2011	1990-2012	1981-2010	1975-2012
Ohio	1279	# of samples:	377	1868	1912	20	0	220	1873	0	447
		# of sampled lakes:	144	144	137	19	0	44	145	0	40
		sample years:	2006-2007	1992-2010	1992-2010	2007	n/a	2006-2010	1994-2010	n/a	1993-2007
Pennsylvania	1755	# of samples:	1170	924	971	163	0	160	638	16	290
		# of sampled lakes:	263	260	160	124	0	124	167	2	147
		sample years:	1980-2011	1984-2011	1980-2011	1984-2008	n/a	1984-2007	1997-2011	1985-2010	1980-2010

Rhode Island	253	# of samples:	3325	18211	12195	51	6	65	2582	0	2100
		# of sampled lakes:	106	107	102	27	1	32	99	0	102
		sample years:	1984-2010	1984-2010	1986-2010	1984-2007	2003-2010	1984-2010	1992-2010	n/a	1984-2010
Vermont	528	# of samples:	13906	23894	15273	1774	1542	982	8	194	2271
		# of sampled lakes:	307	301	249	94	82	83	8	2	116
		sample years:	1977-2010	1977-2010	1977-2010	1981-2010	1979-2010	1984-2010	2007	1979-1994	1977-2010
Wisconsin	6009	# of samples:	45973	130819	26068	4599	174	4029	1932	9596	9417
		# of sampled lakes:	1920	2079	1024	1281	1	671	180	1160	1216
		sample years:	1933-2013	1948-2013	1933-2013	1974-2013	1976-1998	1977-2013	1986-2010	1933-2013	1965-2013
**TOTAL**	49592	**# of samples:**	**154652**	**897724**	**202789**	**38764**	**15709**	**27112**	**39656**	**60954**	**65982**
		**# of sampled lakes:**	**9749**	**12034**	**7867**	**5054**	**2726**	**4599**	**2685**	**5377**	**7472**

We include the number of individual values (representing an individual sampling event); the number of unique lakes for which there is at least one data value; and, the earliest and most recent year of sampling, all recorded by state and variable from any time period. Additional variables in LAGOS-NE$_{LIMNO}$ v1.087.1, not included in this table, which have relatively low sample sizes include: dissolved Kjeldahl nitrogen, ammonium, nitrite, soluble reactive phosphorus, total dissolved nitrogen, total dissolved phosphorus, total organic carbon, and total organic nitrogen.

Table 4. **The number of datasets, data values, and lakes from the different types of sampling programs in LAGOS-NE v1.087.1.**

Program Type	Number of datasets	Number of lakes (≥4 ha)		Total phosphorus	Secchi depth	Chl. a	True color	Apparent color	Dissolved organic carbon	Total nitrogen	Total Kjeldahl nitrogen	Nitrate + nitrite
Federal Agency	3	17	# of values:	419	527	324	229	173	215	335	6	30
			# of unique lakes:	17	17	17	13	15	14	16	1	9
Federal Agency/ University	2	2	# of values:	-	799	-	-	-	-	-	-	-
			# of unique lakes:	-	2	-	-	-	-	-	-	-
LTER	3	9	# of values:	2,346	3,529	2,567	-	-	1,872	1,612	507	2,396
			# of unique lakes:	9	9	5	-	-	9	9	4	9
National Survey Program	5	2,244	# of values:	2,320	2,595	243	3,689	703	4,714	431	-	4,204
			# of unique lakes:	1,863	1,891	171	13	142	2,235	398	-	1,997
Non-Profit Agency	4	44	# of values:	1,326	4,798	2,678	-	-	-	214	9	908
			# of unique lakes:	44	41	28	-	-	-	39	1	44
State Agency	33	4,264	# of values:	34,348	42,888	29,993	16,240	5,010	14,528	5,359	7,220	25,684
			# of unique lakes:	3,914	3,186	2,309	2,092	776	1,191	634	1,991	3,216
State Agency/ Citizen Monitoring	11	7,039	# of values:	79,390	645,650	124,766	18,010	8,630	3,195	18,610	52,995	27,826
			# of unique lakes:	3,955	6,629	4,341	1,111	1,508	786	772	3,476	2,782
State Agency/Univ/ Citizen Monitoring	4	1,835	# of values:	31,809	194,177	37,993	439	1,171	1,519	10,844	-	2,112
			# of unique lakes:	1,439	1,812	1,253	302	393	574	712	-	99
Tribal Agency	5	46	# of values:	911	145	905	3	-	357	411	277	463
			# of unique lakes:	33	3	32	3	-	11	18	5	17
University	17	535	# of values:	2,273	4,412	3,939	172	69	723	2,275	-	2,397
			# of unique lakes:	326	500	415	151	69	318	396	-	171

Figure 4. Percentage of lakes by lake area with water quality data. Percentage of census lakes in each lake area bin (top panel) compared to the percentage of census lakes for which there are limnological data for Secchi (second panel), chlorophyll *a* (third panel), and total phosphorus (TP; bottom panel)

Using the three most sampled variables in the dataset (Secchi depth, chlorophyll concentration and total phosphorus), we found that larger lakes were more likely to be sampled for water quality than smaller lakes (Figure 4). This result was expected given the economic and recreational interest in larger lakes, including easier public access. Previous research has already documented this basic pattern in 6 of the states included in LAGOS-NE [30]. Across all states, almost 80% of lakes > 400 ha have water quality data.

Lakes are also unevenly sampled through time, depending on the variable (Figure 5). Some programs' focus is on long-term monitoring, whereas others are short-term initiatives. Typically, long-term monitoring programs are localized to a few lakes, although there are exceptions (e.g., monitoring for acid rain in the NE in the 1980's-present has resulted in good temporal and spatial coverage for some variables through time and space [31].

Figure 5. The number of years of water quality data by lake. The number of years for which at least one sample is taken during the summer stratified season (15 June to 15 September) for: Secchi depth in meters, total phosphorus in ug/L, total nitrogen in ug/L (includes both measured and calculated values), and chlorophyll a in ug/L.

6. Description of LAGOS-NE$_{GEO}$ v1.05 data module

The LAGOS-NE$_{GEO}$ module includes information on the ecological context of the census lakes, their watersheds, and their regions. The information provided in the data tables for this module is organized into three main themes in which data are exported into individual tables: CHAG - climate, hydrology, atmospheric deposition of nitrogen and sulfur, and surficial geology; LULC - land use/cover, impervious cover, canopy cover, slope and terrain indices, and dam density; and CONN - lake, stream, and wetland abundance and connectivity measures (Figure 2). We also provide the GIS coverages that include some of the underlying data for this module, including: lake polygons and their hydrologic classifications defined in [17]; wetland polygons and their classification; streams as a line coverage and their classification by stream order; the zones used for this study (state and county; hydrologic units [at the 4, 8 and 12 scales; [32]]); and, lake watersheds (IWS). We also include boundaries of U.S. states and Canadian provinces for mapping.

Data sources of the LAGOS-NE$_{GEO}$ module

Detailed information on data sources are found in 'Additional File 5' in Soranno et al. [17]. Almost all data sources for this module are from national-scale datasets and thus use standardized methods throughout the study extent.

Data-integration methods of the LAGOS-NE$_{GEO}$ module

All methods to create this module are described in 'Additional files 5, 7, 8, 13, and 14' in Soranno et al. [17]. Briefly, we calculated the metrics for this module that describe the ecological context surrounding lakes by developing project-specific GIS tools in the ArcGIS environment, which are referred to as the LAGOS GIS Toolbox (and made available here: [33]). The toolbox outputs multiple individual data tables of calculated values organized by the above three data themes that are then imported into LAGOS-NE$_{GEO}$ for different spatial classifications, including values calculated at the level of the individual lake, 100 m and 500 m buffers around each lake, the lake IWS, states and counties, hydrologic units, and ecological drainage units (an ecoregion spatial classification). The unique identifiers for this data module are the zone ID's for each spatial classification for which we calculate these metrics. In other words, we calculate land use around a lake in each of the zones of the many spatial classifications

in LAGOS-NE. However, the data are exported into individual tables by spatial classification. Therefore, there are different numbers of rows in each table; for example, there are 51,101 rows for the land use metrics calculated for the 100 m lake buffer because there are 51,101 lakes that have a 100 m buffer area, but only 17 rows for the land use metrics calculated for the state spatial classification.

Quality control of the LAGOS-NE$_{GEO}$ module

The full description of error analysis for this module is described in 'Additional file 14' in Soranno et al. [17]. Yhe quality control procedures for this module included procedures to identify possible errors or improbable values as a result of the extensive automated GIS data processing that creates the LAGOS-NE$_{GEO}$ data tables and to correct those problems. We assumed that the original data layers had already gone through extensive quality control by the originators of the datasets. We defined errors and improbable values to be: 1) values that did not make ecological sense; 2) values that were well beyond what has been observed in previous studies; 3) values that are not technically feasible; or, 4) null values that indicate an absence of data, when in fact data exist based on the input data coverages. Note, it was not our intention to remove statistical outliers that may or may not be real/true values. Rather, we conducted procedures on each exported table that included: verifying column headers and units, mapping the exported data to evaluate mapping extent and boundary issues using visual inspection, mapping the data distributions of each value, identifying values that were missing or zero, plotting distributions of the data, ensuring that proportions summed to 100 where relevant, and inspecting univariate plots of metrics that are known to be related (e.g., % urban land use versus % impervious surface).

Data in the LAGOS-NE$_{GEO}$ module

This module contains the largest amount of data of any of the modules. For example, Figure 6 shows the wide range of ecological context for the LAGOS-NE study area calculated for three different spatial classifications. For those variables that are measured coarsely (e.g., baseflow, runoff, atmospheric deposition, geology), we calculated variables for only the broader spatial classifications. For example, we did not calculate baseflow for spatial classifications finer than HUC12 because the underlying data for baseflow is estimated on a zone generally coarser than the area of a lake watershed.

Figure 6. **Example ecological context variables by spatial classification in LAGOS-NE.** The top four panels are zoomed in to selected regions of Minnesota and Wisconsin so that the zone boundaries can be seen. The upper left panel shows stream density in each lake IWS, and the upper right panel shows the percent of connected wetlands in each lake IWS. The middle left panel shows the 2011 percent urban land use/cover in each hydrologic unit code 12 (HUC12), and the middle right panel shows the 2011 percent agricultural land use/cover in each hydrologic unit code 12 (HUC12). The lower left panel shows the 2010 nitrogen deposition in each HUC8, and the lower right panel shows the average percent of streamflow that is baseflow in each HUC8.

7. Research to date using LAGOS-NE

Prior versions of this database have supported numerous peer-reviewed publications to date. In particular, LAGOS-NE is ideally suited for studying the local to regional controls of water quality through both space and time because of the large of number of lakes with in situ water-quality measurements and its wide gradients of ecological context. The lake census dataset also makes it possible to quantify the types of biases present in the dataset to assess the potential influence of uneven sampling efforts on results across both space and time. Below, we describe the types of research questions that have been and are being addressed using LAGOS-NE, organized according to three main topics related to studying water quality across space and time in thousands of lakes. We have published 10 articles using portions of this database, and 13 articles are in review or preparation presently.

Methods and database development for macrosystems ecology:

Several of our lines of research have required the development of novel methods and the application of existing methods in novel ways. Much of the impetus for this work on methods and database development has been driven by two needs. The first, was to further develop the database--i.e., creating derived and predicted data as a new data product that is publicly accessible (e.g., [28]). The second was to better understand the spatial and temporal distribution of data contained in LAGOS-NE and to further our understanding of important ecological attributes of lakes across multiple spatial scales. These two needs are not mutually exclusive--analyses that have helped contribute data to LAGOS-NE have also addressed important ecological questions.

Three data gaps were identified early during database development including: (1) a lack of lake depth information (lake depth drives many in-lake processes), (2) the need to develop a flexible method for creating ecological regions from multi-themed mapped data, which are often used in macroscale research to account for broad-scale patterns and processes and, (3) the need for developing ways to measure freshwater connectivity to account for the transport and processing of materials in lakes at broad scales. For the first gap, Oliver et al. [28] used a linear mixed model to predict lake depth for lakes where in situ measurements were lacking, allowing the relationship between surface area and lake depth to vary by region because of the strong regional differences in this relationship. Predictions in some regions were far better than other regions, potentially due to differences in underlying geomorphology. To address the second gap, Yuan et al. [34] developed a novel spatially constrained spectral clustering algorithm that balances geospatial homogeneity and region contiguity, to delineate ecological regions. Cheruvelil et al. [35] has since applied this clustering algorithm across the 17 state study region and tested the ability of newly developed regions to capture variation in lake nutrients and water clarity. Finally, to address the third gap, Fergus et al. [29] developed approaches for determining freshwater connectivity of lakes, streams, and wetlands across broad spatial extents. The resulting freshwater metrics and analysis provide insight into the spatial distribution of surface-water connectivity types across the LAGOS-NE study area and provide LAGOS-NE users with novel metrics of connectivity for use in future research.

A further challenge in large, integrated databases such as LAGOS-NE is the well-known problem with data derived from analytical methods related to the issue of detection limits [36]. Stow et al. (in prep) studied the in situ concentrations that were too low to be quantified by standard analytical practices — measurements that are termed left-censored or below a detection limit of an analytical method. Unfortunately, detection limits were only sometimes reported (although, we do include those data in LAGOS-NE$_{LIMNO}$ where available). In some cases, low values were flagged as being censored, with an explanation as to the reason for censoring the data value, but in other cases the reason for censoring was not clear. In some instances, patterns in the data suggested that ad hoc substitutions for censored observations may have occurred without clear documentation. Stow et al. (in prep) describe a statistical approach that can be used to accommodate left-censored data during macroscale statistical analyses. This work also led to refining how censored observations were reported in LAGOS-NE, which have been incorporated into all later versions of LAGOS-NE$_{LIMNO}$, including v1.087.1.

Lake water quality is affected by many ecological context features, such as lake physical characteristics, land cover, land use, and climate. The relationship between these features and the water-quality measurements are not always linear. In addition, the data tend to be noisy and often contain missing values, which makes it challenging to fit effective statistical models. To overcome these challenges, Yuan et al. [37] developed a novel algorithm for learning non-linear features to predict lake water quality. The algorithm also enables the missing values to be imputed in a way that preserves the relationship between the predictors and response variables. Furthermore, because many of the lake water-quality variables are strongly correlated with each other, their models are expected to be similar. This similarity information can thus be exploited to build better models especially for the lake water-quality variables that have very few observations because they are not sampled frequently. Yuan et al. (in prep) are developing a machine learning approach known as multi-task learning that can simultaneously build regression models of multiple lake water-quality variables for a large number of lakes, taking into account both the correlation between the variables and the spatial autocorrelation among the lakes. Because we expect many ecological datasets across broad geographic scales to have similar data gaps and challenges as LAGOS-NE, we think these methods will be extremely valuable for other researchers studying different macroscale questions.

Understanding spatial variation in lake nutrients and eutrophication at sub-continental scales:

LAGOS-NE allows investigation of spatial variation in lake nutrients and eutrophication at macroscales. For example, Lapierre et al. (in prep) identify general spatial principles that constrain relationships between ecosystem variables with different spatial structures. In other cases, specific questions regarding spatial patterns have focused on identifying important landscape controls on nutrients and their ratios [38], potential stress induced on phytoplankton communities by high nitrogen levels (Filstrup et al. in prep), and spatial autocorrelation in lake-specific relationships between chlorophyll and nutrients and carbon [39]. In addition, LAGOS-NE contains a wealth of information on a variety of lake ecosystem types. Shallow lakes, in particular, are very abundant across the study area and represent systems that can exhibit hysteresis in response to lake eutrophication. Cheruvelil and Wagner (in prep) are investigating the spatial distribution and temporal dynamics of water clarity in shallow lakes of the LAGOS-NE study area.

An important area of research, and one that was a motivating factor for the creation of LAGOS-NE, is understanding the importance of cross-scale interactions (CSIs) — where ecological processes operating at one spatial or temporal scale interact with processes operating at another scale — in lake ecosystems. Because of their importance ecologically and the challenge of quantifying them over large spatial extents, Wagner et al. [40] evaluated the statistical power of large multi-thematic, multi-scaled datasets, such as LAGOS-NE, to detect CSIs. This work not only helped inform the design of large-scale studies aimed at detecting CSIs, but also focused attention on the importance of considering CSI effect sizes and their ecological relevance.. To extend this work, Fergus et al. (in prep) are investigating the importance of both within- and cross-scale interactions in landscape models predicting lake nutrients, and the role that connectivity among freshwaters plays in these interactions. Understanding and predicting nutrients in lakes at macroscales is important to inform estimates of lake contributions to continental and global nutrient cycles. To date, much of this work has been performed on a nutrient-by-nutrient basis, despite knowing that cycles of nitrogen and phosphorus and other key elements are best understood by considering multiple elements in tandem, *e.g.,* in a stoichiometric framework [41] or through analysis of coupled biogeochemical cycles (e.g., [42, 43, 44]). Currently, efforts are underway to develop spatial joint nutrient distribution models to evaluate how our understanding of landscape-scale drivers of lake nutrients and predictive performance are improved by considering multiple nutrients simultaneously (multivariate models) compared to traditional univariate approaches that ignore that nutrient cycles can be tightly coupled in freshwaters (Wagner et al. in prep).

Understanding temporal and spatial variation in lake eutrophication at sub-continental scales:

In addition to the vast spatial data contained in LAGOS-NE, temporal data are available for many water-quality variables, and some of the ecological context variables (e.g., land use/cover and atmospheric deposition). This is important information within the context of understanding and predicting how lake ecosystems have and will respond to global change, such as changes in climate and land use, and management activities to reduce nutrient inputs to lakes. Because we do not expect responses to such change and actions to be the same everywhere, these questions must be addressed across both space and time. In particular, recent environmental changes and management efforts have been hypothesized to both improve and degrade water quality in lakes. However, to date, there have been no studies to examine these issues comprehensively across broad scales and to examine which drivers are most strongly related to eutrophication status in lakes. LAGOS-NE is very well suited to answer these types of questions.

For example, nearly 3,000 lakes were examined for trends in nutrients and chlorophyll from 1990 to 2013 using LAGOS-NE [45]. Across all lakes, nitrogen has declined, and phosphorus and chlorophyll have not changed. Nitrogen and stoichiometric changes in lakes were related to atmospheric deposition of nitrogen, providing key insight into large-scale nutrient transport and policies such as the Clean Air Act. Using only citizen-science data in a subset of the LAGOS-NE database, Lottig et al. [46] showed results that suggested little evidence for major declines or improvements in water quality. In addition, Collins et al. (in press) are examining the relationships between a wide range of climate metrics and water quality in ~11,000 lakes in LAGOS-NE to determine, 1) which climate metrics are most related to water quality; 2) whether physical, chemical and biological aspects of lakes respond to climate in the same way; and, 3) how the climate-water-quality relationship varies across space and regions with different ecological context. However, the temporal dynamics of lake ecosystem properties can sometimes be nonlinear and exhibit variability across the landscape--largely because of climate and within-lake processes. Lottig et al. (in prep) have developed models for understanding and predicting the often complex temporal patterns observed in water clarity. These studies point to the importance of considering both space and time when trying to understand broad-scale environmental issues in surface waters.

8. Using LAGOS-NE for future research, management, and policy

To facilitate potential future use of LAGOS-NE, we have thoroughly documented the database and its methods [17]; and, here, we share LAGOS-NE data with the broader research community. In this data paper, we include a wide range of research products, including: the water quality and ecological-context data; the GIS coverages underlying much of the analyses on freshwaters; and, an R package that facilitates use of LAGOS-NE [47]. This package includes functions to retrieve, store, and interact with the LAGOS-NE database that works across many different operating systems. The package should increase the ease with which users of the database are able to access the data and documentation while maintaining a reproducible workflow.

Key motives for constructing this database included interest in examining lake nutrients and productivity at multiple spatial and temporal scales, fostering broad-scale aquatic ecology and macrosystems research in an open-science platform, and providing new understanding and resources for management and policymakers. To this end, several team members have made presentations at scientific meetings about the structure and use of LAGOS-NE and subsets of LAGOS-NE data have been shared with other researchers and stakeholders and agency personnel in advance of this publication. These early uses of LAGOS-NE data by other researchers outside of our team include an investigation of patterns and causes of shifting distribution of a sentinel fish species (Rypel et al. in prep), developing models to simulate lake temperatures (Winslow et al. in prep) and fish species distributions, and developing a recruitment model for a popular game fish (Hansen et al. in prep). Results from the latter two efforts will inform state-level fisheries management as well as aid in prioritization of lakes for habitat conservation action across a tri-state region.

Much of the research that we and others are conducting with LAGOS-NE has implications for ecosystem management or environmental decision-making. In addition, we have collaborated with

boundary organizations and decision-makers. For example, under development is a dashboard of the ecosystem services provided by lakes for use by land managers (Keeler et al. in prep). In addition, we have helped the state of Michigan determine lake-specific nutrient standards [48]. Our hope is that this database and the associated support tools and documentation serve as a powerful resource and a foundation for future research and decision-making by a broad community of scientists, policy makers, and natural-resource managers. Indeed, our success and experience with database construction and research has inspired us to expand the spatial extent for LAGOS-NE. We have begun to build LAGOS-US, which will include similar data as LAGOS-NE but will be for the continental U.S.

9. Challenges and recommendations for creating large, integrated, and heterogeneous databases

We found that the largest challenge when creating this database was integrating many small heterogeneous datasets that had few common standards. Although creating such large, integrated datasets using fully automated procedures may happen someday, it appears that we are nowhere near such automation today. Until standards in metadata documentation and robust ontologies are created and widely adopted when creating local or regional datasets, future efforts to integrate these into larger databases will have to rely on close collaborations among domain experts and ecoinformatics professionals, extensive manual interpretation of individual datasets, and funds sufficient to implement these labor-intensive approaches [16]. Nevertheless, it is worth the time and money invested in database integration if the resulting databases support new research, management, policy, public outreach, and education at all levels. We anticipate that LAGOS-NE will serve as a foundation for new data modules that can be used beyond the original intent of LAGOS-NE.

<u>The economic value of water quality data in an integrated database</u>

This extensive effort was supported by a U.S. National Science Foundation grant that totaled $2.4 million, along with resources from other projects. Our team ranged in size from 14-20 individuals across the six years of the project, with many members compiling and integrating data, authoring metadata, creating new data products, and implementing quality control procedures, resulting in a tremendous number of person-hours. However, when one puts the cost of the data collection for the water quality data in the first place, the expense of this post-processing integration work is not as large as it sounds. Sprague et al. [16] suggest that a single sample (estimated for collecting nutrient or chemistry data from streams) ranged in cost from $2000-$6000 per sample. If we assume similar rates for lake sampling and multiply this cost by the total number of records of nutrient or chemical samples in LAGOS-NE (n=589,909), then the combined estimate to collect the water quality data found in LAGOS-NE is in the range of $1.2 - $3.5 billion. It cost us between 0.07 - 0.20 % of the cost to sample the data in the first place to harmonize these half a million records, and to build an ecological-context database for them. This relatively small investment in preserving, documenting, and harmonizing these valuable datasets creates the needed infrastructure for new broad-scaled research, management, education, and outreach uses.

<u>Strategies for broad-scale data-integration efforts:</u>

One challenge is to prioritize research areas which datasets that may benefit from a similar type of integration. State water quality datasets were an excellent source of quality data for integration and conducting broad-scaled research on aquatic systems. There are likely other such data sources that would benefit from being integrated as we have done here. We recommend the following strategies to make the best use of future data integration efforts.

(1) The database integration effort should be driven by key underlying research questions or goals, and grounded in a strong conceptual foundation of the important features to include. In our case, the principles of landscape limnology [18,19,20,12] guided the development of LAGOS-NE which helped us to

prioritize geospatial and lake features for inclusion in the database because the addition of any data type or dataset cost time and money.

(2) For databases with more than one major data type, it is very helpful to build the database in modular form, each with its own versioning system, specific data integration methods, and quality control procedures. This strategy was not a primary goal at the outset of our project, but, it emerged somewhat organically through the life of the project. We now recognize the many benefits that the modularity brings to the database, including making it much easier to be dynamic rather than static by providing a platform for the addition of new data, new types of data, and new modules in the future (such as for biological data, or data from high-frequency sensors).

(3) The entire process should be grounded in an open-science framework. Knowing that the database, design, and methods were to be shared and made usable by future users influenced our decisions throughout the process, and made documentation a high priority throughout.

(4) Creation of LAGOS-NE required a strong focus on team science, and in particular the roles of and incentives for early-career researchers in such efforts. This type of research cannot be conducted in a single-investigator mode, but requires a highly collaborative and effective team-based model (e.g., [49, 50, 51]). We explicitly considered strategies for ensuring that early-career team members get credit for their contributions [52]. We recommend providing these essential team members with opportunities for leadership, project management, personnel management, and intellectual growth. For example, they can be part of major decisions and can lead smaller efforts throughout the project, as well as be given power to shape team policies and practices. This integration of early-career researchers into the entire research team and effort will give early-career professionals deep knowledge of the database, the procedures, as well as the skills to conduct such work in the future.

(5) The decision how to dissemination the database documentation needs to be considered early in the project. For example, database documentation papers are rare, especially in ecology, but are very important. The dissemination of the documentation and procedural approaches for making this large, integrated, and heterogeneous database had to be published prior to making the database available [17] and prior to publication of research results stemming from LAGOS-NE because methods sections in journal articles are too short to include all the necessary documentation of such methods. Other researchers may be discouraged by the very real consequence that publishing such products take time and energy investments that may slow down production of research publications. However, such a paper was instrumental in supporting later research articles that used LAGOS-NE. Before making all of LAGOS-NE available to researchers, we supported open science by publishing subsets of LAGOS-NE data that were used in individual publications (e.g., [53, 54]). Therefore, we recommend that this (and other) database documentation papers become a more standard type of paper to describe the extensive methods involved and to supplement data papers. Such papers will facilitate the use, extension, and translation of these databases well into the future, as well as foster future research on broad-scale, complex, and societally-relevant environmental questions.

AVAILABILITY OF SUPPORTING DATA

The data sets supporting the results of this article are available in the Ecological Data Initiative repository, including the following specific components:

- LAGOS-NE-LOCUS v1.01: https://portals.edirepository.org/nis/mapbrowse?scope=edi&identifier=100
- LAGOS-NE-LIMNO v1.087.1: https://portals.edirepository.org/nis/mapbrowse?scope=edi&identifier=101
- LAGOS-NE-GEO v1.05 https://portals.edirepository.org/nis/mapbrowse?scope=edi&identifier=99

- An R package to access the data in this paper: https://github.com/cont-limno/LAGOS
- GIS coverages of the freshwater features that are linked to the data tables: https://portals.edirepository.org/nis/mapbrowse?scope=edi&identifier=98

- Individual water quality datasets and associated metadata for LAGOS-NE$_{\text{LIMNO}}$: url

DECLARATIONS

List of abbreviations

LAGOS-NE – LAke multi-scaled GeOSpatial and temporal database for the 17 Northeastern and Midwest U.S. states
GIS – Geographic Information System
US-EPA – United States Environmental Protection Agency
EML – Ecological Metadata Language
USGS – United States Geological Survey
NHD – National Hydrography Dataset
WBD – Watershed Boundary Dataset
IWS – Interlake Watershed
SRP – Soluble Reactive Phosphorus
TP – Total Phosphorus
TN – Total Nitrogen
DOC – Dissolved Organic Carbon
MAV – Maximum Allowable Value
TDN – Total Dissolved Nitrogen
IQR – Interquartile Range
LULC – Land Use Land Cover
CONN – Connectivity and abundance (lake, stream, and wetland)
CHAG – Climate, Hydrology, Atmospheric deposition of nitrogen and sulfur, and surficial Geology
HUC – Hydrologic Unit Code
CSI – Cross Scale Interactions

Competing interesting

The authors declare they have no competing interests

Funding

The creation of LAGOS-NE was supported by:
The National Science Foundation MacroSystems Biology Program in the Emerging Frontiers Division of the Biological Sciences Directorate (EF-1065786, EF-1638679, EF-1065649, EF-1065818, EF-1638554) and the USDA National Institute of Food and Agriculture, Hatch project 176820 to PAS. KEW thanks the STRIVE Programme (2011-W-FS-7) from the Environmental Protection Agency, Ireland. SMC thanks the NSF Division of Biological Infrastructure (1401954).

The water quality data that are incorporated into LAGOS-NE were originally funded by the following sources:
State of Maine; Michigan Agricultural Experiment Station; Fisheries Division, Michigan Department of Natural Resources; New York State Division of Water Quality; Wisconsin Department of Natural Resources; University of Wisconsin-Madison; State/Trust; Michigan State University Agriculture Experimental Station Disciplinary Research Grant Program; US EPA; US EPA Section 106/319 Grants; Tribal General Fund; U.S. Army Corps of Engineers Federal Lakes Operation & Maintenance Funds; Aquatic Plant Management Society; Aquatic Ecosystem Restoration Foundation; Michigan State

University; Michigan State University Department of Fisheries and Wildlife; EPA Star Fellowship to K.S.C. (U-915342-01-0); Andrew W. Mellon Foundation; Federal Aid in Sport Fish Restoration Program (Grant F-69-P, Fish Management in Ohio) administered jointly by the U.S. Fish and Wildlife Service and the Ohio Department of Natural Resources, Division of Wildlife; Iowa Department of Natural Resources (Contract #ESD04HALFasch110155); Minnesota Pollution Control Agency; NSF-Division of Environmental Biology; Ohio DNR Division of Wildlife; University of Rhode Island Watershed Watch; NSF Kellogg Biological Station Long Term Ecological Research (LTER) Program, DEB 1027253; NSF North Temperate Lakes LTER Program, DEB 1440297; Lac du Flambeau Band and Bureau of Indian Affairs; Indiana Department of Environmental Management; Missouri Department of Natural Resources; Clean Water Act Section 16; Michigan Department of Environmental Quality; Massachusetts Water Supply Protection Trust; US EPA Clean Air Markets Division (LTM Network); US EPA Office of Research and Development; New York City Department of Environmental Protection (NYSDEP); City of New York; USGS Water Availability and Use Science Program (WAUSP); U.S. Geological Society; New York State Energy Research and Development Authority; National Institute of Food and Agriculture, U.S. Department of Agriculture, Hatch Grant 1003732; the New York State Department of Environmental Conservation; Lake Sunapee Protective Association; National Oceanic and Atmospheric Administration; Gull Lake Quality Organization; Clean Michigan Initiative; NSF grant DEB-1455461.

Authors' contributions

Data for the database were contributed by: LCB, MB, KEB, MGB, MTB, SRC, JWC, KSC, MC, JDC, JAD, JD, CTF, CSF, MJG, LTG, JDF, SKH, PCH, EH, CH, JRJ, KJH, LLJ, WWJ, JRJ, CMK, SAK, BL, JAL, YL, NRL, JAL, LJM, WHM, KEBM, PBN, SJN, MLP, DCP, AIP, DMP, POR, DOR, KMR, LGR, OS, NJS, PAS, NRS, EHS, JLS, JMT, TPT, MV, GW, KCW, KEW, JDW, and MKW. The idea to create the database was conceived by PAS and KSC. PAS coordinated the different activities across team-members to build LAGOS-NE. The database was designed by EGB, PNT, CG, and PAS; and, created and managed by EGB. The following authored metadata for the individual water quality data sets using information provided by the data providers: MTB, CKB, KSC, SMC, CEF, CTF, ENH, NRL, SKO, NKS, PAS, EHS, and KEW. CEF prepared the integrated LAGOS-NE metadata, and developed the protocols for authoring the EML metadata; and, CEF and CKB created EML metadata for the 87 water quality data sets. SKO wrote the final variables definitions for the integrated metadata. CG helped to prepare the needed metadata and documentation for loading the data in the data repository. Code for importing the datasets into the database was written by EGB, STC, NRL, and SY. JS and SBS performed geospatial analyses and created the LAGOS-GIS Toolbox. The conceptual foundation for measuring freshwater connectivity was led by CEF. SBS developed the methods to delineate lake watersheds. The quality control methods development and analysis on LAGOS-NE$_{LIMNO}$ were conducted by NRL; the quality control of LAGOS-NE$_{GIS}$ was led by CES and SMC, and conducted by CES, SMC, CEF, NKS, and KEW. The quality control of LAGOS-NE$_{LOCUS}$ was conducted by EGB. Many authors who were part of the database integration team wrote the technical documentation; JFL served as editor of these technical documents. Tables and figures were prepared by SMC, KBSK, JFL, NRL, ACP, NKS and PAS and edited by many of the contributing authors. SKO and JJS wrote the LAGOS-NE R package. NJS prepared the GIS data and its corresponding metadata. PAS coordinated the writing of the manuscript, and major parts of the manuscript were written by: PAS, KSC, SMC, JFL, NRL, SKO, JJS, EHS, PNT, TW, and SY. After the lead author, authors are listed alphabetically.

Competing Interests

The authors declare that they have no competing interests

Acknowledgments

We thank the contributions over the past several decades of many hundreds to thousands of governmental, tribal and citizen scientists whose efforts from lake sampling to water quality analysis to dataset compilation enabled LAGOS-NE to become a reality and a resource for the future. Specifically, we dedicate this paper to the memory of Jody Connor, whose three decades of innovative and science-driven lake management while working for the New Hampshire Department of Environmental Services generated meaningful contributions to the protection and restoration of lake quality and to lasting data legacies such as volunteer monitoring in the state. This is Great Lakes Environmental Research Laboratory contribution number xxxx. Any use of trade, firm, or product names is for descriptive purposes only and does not imply endorsement by the US Government.

References (Final Reference List is a separate document. Updated 31-March)

1. Carpenter SR, Caraco NR, Correll DL, Howarth RW, Sharpley AN, Smith VH. Nonpoint pollution of surface waters with phosphorus and nitrogen. Ecological Applications. 1998; 8:559-568.
2. Jaworski NA, Howarth RW, Hetling LJ. Atmospheric Deposition of Nitrogen Oxides onto the Landscape Contributes to Coastal Eutrophication in the Northeast United States. Environmental Science and Technology 1997; 31:1995–2004.
3. Bennett EM, Carpenter SR, Caraco NF. Human Impact on Erodible Phosphorus and Eutrophication: A Global Perspective. BioScience. 2001; 51:227-234.
4. Schindler DW. Recent advances in the understanding and management of eutrophication. Limnology and Oceanography. 2006; 51: 356–363.
5. Taranu ZE and Gregory-Eaves I. Quantifying relationships among phosphorus, agriculture, and lake depth at an inter-regional scale. Ecosystems. 2008; 11: 715-725.
6. Filstrup CT, Wagner T, Soranno PA, Stanley EH, Stow CA, Webster KE, Downing JA. Regional variability among nonlinear chlorophyll-phosphorus relationships in lakes. Limnology and Oceanography. 2014; doi: 10.4319/lo.2014.59.5.1691.
7. McCrackin ML, Jones HP, Jones PC, Moreno-Mateos D. Recovery of lakes and coastal marine ecosystems from eutrophication: A global meta-analysis. Limnology and Oceanography. 2016; doi: 10.1002/lno.10441.
8. Paerl HW, Otten TG, Joyner AR. Moving towards adaptive management of cyanotoxin-impaired water bodies. Microbial Biotechnology. 2016; doi: 10.1111/1751-7915.12383.
9. Schindler DW, Carpenter SR, Chapra SC, Hecky RE, Orihel, DM. Reducing Phosphorus to Curb Lake Eutrophication is a Success. Environmental Science & Technology. 2016; doi: 10.1021/acs.est.6b02204.
10. Fergus CE, Soranno PA, Cheruvelil KS, Bremigan MT. Multiscale landscape and wetland drivers of lake total phosphorus and water color. Limnology and Oceanography. 2011; doi: 10.4319/lo.2011.56.6.2127.
11. Soranno PA, Cheruvelil KS, Bissell EG, Bremigan MT, Downing JA, Fergus CE, Filstrup CT, Henry EN, Lottig NR, Stanley EH, Stow CA, Tan P-N, Wagner T, Webster KE. Cross-scale interactions: quantifying multi-scaled cause–effect relationships in macrosystems. Frontiers in Ecology and the Environment. 2014; 12:65–73.
12. Read EK, Patil VP, Oliver SK, Hetherington AL, Brentrup JA, Zwart JA, Winters KM, Corman JR, Nodine ER, Woolway RL, Dugan HA, Jaimes A, Santoso AB, Hong GS, Winslow LA, Hanson PC, Weathers KC. The importance of lake-specific characteristics for water quality across the continental United States. Ecological Applications. 2015; doi: 10.1890/14-0935.1.
13. Smith VH, Dodds WK, Havens KE, Engstrom DR, Paerl HW, Moss B, Likens GE. Comment: Cultural eutrophication of natural lakes in the United States is real and widespread. Limnology and Oceanography. 2014; 59: 2217-2225.
14. McDonald CP, Lottig NR, Stoddard, JL, Herlihy AT, Lehmann S, Paulsen SG, Peck DV, Pollard AI, Stevenson RJ. Comment on Bachmann et al. (2013): A nonrepresentative sample cannot describe the extent of cultural eutrophication of natural lakes in the United States. Limnology and Oceanography. 2014; 59: 2226-2230.
15. Stoddard JL, Sickle JV, Herlihy AT, Brahney J, Paulsen S, Peck DV, Mitchell R, Pollard AI. Continental-Scale Increase in Lake and Stream Phosphorus: Are Oligotrophic

Systems Disappearing in the United States? Environmental Science and Technology. 2016; doi: 10.1021/acs.est.5b05950.

16. Sprague LA, Oelsner GP, Argue DM. Challenges with secondary use of multi-source water-quality data in the United States. Water Research. 2017; 100: 252-261.
17. Soranno PA, Bissell EG, Cheruvelil KS, Christel ST, Collins SM, Fergus CE, Filstrup CT, Lapieree JF, Lottig NR, Oliver SK, Scott CE, Smith NJ, Stopyak S, Yuan S, Bremigan MT, Downing JA, Gries C, Henry EN, Skaff NK, Stanley EH, Stow CA, Tan PN, Wagner T, Webster KE. Building a multi-scaled geospatial temporal ecology database from disparate data sources: fostering open science and data reuse. GigaScience. 2015; doi: 10.1186/s13742-015-0067-4.
18. Magnuson JJ, Kratz, TK. Lakes in the landscape: approaches to regional limnology. International Association of Theoretical and Applied Limnology. 2000; 27: 74-87.
19. Wiens JA. Riverine landscapes: taking landscape ecology into the water. Freshwater Biology. 2002; doi: 10.1046/j.1365-2427.2002.00887.x.
20. Soranno PA, Cheruvelil KS, Webster KE, Bremigan MT, Wagner T, Stow CA. Using landscape limnology to classify freshwater ecosystems for multi-ecosystem management and conservation. BioScience. 2010; 60:440–454.
21. United States Geological Survey National Hydrography Dataset. Version 9.3. http://nhd.usgs.gov. Accessed 4 June 2015.
22. Zhang T, Soranno PA, Cheruvelil KS, Kramer DB, Bremigan MT, Ligmann-Zielinska A. Evaluating the effects of upstream lakes and wetlands on lake phosphorus concentrations using a spatially-explicit model. Landscape Ecology. 2012; doi: 10.1007/s10980-012-9762-z.
23. United States Geological Survey Watershed Boundary Dataset. https://nhd.usgs.gov/wbd.html. Downloaded in 2013.
24. National Elevation Dataset. http://ned.usgs.gov/. Accessed 11 March 2013.
25. US Environmental Protection Agency: National lakes assessment fact sheet. 2010. http://water.epa.gov/type/lakes/upload/nla_survey_fact_sheet.pdf. Accessed 4 June 2015.
26. US Environmental Protection Agency: National lakes assessment 2012: a fact sheet for communities. 2012. http://water.epa.gov/type/lakes/assessmonitor/lakessurvey/upload/NLA-2012-Fact-Sheet-for-Communities.pdf. Accessed 4 June 2015.
27. Environmental Protection Agency: National Lake Survey of 2012. 2012. https://www.epa.gov/national-aquatic-resource-surveys/nla. Accessed 4 June 2015.
28. Oliver SK, Soranno PA, Fergus CE, Wagner T, Winslow LA, Scott CE, Webster KE, Downing JA, Stanley EH. Prediction of lake depth across a 17-state region in the United States. Inland Waters. 2016; doi: 10.5268/IW-6.3957.
29. Fergus CE, Lapierre JF, Oliver S, Skaff N, Cheruvelil K, Soranno, P, Webster K, Scott C. The freshwater landscape: Lake, wetland, and stream abundance and connectivity at macroscales. Ecosphere. (in review).
30. Wagner T, Soranno PA, Cheruvelil KS, Renwick WH, Webster KE, Vaux P, Abbitt RJF. Quantifying sample biases of inland lake sampling programs in relation to lake surface area and land use/cover. Environmental Monitoring and Assessment. 2007; doi:10.1007/s10661-007-9883-z.
31. Strock KE, Saros JE, Nelson SJ, Birkel SD, Kahl JS, McDowell WH. Extreme weather years drive episodic changes in lake chemistry: Implications for recovery from sulfate

deposition and long-term trends in dissolved organic carbon. Biogeochemistry. 2016; 127:353–365.
32. Seaber PR, Kapinos FP, Knapp GL. Hydrologic unit maps: U.S. Geological Survey water-supply paper 2294. U.S.G.S. 1987. http://water.usgs.gov/GIS/huc.html.
33. Smith NJ, Soranno PA, Stopyak S. LAGOS-NE GIS Toolbox. GitHub repository. 2014. https://soranno.github.io/LAGOS_GIS_Toolbox/.
34. Yuan S, Tan PN, Cheruvelil KS, Collins SM, Soranno PA. Constrained Spectral Clustering for Regionalization: Exploring the Trade-off between Spatial Contiguity and Landscape Homogeneity. Data Science and Advanced Analytics. 2015; doi:10.1109/DSAA.2015.7344878.
35. Cheruvelil KS, Yuan S, Webster KE, Tan PN, Lapierre JF, Collins SM, Fergus CE, Scott C, Henry E, Soranno PA, Filstrup CT, Wagner T. Creating multithemed ecological regions for macroscale ecology: Testing a flexible, repeatable and accessible clustering method. Ecology and Evolution. (in press).
36. Helsel DR. Statistics for censored environmental data using Minitab and R, 2nd edition. John Wiley and Sons, New York. 2012. http://www.wiley.com/WileyCDA/WileyTitle/productCd-0470479884.html.
37. Yuan S, Tan PN, Cheruvelil KC, Fergus CE, Skaff NK, Soranno PA. Hash-Based Feature Learning for Incomplete Continuous-Valued Data. SIAM International Conference on Data Mining. 2017.
38. Collins SM, Oliver SK, Lapierre JF, Stanley EH, Jones JR, Wagner T, Soranno PA. Lake nutrient stoichiometry is less predictable than nutrient concentrations at regional and sub-continental scales. Ecological Applications. (in press).
39. Fergus CE, Finley AO, Soranno PA, Wagner T. Spatial variation in nutrient and water color effects on lake chlorophyll at macroscales. PLoS ONE. 2016; doi: 10.1371/journal.pone.0164592
40. Wagner T, Fergus EC, Stow CA, Cheruvelil KS, Soranno PA. The statistical power to detect cross-scale interactions at macroscales. Ecosphere. 2016; doi: 10.1002/ecs2.1417.
41. Sterner RW, Elser JJ. The Biology of Elements from Molecules to the Biosphere. Princeton University Press: Princeton and Oxford; 2002.
42. Rastetter, EB. Modeling coupled biogeochemical cycles. Frontiers in Ecology and the Environment. 2001; 9: 68-73.
43. Finzi AC, Austin AT, Cleland EE, Frey SD, Houlton BZ, Wallenstein MD. Responses and feedbacks of coupled biogeochemical cycles to climate change: examples from terrestrial ecosystems. Frontiers in Ecology and the Environment. 2011; 9:61-67.
44. Finlay JC, Small GE, Sterner RW. Human influences on nitrogen removal in lakes. Science. 2013; 342:247-250.
45. Oliver SK, Collins SM, Soranno PA, Wagner T, Stanley EH, Jones JR, Stow CA, Lottig NR. Unexpected stasis in a changing world: Lake nutrient and chlorophyll trends since 1990. Global Change Biology. (in review).
46. Lottig NR, Wagner T, Henry EN, Cheruvelil KS, Webster KE, Downing JA, Stow CA. Long-Term Citizen-Collected Data Reveal Geographical Patterns and Temporal Trends in Lake Water Clarity. PLoS ONE. 2014; doi: 10.1371/journal.pone.0095769.
47. Stachelek J, Oliver S, Masrour F. LAGOS: Tools for Interacting with the Lake Multi-scaled Geospatial and Temporal

Database. R package version 1.087.1. GitHub repository. 2017. https://github.com/cont-limno/LAGO.

48. Cheruvelil KS, Soranno P. Developing nutrient criteria in Michigan lakes: Revision, update, and validation of the lake-specific model for establishing expected nutrient conditions in Michigan lakes. Report to the Michigan Department of Environmental Quality. FY2012 205(j). 2015.
49. Cheruvelil KS, Soranno PA, Weathers KC, Hanson PC, Goring SJ, Filstrup CT, Read EK. Creating and maintaining high-performing collaborative research teams: the importance of diversity and interpersonal skills. Frontiers in Ecology and the Environment. 2014; 12:31–38.
50. Weathers KC, Hanson PC, Arzberger P, Brentrup J, Brookes JD, Carey CC, Gaiser E, Hamilton DP, Hong GS, Ibelings B, Istvánovics V, Jennings E, Kim B, Kratz T, Lin FP, Muraoka K, O'Reilly C, Piccolo C, Ryder E, Zhu G. The Global Lake Ecological Observatory Network (GLEON): The Evolution of grassroots network science. Bulletin of Limnology and Oceanography. 2013; 22:71-73.
51. Hanson PC, Weathers KC, Kratz TK. Networked lake Science: how the Global Lake Ecological Observatory (GLEON) Works to understand, predict, and communicate lake ecosystem response to global change. Inland Waters. 2016; doi: 10.5268/IW-6.4.904.
52. Goring S, Weathers KC, Dodds WK, Soranno PA, Sweet LC, Cheruvelil KS, Kominoski JS, Rüegg J, Thorn AM, Utz RM. Improving the culture of interdisciplinary collaboration in ecology by expanding measures of success. Frontiers in Ecology and the Environment. 2014; 14: 39-47.
53. Oliver SK, Soranno PA, Fergus CE, Wagner T, Winslow LA, Scott CE, Webster KE, Downing JA, Stanley EH. LAGOS – Predicted and observed maximum depth values for lakes in a 17-state region of the U.S. Long Term Ecological Research Network. 2015; doi:10.6073/pasta/f00a245fd9461529b8cd9d992d7e3a2f.
54. Fergus CE, Finley AO, Soranno PA, Wagner T. Spatial variation in nutrient and water color effects on lake chlorophyll at macroscales. Long-Term Ecological Research Network Data Portal. 2016; doi: 10.6073/pasta/0ebd2e4c0705706b77b359955bff44e1.

Figure 1. Map of the study extent of LAGOS-NE. Map includes 17 states in the upper midwest and northeastern U.S. outlined in white and 51,101 lakes ≥ 4 ha shown as blue polygons. Some lakes extend beyond state borders and are included in the database if it was possible to delineate their watersheds. Watershed boundaries rather than state boundaries were used for all analyses of lakes, streams and wetlands. The map is modified from [17].

Figure 2. LAGOS-NE data modules and version numbers. The data modules and versions that are included in LAGOS-NE and are available with this paper include: $\text{LAGOS-NE}_{\text{GEO}}$ v.1.05, $\text{LAGOS-NE}_{\text{LOCUS}}$ v.1.01 (note, that in Soranno et al. [17], this module was called LAGOS-lakes), and $\text{LAGOS-NE}_{\text{LIMNO}}$v.1.087.1. We include descriptions of the type of data that are included in each module; with the major categories of variables the same as those describing the data tables in Additional File 1. The black connectors among the modules show that the modules are connected to each other through common unique identifiers through the $\text{LAGOS-NE}_{\text{LOCUS}}$ module (either the unique lake ID or the zone ID). P is phosphorus, N is nitrogen, C is carbon, S is sulfur, atm is atmospheric, NHD is the National Hydrography Dataset, IWS is the interlake watershed, WBD is the Watershed Boundary Dataset, EDU is Ecological Drainage Unit. Figure is modified from Figure 1 in Soranno et al. [17].

Figure 3. Examples lake watersheds (IWS) in LAGOS-NE. The watersheds are coded by hydrologic class to which its lake belongs. Data are from the $\text{LAGOS-NE}_{\text{GEO}}$ v.1.01 data module and the GIS data coverages.

Figure 4. Percentage of lakes by lake area with water quality data. Percentage of census lakes in each lake area bin (top panel) compared to the percentage of census lakes for which there are limnological data for Secchi (second panel), chlorophyll *a* (third panel), and total phosphorus (TP; bottom panel)

Figure 5. The number of years of water quality data by lake. The number of years for which at least one sample is taken during the summer stratified season (15 June to 15 September) for: Secchi depth in meters, total phosphorus in ug/L, total nitrogen in ug/L (includes both measured and calculated values), and chlorophyll a in ug/L.

Figure 6. **Example ecological context variables by spatial classification in LAGOS-NE.** The top four panels are zoomed in to selected regions of Minnesota and Wisconsin so that the zone boundaries can be seen. The upper left panel shows stream density in each lake IWS, and the upper right panel shows the percent of connected wetlands in each lake IWS. The middle left panel shows the 2011 percent urban land use/cover in each hydrologic unit code 12 (HUC12), and the middle right panel shows the 2011 percent agricultural land use/cover in each hydrologic unit code 12 (HUC12). The lower left panel shows the 2010 nitrogen deposition in each HUC8, and the lower right panel shows the average percent of streamflow that is baseflow in each HUC8.

Figure1

Click here to download Figure Figure 1 Study Extent Lake Map_FINAL.png

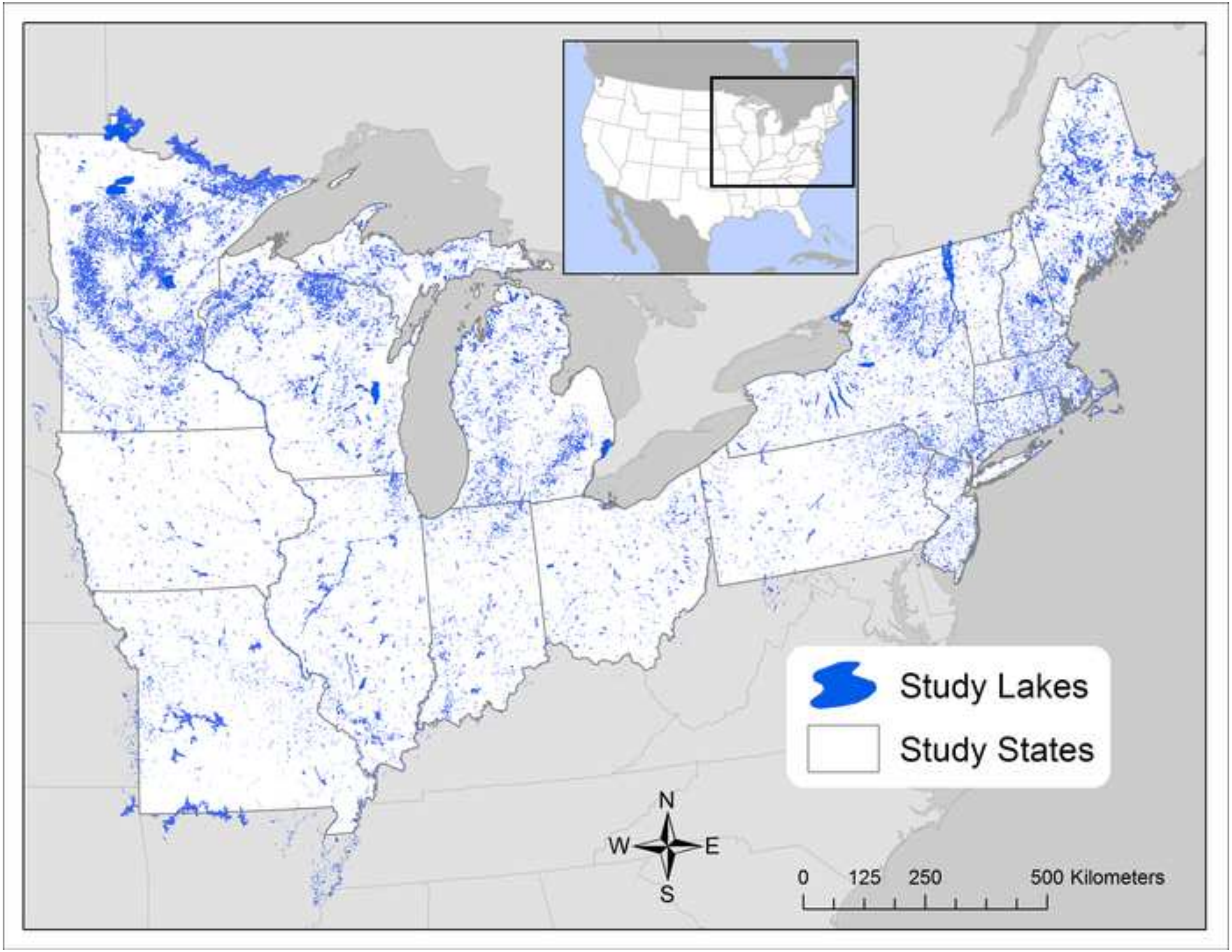

Figure2

Click here to download Figure Figure 2 - modules-final.png

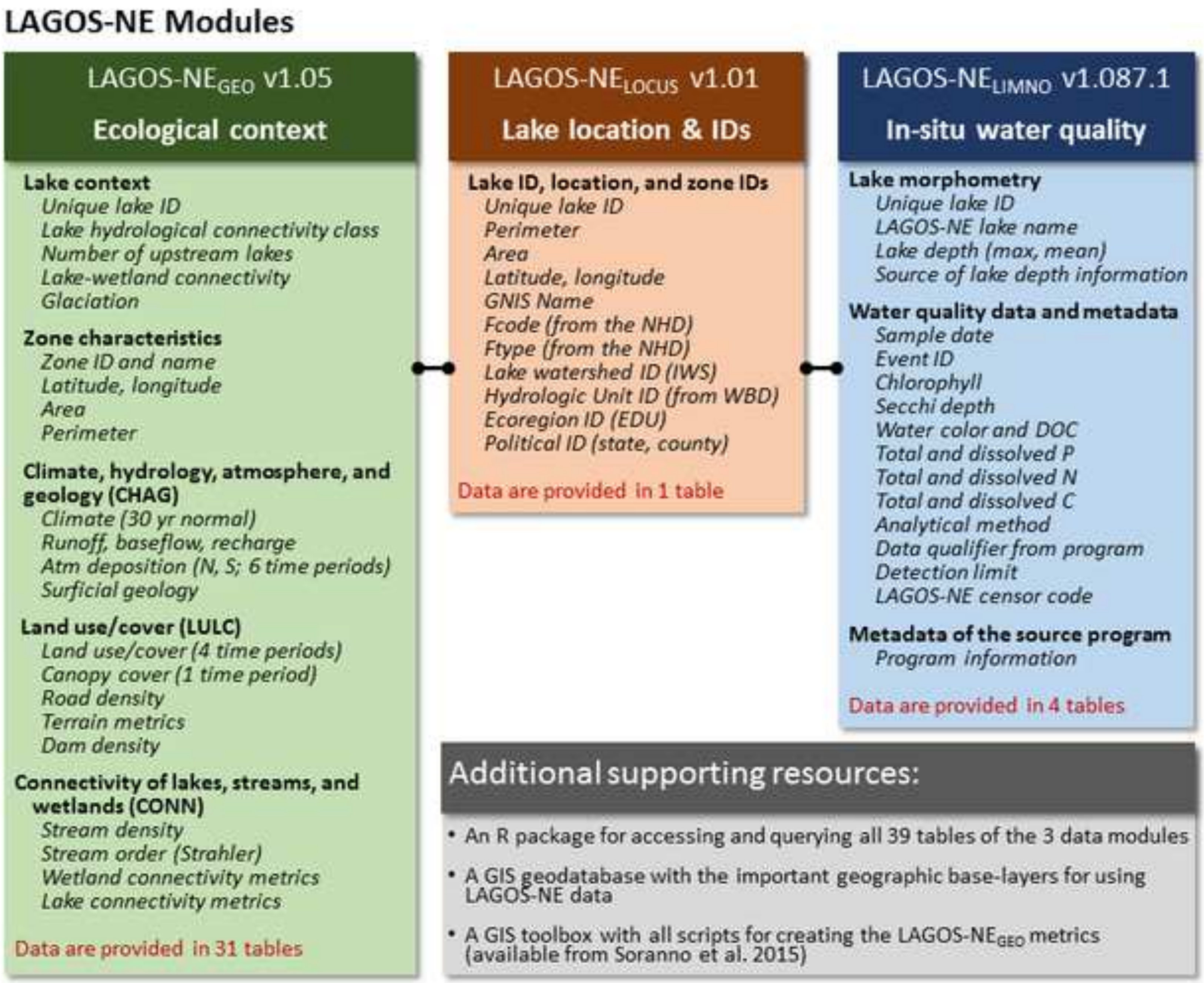

Figure3

Click here to download Figure Figure 3 IWS Hydro Class.png

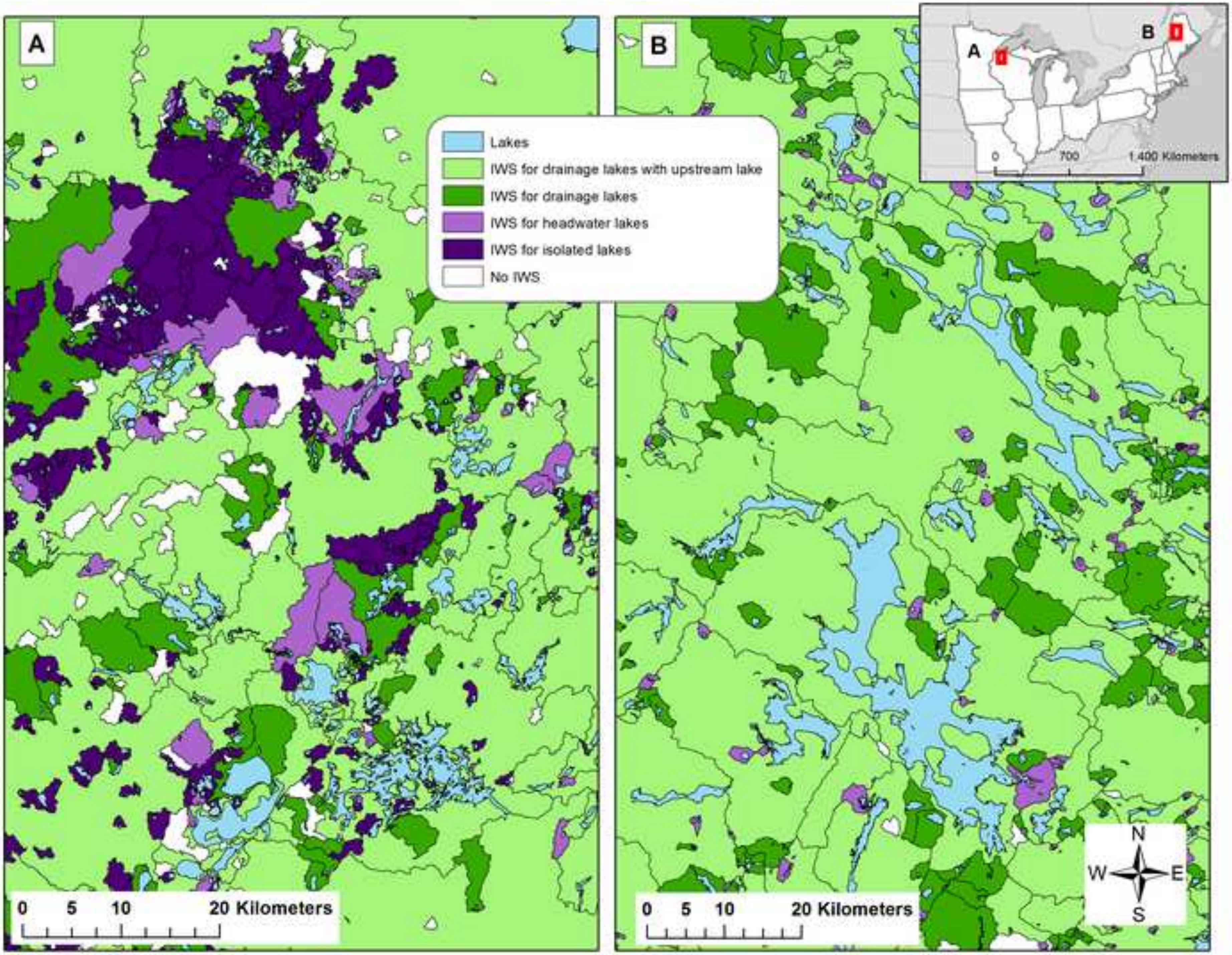

Figure4

Click here to download Figure Figure 4 CensusvsSampledLakeSize.png

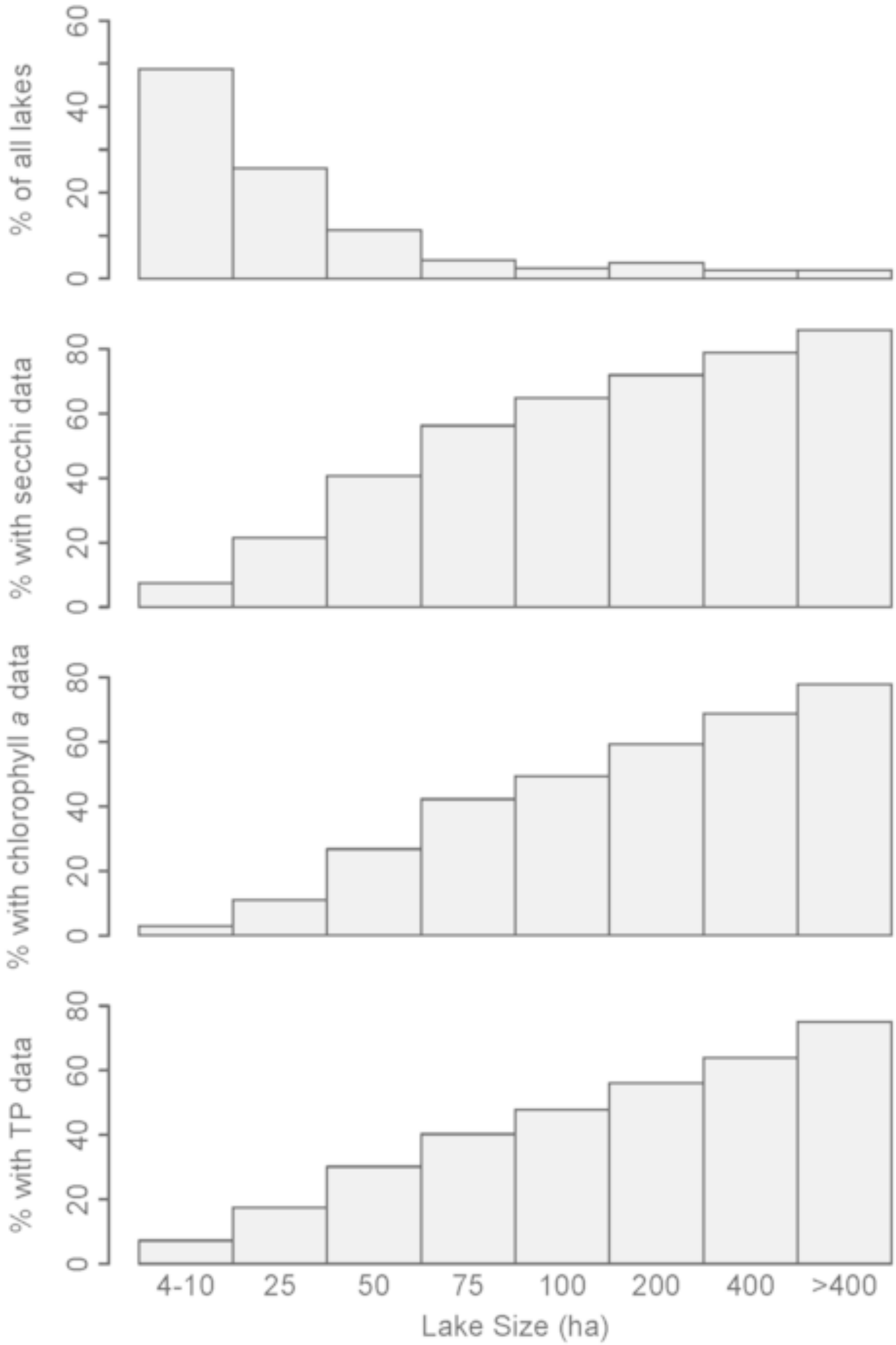

Figure5

Click here to download Figure Figure 5_TN_TP_CHL_SEC_years.png

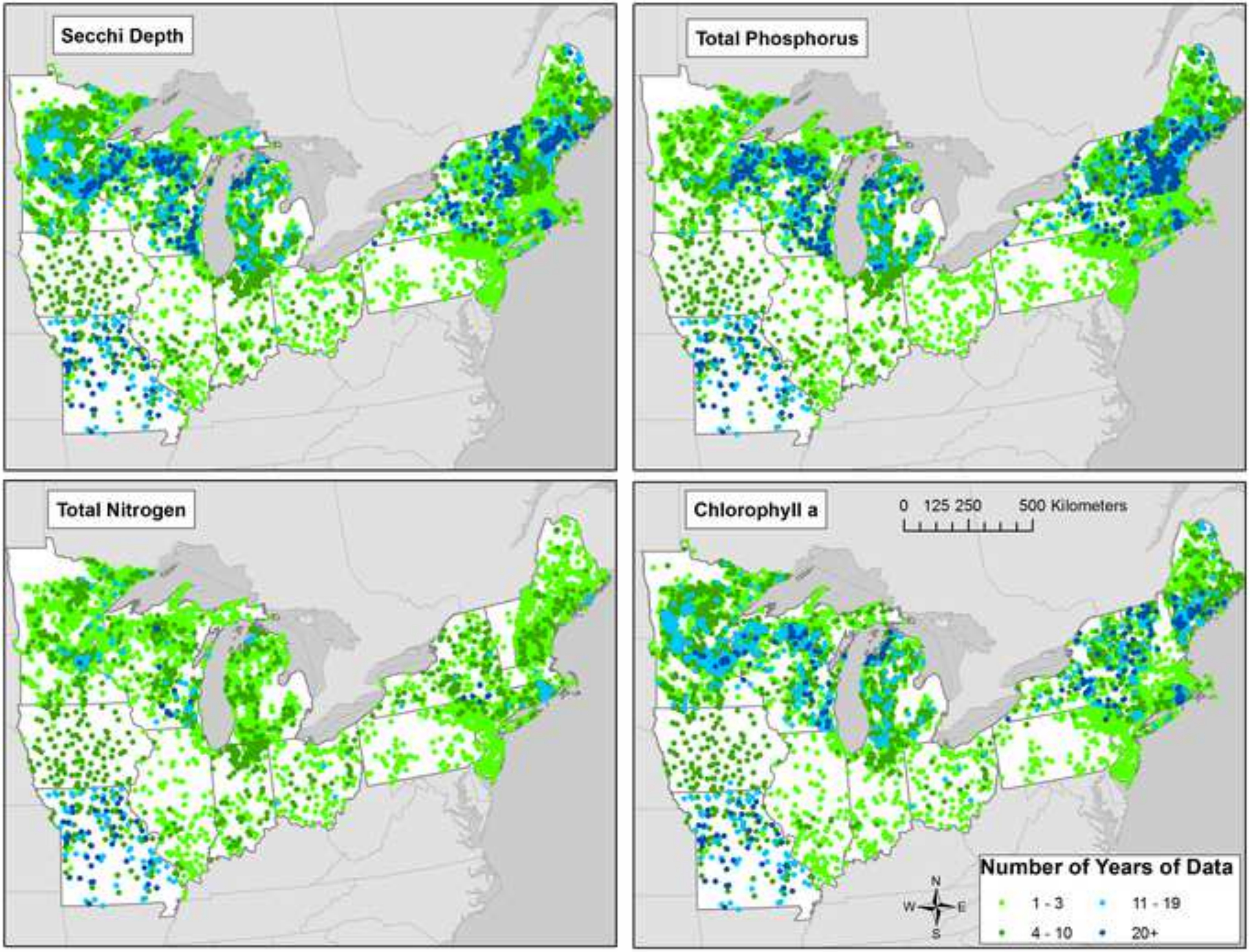

Click here to download Figure Figure 6 spatial extents_lines.png

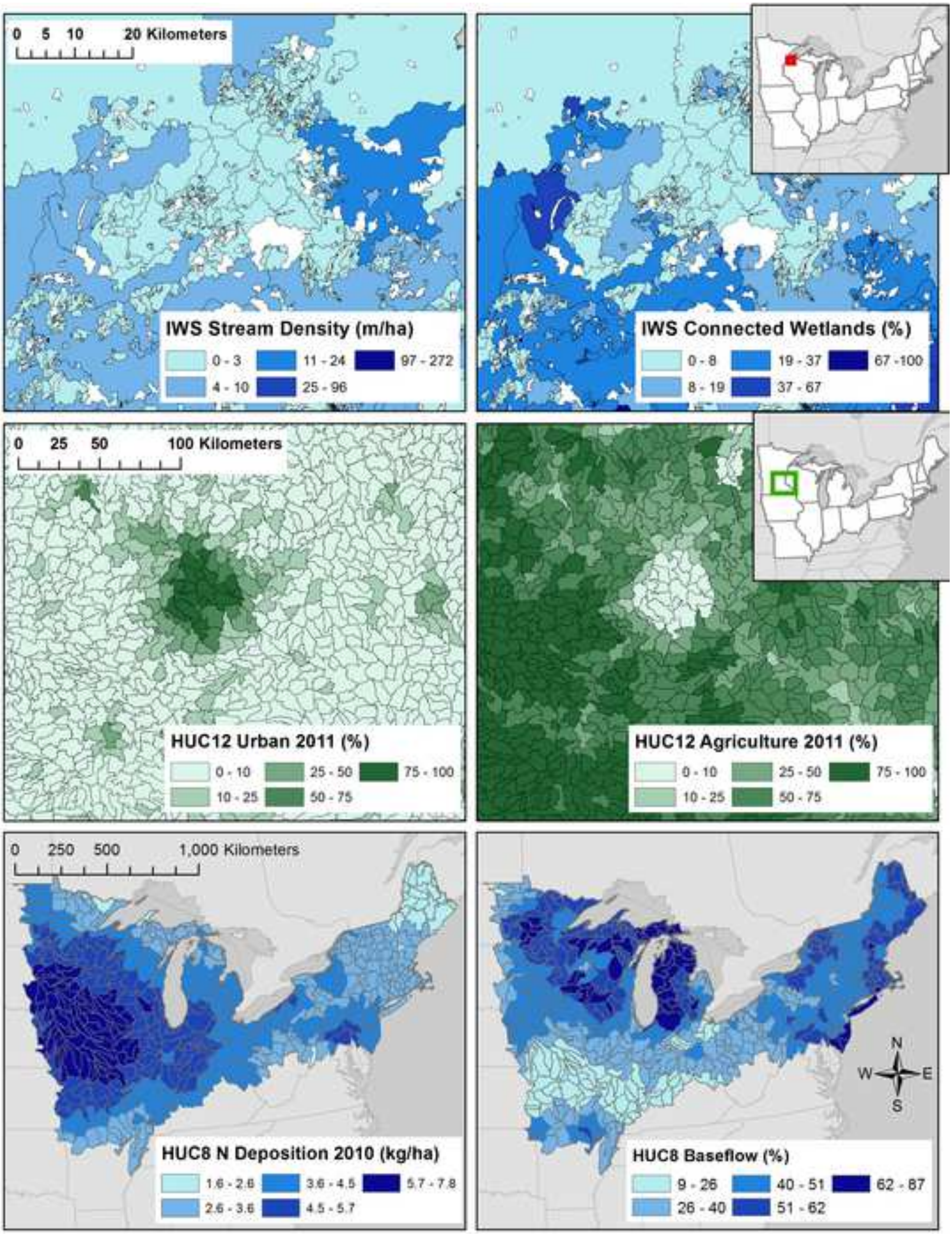

Click here to access/download
Supplementary Material
Soranno_etal_2017_Additional file 1_list of data files.docx

Click here to access/download
Supplementary Material
Soranno_etal_2017_Additional file 2_qaqc-limno_v2.docx

Personal Cover
Click here to download Personal Cover Soranno_etal_gigascience-cover letter.docx

Dr. Goodman, Editor-in-chief
GigaScience

May 16, 2017

Dear Dr. Goodman,

Please find enclosed our manuscript, *LAGOS-NE: A multi-scaled geospatial temporal database of lake ecological context and water quality for thousances of U.S. lakes* by Soranno et al., which we would like to submit for publication as a *Data Note* in GigaScience. We describe and make available a very large integrated, geospatial database for water quality. Given the size and scope of the database, the Data Note is likely longer than many such articles published in this journal. However, we wanted to include sufficient detail to correctly describe the database for future use of scientists. This is the database for which we described previously in a Review article published in GigaScience in 2015 (https://gigascience.biomedcentral.com/articles/10.1186/s13742-015-0067-4). For this Data Note, we are making the data available, and describing the data itself. Given the reception that the previous article received, we think the research community will find our database useful. Also, we appreciated the flexibility that GigaScience provided us in drafting our previous paper that describe the methods in detail, and having the data paper published in the same journal seems to make sense so that future data users can find both papers easily. We have decided to place the data in the Environmental Data Initiative data repository as described in our paper.

We confirm that this manuscript has not been published elsewhere and is not under consideration by another journal. All authors have approved the manuscript, agree with its submission to GigaScience, and have no competing interests.

We recommend the following reviewers:
Dr. Carly Strasser, DataCite Organization, Oakland, CA carlystrasser@gmail.com
Dr. Simon Goring, University of Wisconsin, Madison, goring@wisc.edu
Dr. Matthew Jones, University of California, Santa Barbara, jones@nceas.ucsb.edu

We look forward to hearing from you.

Sincerely,

Dr. Patricia A. Soranno, corresponding author
Professor
Michigan State University

Michigan State University
College of Agriculture and Natural Resources
DEPARTMENT OF FISHERIES AND WILDLIFE
480 Wilson Road, Room 13
Natural Resources Bldg.
East Lansing, MI 48824
517/355-4478
FAX: 517/432-1699

MSU is an affirmative-action, equal-opportunity employer.
