# Supplementary material for: LAGOS-NE: a multi-scaled geospatial and temporal database of lake ecological context and water quality for thousands of US lakes
Source: Gigascience. 2017 Oct 19;6(12):1–22. doi: 10.1093/gigascience/gix101 (PMC5721373; doi:10.1093/gigascience/gix101)
Supplement: Additional File [file soranno_etal_2017_additional_file_1_8sep17_final.docx]

**Additional file 1**

List of data and resources for LAGOS-NE available for download

In this data paper, we make the following research products available:

1. **Data tables** with the data that make up LAGOS-NE
2. An **R package** for accessing the data tables and integrating and querying different variables from the tables. This package greatly facilitates interaction and usability with the many tables that make up LAGOS-NE. The package loads in the data from the data repository and allows users to select certain variables rather than ingesting all of the data at once.
3. **GIS coverages** of freshwater features (lakes, streams, and wetlands) that were used to create many of the metrics in LAGOS-NE
4. **Individual water quality datasets, metadata, and code** that were used to create LAGOS-NE_LIMNO_, including for each dataset: individual EML metadata files; R code to translate the source data model into the LAGOS-NE data model; a log file with notes about that process; and the translated dataset that got loaded into LAGOS-NE.

**1. Data tables**

**LAGOS-NE_LIMNO_ v1.087.1**

<http://dx.doi.org/10.6073/pasta/56cc5f1f753d48edfea170a5401dd6df>

*Lake morphometry*

LAGOSNE_lakeslimno10871.csv

*Water quality data and metadata*

LAGOSNE_epinutr10871.csv

LAGSONE_secchi10871.csv*

*Metadata of the source program*

LAGOSNE_sourceprogram10871.csv

**LAGOS-NE_LOCUS_ v1.01**

<http://dx.doi.org/doi:10.6073/pasta/0c23a789232ab4f92107e26f70a7d8ef>

*Lake ID, location, and zone IDs*

LAGOSNE_LakesLocus101.csv

**LAGOS-NE_GEO_ v1.05**

<http://dx.doi.org/doi:10.6073/pasta/16f4bdaa9607c845c0b261a580730a7a>

*Lake context*

LAGOSNE_lakesgeo101.csv

*Zone characteristics*

LAGOSNE_state_105.csv

LAGOSNE_county_105.csv

LAGOSNE_edu_105.csv

LAGOSNE_hu4_105.csv

LAGOSNE_hu8_105.csv

LAGOSNE_hu12_105.csv

LAGOSNE_iws_105.csv

LAGOSNE_buffer500m_105.csv

LAGOSNE_buffer100m_105.csv

*CHAG theme* (climate, hydrology, atmospheric deposition of N and S, and surficial geology)

LAGOSNE_state_chag105.csv

LAGOSNE_county_chag105.csv

LAGOSNE_edu_chag105.csv

LAGOSNE_hu4_chag105.csv

LAGOSNE_hu8_chag105.csv

LAGOSNE_hu12_chag105.csv

*LULC theme* (land use/cover, canopy cover, terrain metrics, road density, and dam density)

LAGOSNE_state_lulc105.csv

LAGOSNE_county_lulc105.csv

LAGOSNE_edu_lulc105.csv

LAGOSNE_hu4_lulc105.csv

LAGOSNE_hu8_lulc105.csv

LAGOSNE_hu12_lulc105.csv

LAGOSNE_iws_lulc105.csv

LAGOSNE_buffer500m_lulc105.csv

LAGOSNE_buffer100m_lulc105.csv

*CONN theme* (lake, stream, and wetland abundance and connectivity metrics)

LAGOSNE_state_conn105.csv

LAGOSNE_county_conn105.csv

LAGOSNE_edu_conn105.csv

LAGOSNE_hu4_conn105.csv

LAGOSNE_hu8_conn105.csv

LAGOSNE_hu12_conn105.csv

LAGOSNE_iws_conn105.csv

*** Note, although there are Secchi depth data in the *LAGOSNE_epinutr10871.csv* file, it is only for lakes in which there are also nutrient data collected. In the *LAGSONE_secchi10871.csv* file, there are data from lakes that only have Secchi depth data, i.e., there are many lakes for which only Secchi data were collected and those data are only in the *LAGSONE_secchi10871.csv* file.

**2. An R package for accessing the data in this data paper**

<https://github.com/cont-limno/LAGOS>

**3. GIS coverages of the freshwater features (lakes, streams, and wetlands) that are linked to the data tables for GIS processing**

<http://dx.doi.org/doi:10.6073/pasta/8674fd113c0089c0fa174ee4eaf3f376>

LAGOS_NE_GIS_Data_v1.0.gdb (in addition to individual shape files)

**4. Individual water quality datasets and associated metadata and code for LAGOS-NE_LIMNO_**

For each water quality dataset, we provide the following in each data package

- EML metadata files for each of the 87 limnological datasets
- R code to translate the source data model into the LAGOS-NE data model
- A log file with notes about the translation proess
- The source dataset that got loaded into LAGOS-NE

CT_DEEP_CHEM

Landing page:

<https://portal.edirepository.org/nis/mapbrowse?scope=edi&identifier=54&revision=1>

Detailed metadata page:

<https://portal.edirepository.org/nis/metadataviewer?packageid=edi.54.1>

CT_DEEP_CHEM_GRANT_PROGRAM

Landing page:

<https://portal.edirepository.org/nis/mapbrowse?scope=edi&identifier=121&revision=1>

Detailed metadata page:

<https://portal.edirepository.org/nis/metadataviewer?packageid=edi.121.1>

CT_DEEP_SECCHI

Landing page:

<https://portal.edirepository.org/nis/mapbrowse?scope=edi&identifier=66&revision=1>

Detailed metadata page:

<https://portal.edirepository.org/nis/metadataviewer?packageid=edi.66.1>

CT_POST_CHL

Landing page:

<https://portal.edirepository.org/nis/mapbrowse?scope=edi&identifier=12&revision=1>

Detailed metadata page:

<https://portal.edirepository.org/nis/metadataviewer?packageid=edi.12.1>

CT_POST_NUTRIENTS

Landing page:

<https://portal.edirepository.org/nis/mapbrowse?scope=edi&identifier=13&revision=1>

Detailed metadata page:

<https://portal.edirepository.org/nis/metadataviewer?packageid=edi.13.1>

CT_POST_SECCHI

Landing page:

<https://portal.edirepository.org/nis/mapbrowse?scope=edi&identifier=14&revision=1>

Detailed metadata page:

<https://portal.edirepository.org/nis/metadataviewer?packageid=edi.14.1>

EPA_ELS_PHASE_I

Landing page:

<https://portal.edirepository.org/nis/mapbrowse?scope=edi&identifier=71&revision=1>

Detailed metadata page:

<https://portal.edirepository.org/nis/metadataviewer?packageid=edi.71.1>

EPA_ELS_PHASE_II

Landing page:

<https://portal.edirepository.org/nis/mapbrowse?scope=edi&identifier=79&revision=1>

Detailed metadata page:

<https://portal.edirepository.org/nis/metadataviewer?packageid=edi.79.1>

EPA_EMAP_CHEM

Landing page:

<https://portal.edirepository.org/nis/mapbrowse?scope=edi&identifier=46&revision=1>

Detailed metadata page:

<https://portal.edirepository.org/nis/metadataviewer?packageid=edi.46.1>

EPA_NLA_CHEM

Landing page:

<https://portal.edirepository.org/nis/mapbrowse?scope=edi&identifier=48&revision=1>

Detailed metadata page:

<https://portal.edirepository.org/nis/metadataviewer?packageid=edi.48.1>

EPA_TIME_CHEM

Landing page:

<https://portal.edirepository.org/nis/mapbrowse?scope=edi&identifier=65&revision=1>

Detailed metadata page:

<https://portal.edirepository.org/nis/metadataviewer?packageid=edi.65.1>

IA_CHEMISTRY

Landing page:

<https://portal.edirepository.org/nis/mapbrowse?scope=edi&identifier=91&revision=1>

Detailed metadata page:

<https://portal.edirepository.org/nis/metadataviewer?packageid=edi.91.1>

IA_RATHBUN_CHEM

Landing page:

<https://portal.edirepository.org/nis/mapbrowse?scope=edi&identifier=86&revision=2>

Detailed metadata page:

<https://portal.edirepository.org/nis/metadataviewer?packageid=edi.86.2>

IL_ALMP_1999_2000

Landing page:

<https://portal.edirepository.org/nis/mapbrowse?scope=edi&identifier=80&revision=1>

Detailed metadata page:

<https://portal.edirepository.org/nis/metadataviewer?packageid=edi.80.1>

IL_ALMP_2001_2004

Landing page:

<https://portal.edirepository.org/nis/mapbrowse?scope=edi&identifier=81&revision=1>

Detailed metadata page:

<https://portal.edirepository.org/nis/metadataviewer?packageid=edi.81.1>

IL_ALMP_2004_2005

Landing page:

<https://portal.edirepository.org/nis/mapbrowse?scope=edi&identifier=82&revision=1>

Detailed metadata page:

<https://portal.edirepository.org/nis/metadataviewer?packageid=edi.82.1>

IL_ALMP_2006_2008

Landing page:

<https://portal.edirepository.org/nis/mapbrowse?scope=edi&identifier=83&revision=1>

Detailed metadata page:

<https://portal.edirepository.org/nis/metadataviewer?packageid=edi.83.1>

IL_ALMP_2009

Landing page:

<https://portal.edirepository.org/nis/mapbrowse?scope=edi&identifier=84&revision=1>

Detailed metadata page:

<https://portal.edirepository.org/nis/metadataviewer?packageid=edi.84.1>

IL_ALMP_CHMPG_2006

Landing page:

<https://portal.edirepository.org/nis/mapbrowse?scope=edi&identifier=85&revision=1>

Detailed metadata page:

<https://portal.edirepository.org/nis/metadataviewer?packageid=edi.85.1>

IL_ALMP_SPRNGFLD_2004_2006

Landing page:

<https://portal.edirepository.org/nis/mapbrowse?scope=edi&identifier=55&revision=1>

Detailed metadata page:

<https://portal.edirepository.org/nis/metadataviewer?packageid=edi.55.1>

IN_chemistry

Landing page:

<https://portal.edirepository.org/nis/mapbrowse?scope=edi&identifier=70&revision=1>

Detailed metadata page:

<https://portal.edirepository.org/nis/metadataviewer?packageid=edi.70.1>

MA_DEP_2005_2010

Landing page:

<https://portal.edirepository.org/nis/mapbrowse?scope=edi&identifier=56&revision=2>

Detailed metadata page:

<https://portal.edirepository.org/nis/metadataviewer?packageid=edi.56.2>

MA_DEP_CHEM

Landing page:

<https://portal.edirepository.org/nis/mapbrowse?scope=edi&identifier=72&revision=1>

Detailed metadata page:

<https://portal.edirepository.org/nis/metadataviewer?packageid=edi.72.1>

MA_QUABBIN_CHEM

Landing page:

<https://portal.edirepository.org/nis/mapbrowse?scope=edi&identifier=18&revision=1>

Detailed metadata page:

<https://portal.edirepository.org/nis/metadataviewer?packageid=edi.18.1>

MA_QUABBIN_SECCHI

Landing page:

<https://portal.edirepository.org/nis/mapbrowse?scope=edi&identifier=19&revision=1>

Detailed metadata page:

<https://portal.edirepository.org/nis/metadataviewer?packageid=edi.19.1>

MA_WACHUSETT_CHEM

Landing page:

<https://portal.edirepository.org/nis/mapbrowse?scope=edi&identifier=49&revision=1>

Detailed metadata page:

<https://portal.edirepository.org/nis/metadataviewer?packageid=edi.49.1>

ME_ANP_post_2006

Landing page:

<https://portal.edirepository.org/nis/mapbrowse?scope=edi&identifier=20&revision=1>

Detailed metadata page:

<https://portal.edirepository.org/nis/metadataviewer?packageid=edi.20.1>

ME_ANP_pre_2006

Landing page:

<https://portal.edirepository.org/nis/mapbrowse?scope=edi&identifier=21&revision=1>

Detailed metadata page:

<https://portal.edirepository.org/nis/metadataviewer?packageid=edi.21.1>

ME_DEP_CHEM

Landing page:

<https://portal.edirepository.org/nis/mapbrowse?scope=edi&identifier=61&revision=1>

Detailed metadata page:

<https://portal.edirepository.org/nis/metadataviewer?packageid=edi.61.1>

MI_CORPS_CHEM

Landing page:

<https://portal.edirepository.org/nis/mapbrowse?scope=edi&identifier=22&revision=1>

Detailed metadata page:

<https://portal.edirepository.org/nis/metadataviewer?packageid=edi.22.1>

MI_DEQ_COLOR

Landing page:

<https://portal.edirepository.org/nis/mapbrowse?scope=edi&identifier=23&revision=1>

Detailed metadata page:

<https://portal.edirepository.org/nis/metadataviewer?packageid=edi.23.1>

MI_DEQ_HIST_LWQS

Landing page:

<https://portal.edirepository.org/nis/mapbrowse?scope=edi&identifier=93&revision=1>

Detailed metadata page:

<https://portal.edirepository.org/nis/metadataviewer?packageid=edi.93.1>

MI_DEQ_LWQA

Landing page:

<https://portal.edirepository.org/nis/mapbrowse?scope=edi&identifier=94&revision=1>

Detailed metadata page:

<https://portal.edirepository.org/nis/metadataviewer?packageid=edi.94.1>

MI_DOUGLAS_SECCHI

Landing page:

<https://portal.edirepository.org/nis/mapbrowse?scope=edi&identifier=24&revision=1>

Detailed metadata page:

<https://portal.edirepository.org/nis/metadataviewer?packageid=edi.24.1>

MI_Hamilton_KBS_CHEM

Landing page:

<https://portal.edirepository.org/nis/mapbrowse?scope=edi&identifier=25&revision=1>

Detailed metadata page:

<https://portal.edirepository.org/nis/metadataviewer?packageid=edi.25.1>

MI_LEELANAU_CHEM

Landing page:

<https://portal.edirepository.org/nis/mapbrowse?scope=edi&identifier=95&revision=1>

Detailed metadata page:

<https://portal.edirepository.org/nis/metadataviewer?packageid=edi.95.1>

MI_LTBB_CHEM

Landing page:

<https://portal.edirepository.org/nis/mapbrowse?scope=edi&identifier=26&revision=1>

Detailed metadata page:

<https://portal.edirepository.org/nis/metadataviewer?packageid=edi.26.1>

MI_MSU_CHERUVELIL

Landing page:

<https://portal.edirepository.org/nis/mapbrowse?scope=edi&identifier=92&revision=1>

Detailed metadata page:

<https://portal.edirepository.org/nis/metadataviewer?packageid=edi.92.1>

MI_MSU_LNDSCP_CHEM

Landing page:

<https://portal.edirepository.org/nis/mapbrowse?scope=edi&identifier=122&revision=1>

Detailed metadata page:

<https://portal.edirepository.org/nis/metadataviewer?packageid=edi.122.1>

MI_MSU_SONAR_CHEM

Landing page:

<https://portal.edirepository.org/nis/mapbrowse?scope=edi&identifier=27&revision=1>

Detailed metadata page:

<https://portal.edirepository.org/nis/metadataviewer?packageid=edi.27.1>

MI_PAUL

Landing page:

<https://portal.edirepository.org/nis/mapbrowse?scope=edi&identifier=28&revision=1>

Detailed metadata page:

<https://portal.edirepository.org/nis/metadataviewer?packageid=edi.28.1>

MI_TIP_MITT_CHEM

Landing page:

<https://portal.edirepository.org/nis/mapbrowse?scope=edi&identifier=29&revision=1>

Detailed metadata page:

<https://portal.edirepository.org/nis/metadataviewer?packageid=edi.29.1>

MI_TIP_MITT_VOLUNTEER

Landing page:

<https://portal.edirepository.org/nis/mapbrowse?scope=edi&identifier=30&revision=1>

Detailed metadata page:

<https://portal.edirepository.org/nis/metadataviewer?packageid=edi.30.1>

MI_TMDL_PLATTE_CHEM

Landing page:

<https://portal.edirepository.org/nis/mapbrowse?scope=edi&identifier=50&revision=1>

Detailed metadata page:

<https://portal.edirepository.org/nis/metadataviewer?packageid=edi.50.1>

MN_FDL_chemistry

Landing page:

<https://portal.edirepository.org/nis/mapbrowse?scope=edi&identifier=64&revision=1>

Detailed metadata page:

<https://portal.edirepository.org/nis/metadataviewer?packageid=edi.64.1>

MN_GPR_CHEM

Landing page:

<https://portal.edirepository.org/nis/mapbrowse?scope=edi&identifier=53&revision=1>

Detailed metadata page:

<https://portal.edirepository.org/nis/metadataviewer?packageid=edi.53.1>

MN_MPCA_CHEM

Landing page:

<https://portal.edirepository.org/nis/mapbrowse?scope=edi&identifier=47&revision=1>

Detailed metadata page:

<https://portal.edirepository.org/nis/metadataviewer?packageid=edi.47.1>

MN_MPCA_CHEM_1999_2012

Landing page:

<https://portal.edirepository.org/nis/mapbrowse?scope=edi&identifier=63&revision=3>

Detailed metadata page:

<https://portal.edirepository.org/nis/metadataviewer?packageid=edi.63.3>

MN_MPCA_SECCHI

Landing page:

<https://portal.edirepository.org/nis/mapbrowse?scope=edi&identifier=31&revision=1>

Detailed metadata page:

<https://portal.edirepository.org/nis/metadataviewer?packageid=edi.31.1>

MN_SENTINEL

Landing page:

<https://portal.edirepository.org/nis/mapbrowse?scope=edi&identifier=57&revision=2>

Detailed metadata page:

<https://portal.edirepository.org/nis/metadataviewer?packageid=edi.57.2>

MN_SHINGOBEE_SECCHI

Landing page:

<https://portal.edirepository.org/nis/mapbrowse?scope=edi&identifier=32&revision=1>

Detailed metadata page:

<https://portal.edirepository.org/nis/metadataviewer?packageid=edi.32.1>

MN_WILLIAMS_SECCHI

Landing page:

<https://portal.edirepository.org/nis/mapbrowse?scope=edi&identifier=33&revision=1>

Detailed metadata page:

<https://portal.edirepository.org/nis/metadataviewer?packageid=edi.33.1>

MO_LMVP_CHEM

Landing page:

<https://portal.edirepository.org/nis/mapbrowse?scope=edi&identifier=34&revision=1>

Detailed metadata page:

<https://portal.edirepository.org/nis/metadataviewer?packageid=edi.34.1>

MO_UM_SLAP_1978_2013

Landing page:

<https://portal.edirepository.org/nis/mapbrowse?scope=edi&identifier=35&revision=1>

Detailed metadata page:

<https://portal.edirepository.org/nis/metadataviewer?packageid=edi.35.1>

NH_LKTROPH

Landing page:

<https://portal.edirepository.org/nis/mapbrowse?scope=edi&identifier=58&revision=1>

Detailed metadata page:

<https://portal.edirepository.org/nis/metadataviewer?packageid=edi.58.1>

NH_LKTROPH _1995_2014

Landing page:

<https://portal.edirepository.org/nis/mapbrowse?scope=edi&identifier=52&revision=1>

Detailed metadata page:

<https://portal.edirepository.org/nis/metadataviewer?packageid=edi.52.1>

NH_SUNAPEE

Landing page:

<https://portal.edirepository.org/nis/mapbrowse?scope=edi&identifier=67&revision=1>

Detailed metadata page:

<https://portal.edirepository.org/nis/metadataviewer?packageid=edi.67.1>

NH_VLAP

Landing page:

<https://portal.edirepository.org/nis/mapbrowse?scope=edi&identifier=96&revision=1>

Detailed metadata page:

<https://portal.edirepository.org/nis/metadataviewer?packageid=edi.96.1>

NH_VLAP_1995_2014

Landing page:

<https://portal.edirepository.org/nis/mapbrowse?scope=edi&identifier=97&revision=1>

Detailed metadata page:

<https://portal.edirepository.org/nis/metadataviewer?packageid=edi.97.1>

NJ_DEP_CHEM

Landing page:

<https://portal.edirepository.org/nis/mapbrowse?scope=edi&identifier=36&revision=1>

Detailed metadata page:

<https://portal.edirepository.org/nis/metadataviewer?packageid=edi.36.1>

NY_ADIRONDACK_1984

Landing page:

<https://portal.edirepository.org/nis/mapbrowse?scope=edi&identifier=69&revision=2>

Detailed metadata page:

<https://portal.edirepository.org/nis/metadataviewer?packageid=edi.69.2>

NY_ADIRONDACK_1992

Landing page:

<https://portal.edirepository.org/nis/mapbrowse?scope=edi&identifier=37&revision=1>

Detailed metadata page:

<https://portal.edirepository.org/nis/metadataviewer?packageid=edi.37.1>

NY_CDEP_CATSKILL

Landing page:

<https://portal.edirepository.org/nis/mapbrowse?scope=edi&identifier=38&revision=1>

Detailed metadata page:

<https://portal.edirepository.org/nis/metadataviewer?packageid=edi.38.1>

NY_CSLAP

<https://portal.edirepository.org/nis/mapbrowse?scope=edi&identifier=39&revision=1>

Landing page:

<https://portal.edirepository.org/nis/metadataviewer?packageid=edi.39.1>

Detailed metadata page:

NY_LCI_CHEM

Landing page:

<https://portal.edirepository.org/nis/mapbrowse?scope=edi&identifier=87&revision=1>

Detailed metadata page:

<https://portal.edirepository.org/nis/metadataviewer?packageid=edi.87.1>

NY_ONEIDA_CHEM

Landing page:

<https://portal.edirepository.org/nis/mapbrowse?scope=edi&identifier=88&revision=1>

Detailed metadata page:

<https://portal.edirepository.org/nis/metadataviewer?packageid=edi.88.1>

NY_SENECA

Landing page:

<https://portal.edirepository.org/nis/mapbrowse?scope=edi&identifier=68&revision=1>

Detailed metadata page:

<https://portal.edirepository.org/nis/metadataviewer?packageid=edi.68.1>

OH_MIAMI_CHEM

Landing page:

<https://portal.edirepository.org/nis/mapbrowse?scope=edi&identifier=62&revision=1>

Detailed metadata page:

<https://portal.edirepository.org/nis/metadataviewer?packageid=edi.62.1>

OH_ODW_CHEM

Landing page:

<https://portal.edirepository.org/nis/mapbrowse?scope=edi&identifier=40&revision=1>

Detailed metadata page:

<https://portal.edirepository.org/nis/metadataviewer?packageid=edi.40.1>

PA_DEP_CHEM

Landing page:

<https://portal.edirepository.org/nis/mapbrowse?scope=edi&identifier=73&revision=1>

Detailed metadata page:

<https://portal.edirepository.org/nis/metadataviewer?packageid=edi.73.1>

PA_WALLENPAUPACK_CHEM

Landing page:

<https://portal.edirepository.org/nis/mapbrowse?scope=edi&identifier=78&revision=1>

Detailed metadata page:

<https://portal.edirepository.org/nis/metadataviewer?packageid=edi.78.1>

RI_URIWW_CHEM

Landing page:

<https://portal.edirepository.org/nis/mapbrowse?scope=edi&identifier=41&revision=1>

Detailed metadata page:

<https://portal.edirepository.org/nis/metadataviewer?packageid=edi.41.1>

UMWLTM

Landing page:

<https://portal.edirepository.org/nis/mapbrowse?scope=edi&identifier=89&revision=1>

Detailed metadata page:

<https://portal.edirepository.org/nis/metadataviewer?packageid=edi.89.1>

VT_DWQ_NUTRIENT

Landing page:

<https://portal.edirepository.org/nis/mapbrowse?scope=edi&identifier=59&revision=1>

Detailed metadata page:

<https://portal.edirepository.org/nis/metadataviewer?packageid=edi.59.1>

WI_BIOCOM

Landing page:

<https://portal.edirepository.org/nis/mapbrowse?packageid=knb-lter-ntl.41.5>

<https://portal.edirepository.org/nis/mapbrowse?packageid=knb-lter-ntl.42.5>

<https://portal.edirepository.org/nis/mapbrowse?packageid=knb-lter-ntl.209.4>

<https://portal.edirepository.org/nis/mapbrowse?packageid=knb-lter-ntl.44.5>

Detailed metadata page:

<https://portal.edirepository.org/nis/metadataviewer?packageid=knb-lter-ntl.41.5>

<https://portal.edirepository.org/nis/metadataviewer?packageid=knb-lter-ntl.42.5>

<https://portal.edirepository.org/nis/metadataviewer?packageid=knb-lter-ntl.209.4>

<https://portal.edirepository.org/nis/metadataviewer?packageid=knb-lter-ntl.44.5>

WI_CROSS_LAKE

Landing page:

<https://portal.edirepository.org/nis/mapbrowse?packageid=knb-lter-ntl.220.6>

Detailed metadata page:

<https://portal.edirepository.org/nis/metadataviewer?packageid=knb-lter-ntl.220.6>

WI_DNR_NUTRIENT

Landing page:

<https://portal.edirepository.org/nis/mapbrowse?scope=edi&identifier=74&revision=1>

Detailed metadata page:

<https://portal.edirepository.org/nis/metadataviewer?packageid=edi.74.1>

WI_FCPC_CHEM

Landing page:

<https://portal.edirepository.org/nis/mapbrowse?scope=edi&identifier=75&revision=1>

Detailed metadata page:

<https://portal.edirepository.org/nis/metadataviewer?packageid=edi.75.1>

WI_HANSON

Landing page:

<https://portal.edirepository.org/nis/mapbrowse?scope=knb-lter-ntl&identifier=277>

Detailed metadata page:

<https://portal.edirepository.org/nis/metadataviewer?packageid=knb-lter-ntl.277.2>

WI_L_POSITION_CHEM

Landing page:

<https://portal.edirepository.org/nis/mapbrowse?scope=edi&identifier=76&revision=1>

Detailed metadata page:

<https://portal.edirepository.org/nis/metadataviewer?packageid=edi.76.1>

WI_L_POSITION_CHL

Landing page:

<https://portal.edirepository.org/nis/mapbrowse?scope=edi&identifier=77&revision=1>

Detailed metadata page:

<https://portal.edirepository.org/nis/metadataviewer?packageid=edi.77.1>

WI_LDF_CHEM

Landing page:

<https://portal.edirepository.org/nis/mapbrowse?scope=edi&identifier=60&revision=1>

Detailed metadata page:

<https://portal.edirepository.org/nis/metadataviewer?packageid=edi.60.1>

WI_LKLS

Landing page:

<https://portal.edirepository.org/nis/mapbrowse?scope=edi&identifier=42&revision=1>

Detailed metadata page:

<https://portal.edirepository.org/nis/metadataviewer?packageid=edi.42.1>

WI_LTER_NUTRIENTS

Landing page:

<https://portal.edirepository.org/nis/mapbrowse?packageid=knb-lter-ntl.1.12>

Detailed metadata page:

<https://portal.edirepository.org/nis/metadataviewer?packageid=knb-lter-ntl.1.12>

WI_LTER_SECCHI

Landing page:

<https://portal.edirepository.org/nis/mapbrowse?packageid=knb-lter-ntl.31.8>

Detailed metadata page:

<https://portal.edirepository.org/nis/metadataviewer?packageid=knb-lter-ntl.31.8>

WI_LTER_TROUT_CHL

Landing page:

<https://portal.edirepository.org/nis/mapbrowse?scope=knb-lter-ntl&identifier=35>

Detailed metadata page:

<https://portal.edirepository.org/nis/metadataviewer?packageid=knb-lter-ntl.35.20>

WI_WERL

Landing page:

<https://portal.edirepository.org/nis/mapbrowse?scope=edi&identifier=104&revision=1>

Detailed metadata page:

<https://portal.edirepository.org/nis/metadataviewer?packageid=edi.104.1>
