# Supplementary material for: LAGOS-NE: a multi-scaled geospatial and temporal database of lake ecological context and water quality for thousands of US lakes
Source: Gigascience. 2017 Oct 19;6(12):1–22. doi: 10.1093/gigascience/gix101 (PMC5721373; doi:10.1093/gigascience/gix101)
Supplement: Additional File [file soranno_etal_2017_additional_file_2_qaqc-limno_v2.docx]

**Additional file 2**

The quality assurance/quality control (QAQC) procedure and results for

LAGOS-NE_LIMNO_ v1.087.1

*Prepared by Noah Lottig and Patricia Soranno*

*April 2017*

**Overview of qaqc procedures for LAGOS-NE_LIMNO_ v1.087.1**

Our goal for this effort was to identify *egregiously* high values and values that might be too low, both defined below. Note that our quality control procedures were not designed to identify statistical outliers, which individual users are expected to perform themselves because such analyses depend on the subsequent statistical analysis of each user. There were three major phases in the quality assurance/quality control (QAQC) procedure for LAGOS-NE_LIMNO_. Phase I was designed to identify and flag values that were lower than analytically possible (i.e., below detection limits) when there was sufficient metadata; however, note that although these data are flagged, they were still provided in this data paper because it is not appropriate to remove data that are below detection. Therefore, each data value in LAGOS-NELIMNO has an associated *censorcode* associated with it. Phases II and III were designed to identify the egregious values that we defined as those that: 1) did not make ecological sense, 2) were far beyond what has been detected in previous studies, 3) were not technically feasible (e.g., SRP > TP), or 4) were a result of a data or file corruption or error in the data loading stage. For these egregious values, we explored the issues that might be underlying the values and removed them from the LAGOS-NE_LIMNO_ data export provided in this data paper because we did had insufficient confidence that they were scientifically valid data. We were very conservative in these assessments to avoid not including data values that were high, yet still valid. These LAGOS QAQC-flags are retained in the master LAGOS-NE database, but are not exported in this data paper because the data values themselves are not exported in this data paper.

For all versions of LAGOS-NE_LIMNO_ exports, the QAQC was conducted on the entire cumulative dataset to leverage as large of a sample size as possible to detect problem values. In other words, because most of the QAQC analyses and tests described here make use of all information from many lakes or multiple variables, incorporating new data may result in a better assessment of the data quality than when there is less data. Thus, for each new version of LAGOS-NE_LIMNO_, new decisions are made about egregious values. In this data paper and this document, we describe the procedures for assessing all major versions of LAGOS-NE_LIMNO_, but we present the results only for this version of LAGOS-NE_LIMNO_ (v1.087.1).

Because there are few accepted practices for conducting quality control analyses on such a large, integrated database, we created our own procedures for Phase II and III by creating tests to identify egregious values that leverage a large, integrated database with multiple measures of water quality and well-established expected relationships among variables. The database that we used to identify egregious values was based on data in the full LAGOS-NE_LIMNO_ database for samples taken from all lake depths provided by the source datasets (note, our data exports in this data paper are only for epilimnetic or surface samples). While the quality control procedures that we implemented here were designed to help identify egregious errors in a combined dataset across many programs, there are likely additional extreme values in the database due to the size and heterogeneity of the data. Users should check for additional issues in the data values such as outlier detection that are specific to intended analyses.

**Phase I – Creating of LAGOS-NE_LIMNO_ censor codes based on detection limit information**

We created a LAGOS-NE_LIMNO_ specific flag for water quality data values that get exported for all variables in LAGOS-NE_LIMNO_. This flag, is called **_censorcode* (where * signifies which water quality variable the code is assigned to) because it identifies whether a value is censored or not based on the relation of the data value to a known analytical detection limit. The need to consider the role of censored observations in data analyses of integrated databases may be complicated by the fact that detection limits for the same characteristic chemical constituent can differ depending on the sampling and measurement protocols. These protocols may differ among data providers and may have changed over time. For this data flag, we are trying to identify data values that are below a detection limit of a particular analytical approach. It has been well-established that data that are below detection should not simply be deleted or dealt with in other simple ways (such as assigning the value to ½ of the detection limit [1]). Therefore, when possible, we have documented the detection limit and created a flag that identifies the relationship of a value and its detection limit if it is known.

Because of the wide range of ways that detection limits are reported by different data users, we created nine unique codes describing the different relationships between: the data value itself, the detection limit if available, and the presence of a ‘censor-code’ from the data provider (Table S1). This data flag provides future users of LAGOS-NE_LIMNO_ with the information that we were able to glean from metadata for these data sources.

**Phase II – QAQC at the program level to detect systematic errors in data-loading**

In Phase II, we analyze limnological data at the level of the individual program and variable. For each program, we examine individual variables, and, when possible, combinations of two variables between which we expect a relationship based on known limnological relationships. In this phase, we seek to identify problems or errors at the program level or at various steps in the data import. When problems are identified, they are fixed at the central LAGOS-NE_LIMNO_ database level and the data are re-exported before proceeding to the next QAQC phase. In general, this phase does not result in assigning flags to data; rather, it is used as a diagnostic step to identify major problems in data import/export.

The steps are as follows:

1. Import data:
   1. Read in all LAGOS-NE_LIMNO_ data (for all lakes and lake depths) into R
   2. Query the database to identify data values that had a LAGOS-NE_LIMNO_ *censorcode* with one of the 9 options described above. We then excluded those data from the remaining QAQC procedures because they have already been QAQC’d by the source program and we have no further information to improve on the assessment of the quality of the data. However, these censored data values WERE included in the final LAGOS-NE_LIMNO_ export that is provided in this data paper, along with the appropriate censor code.
2. Variable diagnostics: For each Program/Variable combination, generate general diagnostic plots of the data that include histograms, boxplots, the number of observations, and the variance of those observations. Because ecological data are often not normally distributed and characterized by fat tails, we use robust boxplots that account for the skewness of the data [2]. Histograms and boxplots provide similar types of information in that they are both representations of the distribution of the data, but provide slightly different representations of those distributions.
   1. *Distribution plots (shape)*: Examine the histogram shapes for each program and for each variable. For each variable, we compare across programs by visually assessing whether the distribution diverges from the general patterns observed in other programs. Specifically, we target bimodal distributions, extremely skewed data, or other patterns that diverge drastically from the other programs. In general, these divergent patterns can indicate that variables were recorded in more than one measurement unit (e.g., feet and meters; or mg/L and µg/L).
   2. *Distribution plots (mode and range)*: Compare mode and range of distribution plots (both histograms and robust boxplots) to determine whether program level unit conversion issues exist. For example, units of a variable were thought to be mg/L and converted to µg/L when in fact the units were originally in µg/L. Typically, this results in distributions being shifted by approximately three orders of magnitude for variables measured as concentrations.
   3. *Variance*: Very low variance in the data is suggestive of constant values (e.g., all values the same, such as 10.4) and may indicate a data import problem. This occurred twice in the QAQC process for two separate variables in different programs. In both cases, the reported values were below the detection limit because at a program level, no measurement was ever made that was above the detection limit. This observation was one of the driving factors for Phase I-1a.
3. Limnological relationships: Many ecological variables tend to change in predictable ways, such as the positive relationship between chlorophyll a (Chla) and total phosphorus (TP). At the program level, we generate all combinations of bivariate plots (e.g., TP vs Chla plots) for the variables included in LAGOS-NE_LIMNO_. For each bivariate plot, we use quantile regression to estimate the slope of the relationship between the two variables. Slopes from the bivariate plots are examined to assess whether a program does not follow known ecological relationships or varies substantially relative to the other programs. Relationship errors typically occurred as a result of errors in the source data or in the data importing stage.
4. Strategy to deal with data problems: If problems are identified in steps 2-3, then the data administrator examines the data beginning with the original source raw data, then the data-import step, and finally the export step to identify the possible problem(s). The data administrator fixes the problem and re-exports a new version of LAGOS-NE_LIMNO_ for the remaining QAQC steps.
5. We repeated steps 1-3 to ensure the problems identified are fixed.

**Phase III – QAQC of individual and combinations of variables**

Phase III of the QAQC procedure identifies anomalies in the data at the variable level, in contrast to Phase II, which identified program level issues. Here, we take a variety of approaches, including:

- combining information from the LAGOS-NE_LIMNO_ data, professional knowledge, and published literature to determine realistic ranges in values for parameters
- comparing different forms of parameters such as total and dissolved phosphorus to determine if values are inconsistent with known relationships (e.g., dissolved cannot be greater than total fractions)
- identifying observations using multiple different parameters that are inconsistent with limnological relationships (e.g., Chla/TP relationship)
- identifying observations within a lake that are inconsistent with the historical record in the lake

A significant amount of effort was placed in generating robust approaches that identify and flag egregious values in an automated fashion so as to remove observer bias and ensure that the process was repeatable regardless of who was processing the data. Table S2 describes the five specific tests that we developed to conduct this part of the QAQC analysis of the LAGOS-NE_LIMNO_ database. Note, it is still assumed that censored data are still not included in the database that undergoes the next QAQC steps to identify egregious values.

**A. TEST: Zero values**

1. This test is based on the assumption that because it is unlikely that any analytical method can quantify a true zero value for the water quality variables in the database, these values with no associated censor code were considered potentially suspect. We identified values that have a value of zero.
2. Generate a table of the ZERO-flagged values for the database administrator to add to the QAQC-flag in the LAGOS-NE_LIMNO_ database (but that is not exported in the final database).
3. Exclude the ZERO-flagged values from the QAQC dataset for the remaining steps. The ZERO-flagged values are no longer part of the QAQC process for remaining Phase II steps because a data value that has any of these QAQC flags will not be exported for future analysis, but we do retain them in our LAGOS-NE database for future reference.

**C. TEST: Maximum Allowable Values (MAV) and Ceiling Values**

1. Using best professional judgement, we determined the Ceiling Values that were defined as values of variables that are deemed not possible in lakes. These values were conservatively set quite high.
2. Identify any values that are greater than or equal to the Ceiling Value (Table S2), and send the list of values to the database administrator to remove from LAGOS-NE permanently. Note, these are the only values that are ever removed from the database permanently.
3. To determine MAVs, we took a multi-pronged approach. First, we searched the literature to find articles that report global or broad-scale datasets for water quality variables to identify upper ranges for as many variables as possible. Then, for variables with few studies, we consulted multiple aquatic ecologists to identify maximum values for each variable using best professional judgement. Then, finally, we took a data-driven approach for those variables that had at least 10,000 data points, which we describe below. 10,000 was determined as a cut-off for sufficient data based on a randomization analysis for TP and CHL data, for which we have very large sample sizes.
   1. For each variable with at least 10,000 values, calculate the IQR range using the robust boxplot analysis (see below)
   2. For each variable, we examined a range values from 4 – 15 times the IQRs to try to balance the need to identify sufficient values that were likely truly egregious with the desire to retain high, but real extreme values in the dataset.
   3. We selected a value of 10 times the IQR as our definition of the MAV for the variables for which we did not have a MAV estimated another way. The value of 10 times the IQR was selected because it only excluded a few data points and resulted in MAVs that were significantly greater than values observed in programs in which we expected truly high values and that also had good QAQC procedures.
   4. Compile the list of MAVs to use for the analysis (Table S3)
4. Identify values that exceed the MAV and flag them as 'MAV' (Table S2).
5. Generate a table of the MAV-flagged values for the database administrator to add to the QAQC-flag in the LAGOS-NE_LIMNO_ database (but that is not exported in the final database).

6. Exclude the MAV-flagged values from the QAQC dataset for the remaining steps. The MAV-flagged values are no longer part of the QAQC process for remaining steps because a data value that has any of these QAQC flags will not be exported for future analysis, but we do retain them in our LAGOS-NE database for future reference.

**C. TEST: Analysis of bounded relationships for select variables**

1. The bounded relationship (RATIO) QAQC step assumes that species of nutrients cannot be greater than the TOTAL measures of those nutrients (TP and TN). Calculate nitrogen and phosphorus ratios such as total dissolved phosphorus (TDP)/TP, total Kjeldahl nitrogen (TKN)/total nitrogen (TN) when known quantities are bounded by another known parameter (e.g., TDP can never be greater than TP). See Table S4 for a list of all calculated ratios.
2. Flag the values that have ratios >1.1 as 'RATIO'.
3. Generate a table of RATIO-flagged values for the database administrator to add to the QAQC-flag in the LAGOS-NE_LIMNO_ database (but that is not exported in the final database).
4. Exclude the RATIO-flagged values from the QAQC dataset for the remaining steps. The RATIO-flagged values are no longer part of the QAQC process for remaining Phase II steps because a data value that has any of these QAQC flags will not be exported for future analysis, but we do retain them in our LAGOS-NE database for future reference.

**D. TEST: Distance-based outlier test**

1. The distance-based outlier (BIPLOT) QAQC step assumes that every variable is related in some systematic way to the other variables for all of LAGOS-NELIMNO data. Generate bi-plot matrixes of all combinations of variables in LAGOS-NE_LIMNO_ using data from all programs at any depth.
2. Calculate the Euclidian distances between all bi-plot points when more than 20 points exist and extract the Euclidian distance to the 5th nearest point (k value). K values between 3 and 20 were compared and we determined that k = 5 appears to provide an optimal balance of identifying points that appeared to be outliers in bi-plot space while retaining points that are not egregious.
3. Calculate boxplots of the Euclidian distances and identify outlier biplot points that have Euclidian distances greater than 15 times the IQR of the boxplot. These points were then given a flag value of ‘BIPLOT’ for both variables (i.e., the X and the Y variable from the original biplot). We determined that 15 times the IQR was a conservative estimate in retaining data. It excludes only those values whose Euclidian distances are extreme relative to all other values in a given bi-plot space. See Figure S1 for an example plot.
4. Generate table of BIPLOT-flagged values for the database administrator to add to the QAQC-flag in the LAGOS-NE_LIMNO_ database (but that is not exported in the final database).
5. Exclude the BIPLOT-flagged values from the QAQC dataset for the remaining steps. The BIPLOT-flagged values are no longer part of the QAQC process for remaining Phase II steps because a data value that has any of these QAQC flags will not be exported for future analysis, but we do retain them in our LAGOS-NE database for future reference.

**E. TEST: Analysis of variables by depth through time**

1. The time-test (TIME) QAQC step assumes that lake-level variables are temporally related to one another from year to year. This test identifies lake-specific egregious values, and only can be used for lakes that have been sampled through time (on at least 5 separate dates).
2. Split the dataset into two separate datasets. A near surface (EPI) dataset is composed of 1) all samples designated as EPI, and 2) samples from depth < 10 m when the stratum is not designated but depth is, and 3) any sample with unknown sample depth or stratum (based on the assumption that if a sampling program collects samples without depth specified that it is likely taken from the surface). The second dataset is a hypolimnion (HYPO) dataset and contains all data that are identified as HYPO samples, and, when the stratum is not identified but the depth is, those samples that have a depth of ≥ 10m. This step is conducted only for this analysis and is driven by the fact that hypolimnion TP concentrations may, for example, be very different from epilimnion concentrations during stratified periods. Consequently, within a lake, the distributions of data might be very different and thus should be considered separately when identifying outliers using discrete time-series of data without other co-varying information.
3. Identify lakes in LAGOS-NE_LIMNO_ that have had an individual variable measured on at least five different dates over the entire sampling record. Thus, each lake must have at least five observations for a given variable (but the number of observations could be greater if multiple depths within each stratum were measured on the same date).
   1. For each identified lake, analyze each variable for egregiously large values using the EPI and HYPO datasets separately. We exclude data from lakes with time series data that have median absolute deviations equal to zero due to analytical difficulties (e.g., TP values of 1.2, 1.2, 1.2, 1.2, 0.5, 1.2, 1.2, 1.5) would result in both 0.5 and 1.5 being identified as egregious values).
   2. Lake-specific egregious values were identified when the generalized extreme studentized deviate test indicated an ‘outlier’ at p = 0.001 and the outlier is greater than eight times the IQR value using robust boxplots. See Figure S1 for an example plot.
4. Generate table of TIME-flagged values for the database administrator to add to the QAQC-flag in the LAGOS-NE_LIMNO_ database (but that is not exported in the final database).

**F. Summarize the outcome of the QAQC procedures for Phase II-III**

The database administrator summarizes, by variable and by QAQC procedure, the numbers of data values identified by these procedures to evaluate the potential problems with the database as a whole. Note that the data that have been identified through these procedures are not exported to data users because these values are deemed egregious by our procedures and we consider the data associated with those flags to be untrustworthy.

**Results of the QAQC analysis for LAGOS-NE_LIMNO_ v1.087.1**

**Phase I**

Censored observations were a small percentage of the entire database; for programs that had information on detection limits, then only 0.5%, 2.5% and 3% of the values for TN, TP, and Chl, respectively, were censored compared to those that were not (Table S5). However, across all observations for these 3 variables, 76%, 33% and 45% of the observations contained information on detection limits for TN, TP, and Chl respectively.

**Phase II and III**

In total, 3.7% of the data values across all variables were identified to be egregious based on one of these five tests; and, not surprisingly, variables with the highest number of egregious values were dissolved nutrient fractions, which can have low concentrations in lake surface waters, and are likely to have large measurement errors (Table S5). All variables had egregious values that were detected from at least 3 or more of the tests.

**References**

1. Helsel D. 2010. Much ado about next to mothing: Incorporating nondetects in science. Annals of Occupational Hygiene 54: 257-262.

2. Vanderviere E, Huber M. An adjusted boxplot for skewed distributions. COMPSTAT’2004 Symposium. 2004. https://wis.kuleuven.be/stat/robust/papers/2004/boxplotCOMPSTAT04.pdf. Accessed 27 May 2015.

**Table S1. LAGOS censor codes**

| **Censorcode** | **Description** |
| --- | --- |
| GT | has no upper detectionlimit, datavalue is greater than or equal to an upper detection limit, may or may not have qualifiers |
| NC1 | has detectionlimit and datavalue is above detectionlimit and has no qualifier or comments |
| NC2 | has detectionlimit and datavalue is above detectionlimit, and has qualifier or comments |
| NC3 | has no detectionlimit and has qualifier or comments |
| NC4 | has no detectionlimit and has no qualifiers or comments |
| LE1 | has detectionlimit, datavalue is less than or equal to detectionlimit, has qualifier or comments |
| LE2 | has detectionlimit, datavalue is less than or equal to detectionlimit, has no qualifier or comments |
| LE3 | has no detectionlimit, < than comes from sourceprogram, has qualifier or comments |
| LE4 | has no detectionlimit, < than comes from sourceprogram, has no qualifier or comments |

A description of the censor codes that we developed for LAGOS-NELIMNO to determine exactly the type of information available from the source program and the relationship among a data value, the detection limit, or a censor code provided by the source program.

**Table S2. LAGOS-NE_LIMNO_ QAQC flag codes and their definitions**

| **QAQC flag codes** | **Description** | **Limnological data type relevant to QAQC flag** | **LAGOS-NE_LIMNO_ variables relevant to QAQC flag** |
| --- | --- | --- | --- |
| ZERO | Value reported as zero with no associated flags or censor codes from data provider (excluding Secchi) | All sampling events | All LAGOS-NE_LIMNO_ variables except Secchi |
| MAV | Maximum allowable value exceeded. Values are excluded from all LAGOS-NE_LIMNO_ exports | All sample events | All LAGOS-NE_LIMNO_ variables |
| RATIO | Measured ratio of N, P, or C not feasible (e.g., TDP > 1.1 x TP). Values are excluded from all LAGOS-NE_LIMNO_ exports | All sample events | Only nitrogen, phosphorus, and carbon variables |
| TIME | Value was identified as outlier based on generalized extreme studentized deviate tests (p = 0.001) and > 8 x IQR using robust boxplots | Only individual lakes with at least 5 years of repeated measures | All LAGOS-NE_LIMNO_ variables |
| BIPLOT | Value was identified as an outlier using distance-based outlier algorithm | All sample events | All LAGOS-NE_LIMNO_ variables |

For each QAQC flag code, we describe the test, the data that was used for the test, and the variables that the test could be applied to.

**Table S3. Maximum allowable values (MAVs) and Ceiling Values that are used in the QAQC process**

| **Variable** | **Units** | **Maximum Allowable Values*** | **Ceiling Values**** |
| --- | --- | --- | --- |
| Chla | µg/L | 800 | 1,600 |
| Colora | PCU | 600 | 1,200 |
| Colort | PCU | 600 | 1,200 |
| DKN | µg/L | 21,000 | 40,000 |
| DOC | mg/L | 100 | 165 |
| NH4 | µg/L | 21,000 | 40,000 |
| NO2 | µg/L | 21,000 | 40,000 |
| NO2NO3 | µg/L | 21,000 | 40,000 |
| Secchi | m | 32 | 32 |
| SRP | µg/L | 2,000 | 10,000 |
| TDN | µg/L | 21,000 | 40,000 |
| TDP | µg/L | 2,000 | 10,000 |
| TKN | µg/L | 21,000 | 40,000 |
| TN | µg/L | 21,000 | 40,000 |
| TOC | mg/L | 332 | 332 |
| TON | µg/L | 21,000 | 40,000 |
| TP | µg/L | 2,000 | 10,000 |

* The MAVs were determined as a result of the analysis described in this document and so represent the maximum allowed in the LAGOS-NE_LIMNO_ v1.087.1 data export. MAVs were estimated from a combination of sources including: the literature to find articles that report global or broad-scale datasets for water quality variables to identify upper ranges for as many variables as possible; best professional judgement of multiple aquatic ecologists; and, a data-driven approach for those variables that had at least 10,000 data points.

** Values greater than or equal to the Ceiling values were deleted from LAGOS-NE_LIMNO_.

**Table S4. Bounded relationship ratios examined in the LAGOS-NE_LIMNO_ QAQC process**

| **Major Nutrient** | | |
| --- | --- | --- |
| **Phosphorus** | **Nitrogen** | **Carbon** |
| TDP:TP | TON:TN | DOC:TOC |
| SRP:TP | TKN:TN |  |
| SRP:TDP | TDN:TN |  |
|  | NO2NO3:TN |  |
|  | NO2:TN |  |
|  | NH4:TN |  |
|  | DKN:TN |  |
|  | NO2:NO2NO3 |  |
|  | DKN:TKN |  |
|  | NH4:TKN |  |
|  | NH4:DKN |  |
|  | TON:TKN |  |
|  | (NO2NO3+NH4):TKN |  |
|  | (NO2NO3+NH4):TDN |  |
|  | (TN-TKN):NO2NO3 |  |

**Table S5.** **Overview of censored and non-censored data in the LAGOS-NE database for the three variables with the most data.**

|  |  |  | **Water quality variable** | | | |
| --- | --- | --- | --- | --- | --- | --- |
| **Measure** | | **Units** | **Chl a** | **TN** | **TP** |  |
| *(a) Programs* | | *Total n* | *58* | *34* | *68* |  |
|  | With DL | *%* | 25.9 | 29.4 | 32.3 |  |
|  | With and without DL | *%* | 8.6 | 5.9 | 11.8 |  |
|  | With tagged (<) observations | *%* | 8.6 | 2.9 | 16.2 |  |
|  | No DL information | *%* | 56.9 | 61.8 | 39.7 |  |
| *(b) Observations from programs* | | *Total n* | *209732* | *41671* | *158974* |  |
|  | With DL | *%* | 45.1 | 75.7 | 32.8 |  |
|  | With and without DL | *%* | 16.0 | 7.7 | 8.7 |  |
|  | With tagged (<) observations | *%* | 19.5 | 2.3 | 41.6 |  |
|  | No DL information | *%* | 19.4 | 14.4 | 16.9 |  |
| *(c) Censored Observations* | | *Total n* | *5088* | *192* | *3265* |  |
|  | Observations from all programs | *%* | 2.4 | 0.46 | 2.1 |  |
|  | Observations from programs with DL | *%* | 3.0 | 0.54 | 2.5 |  |
| *(d) DL (µg/L) from censored and non-censored* | | *Total n* | *103297* | *32401* | *62912* |  |
|  |  | median | 1 | 50 | 2 |  |
|  |  | mean | 0.8 | 68.9 | 2.2 |  |
|  |  | min | 0.001 | 3.2 | 0.3 |  |
|  |  | max | 10 | 500 | 110 |  |
| *(e) DL (µg/L) from censored* | | *Total n* | *4292* | *183* | *2280* |  |
|  |  | median | 1 | 84 | 10 |  |
|  |  | mean | 1.0 | 147.5 | 8.5 |  |
|  |  | min | 0.1 | 20 | 0.3 |  |
|  |  | max | 10 | 280 | 50 |  |

(a) The number of individual programs supplying datasets and the percent with DL (detection limits), with a combination of observations with and without DL, with tags indicating censored observations, and with no DL information. (b) The total number of observations and percentages from each group described in (a). (c) The number of censored observations and the percent censored among all observations and from programs with DL specified. (d) The number and summary statistics for DL where provided for both censored and non-censored observations. (e) The number and summary statistics for DL from censored observations.

**Table S6. Results of the Phase III QAQC conducted on LAGOS-NE_LIMNO_ v1.087.1.**

| **Variable** | **Zero** | **MAV** | **Ratio** | **Biplot** | **Time** | **Total number values** | **Percent values identified as egregious** |
| --- | --- | --- | --- | --- | --- | --- | --- |
| Chlorophyll *a* | 233 | 16 | *na* | 29 | 202 | 263,388 | 0.2 |
| Secchi depth | *na* |  | *na* | 9 | 119 | 913,542 | 0.0 |
| Apparent color | 32 | 3 | *na* |  | 15 | 45,079 | 0.1 |
| True color | 40 | 52 | *na* | 1 | 21 | 48,600 | 0.2 |
| Total organic carbon |  |  | 751 | 12 | 72 | 12,504 | 6.7 |
| Dissolved organic carbon | 6 | 73 | 751 | 16 | 77 | 45,168 | 2.0 |
| Ammonium | 626 | 2,077 | 1,084 | 26 | 54 | 92,203 | 4.2 |
| Nitrite | 437 | 1 | 329 |  | 25 | 10,441 | 7.6 |
| Nitrate + nitrite | 1,866 | 5 | 4,095 | 38 | 95 | 119,351 | 5.1 |
| Dissolved Kjeldahl nitrogen |  | 6 | 49 |  | 5 | 5,844 | 1.0 |
| Total dissolved nitrogen | 2 | 15 | 884 | 3 | 7 | 11,168 | 8.2 |
| Total Kjeldahl nitrogen |  | 25 | 3,835 | 34 | 67 | 96,980 | 4.1 |
| Total organic nitrogen | 201 |  | 3 | 1 | 2 | 5,179 | 4.0 |
| Total nitrogen | 12 | 14 | 4,323 | 24 | 28 | 66,069 | 6.7 |
| Soluble reactive phosphorus | 51 | 211 | 390 | 4 | 40 | 27,767 | 2.5 |
| Total dissolved phosphorus | 836 | 97 | 666 | 11 | 35 | 17,733 | 9.3 |
| Total phosphorus | 120 | 613 | 828 | 35 | 472 | 282,577 | 0.7 |


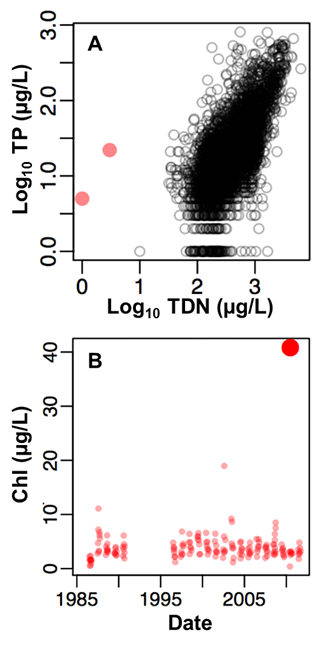
**Figure S1**. **Example output from quality control tests for LAGOS-NE_LIMNO_.** Large, red-filled symbols in both plots represent observations that one of the tests identified as suspect based on the population of observations in all lakes with data for the BIPLOT test (A) or for a single lake through time for the TIME test (B).
