# Supplementary material for: LAGOS-NE: a multi-scaled geospatial and temporal database of lake ecological context and water quality for thousands of US lakes
Source: Gigascience. 2017 Oct 19;6(12):1–22. doi: 10.1093/gigascience/gix101 (PMC5721373; doi:10.1093/gigascience/gix101)
Supplement: Response-to-Reviewer-Comments_Original-Submission.pdf [file gix101_response-to-reviewer-comments_original-submission.pdf]

Author's Response To Reviewer Comments

Response to Reviewer reports
Soranno et al. Gigascience; July 14, 2017
GIGA-D-17-00112 -- LAGOS-NE: A multi-scaled geospatial and temporal database of lake ecological context and water quality for thousands of U.S. lakes
Reviewer #1:
The compilation of LAGOS-NE is truly impressive work and I am really excited that the data is made publicly available now. I am sure this is a great resource for future work by you and by others.
Overall, I liked the manuscript and was impressed by the care that went into creating and describing the database.
Besides a few smaller comments (see remarks below and the comments in the attached pdf), I have only one more fundamental critique:
I believe that usability and value of the database would be considerably improved, if the concepts were linked wherever that is possible to concepts grounded in ontologies. I know that not all concepts are modeled in ontologies yet, but at least for those that are, it would be good to provide that link instead of creating your own definitions. For those that are not in ontologies yet, it would be great, if you worked towards their addition. In the long run, this would ease the integration of LAGOS-NE with other upcoming databases and would contribute to a common understanding of the domain.
[I don't see this linkage as a prerequisite to publication of the paper, but would really like to see it in the future].
RESPONSE: We completely agree with the reviewer, however, currently, our community is not quite there yet. However, in our next LAGOS data project, we will strive to connect to existing ontologies and we are currently involved in an effort to construct and add a lake characteristic-related ontology to the existing ENVO (https://bioportal.bioontology.org/ontologies/ENVO). We are collaborating with the curator of ENVO, computer scientists in Germany and semantics specialists in the US to accomplish this task. This is currently in the PhD thesis stage, once we are confident that we can connect the LAGOS concepts to the ENVO ontology we will do so in future LAGOS databases.

You write in the introduction that you provide the water quality files, however, that URL is missing. I thus was not able to have a look at these files.
RESPONSE: We have updated all URLs so they are all now active.

Here are the smallish remarks:
* I found a number of typos in the manuscript and marked them directly in the pdf. Please refer to the attachment.
RESPONSE: We have fixed all identified typos that the reviewer identified in the manuscript that was attached, which we found to be very helpful. However, we have not removed brackets as those are required by the journal. In addition, the land use percentages do not add up to 100% because we only include the 4 dominant types. We have added this to the table legend.

* I had a look at the datasets and found some minor issues there:

* The metadata about the LAGOS-LakeID says that this is a float and a ratio. Is that really correct?
RESPONSE: We have changed all ID's to type character.

* In LAGOS_LIMNO the limno definition (YYYY-MM-DD) and Format: mm/DD/YYYY of sample date are inconsistent.
RESPONSE: We have we corrected the date time format of the EML to be MM/DD/YYYY. This is the actual date time format used in the data table.

* In LAGOS_GEO both min and max values of county_pct_in_nwi are 100%. Is that correct?
RESPONSE: yes, it is ok to have all 100%. For other spatial extents, these values are not always 100%.

Reviewer #2:
Review pertaining to general limnological information, manuscript text, and data sets:
Gertrud Nürnberg, Ph.D., Freshwater Research (www.fwr.ca)
Review pertaining to data management and R-related files: Stefanie LaZerte, Ph.D. (steffilazerte.ca)
Not reviewed: GIS related information

This manuscript describes and publishes the data files that were used in a previously published paper about methods of data base creation [17]. The authors are to be commended on this effort of making their large data set not only accessible, but also describing data quality/control/variability and providing data management tools for easy access and analysis. Such efforts should be supported and definitely warrant publication in a platform like GigaScience.

This manuscript repeats some of the general information published previously [17] in the introduction, general purpose, etc., which could be deleted. However, the text as assembled here may help the data file user to find much information without major searches in the previous paper; I therefore leave it up to the authors and the editors to decide whether shortening of the text is possible and necessary.
RESPONSE: We agree and have tried to make this paper a standalone paper with as little overlap as possible. Therefore we have decided to leave the text as is.

While some sections are repeated from the previous paper, an interesting and useful new section is provided at the end in Section 9. "Challenges and recommendations for creating large, integrated, and heterogeneous databases".

But I find costs provided in "The economic value of water quality data in an integrated database" (791-805) out of proportion. The cost estimate of a single lake sample of $2000-6000, based on stream sampling, seems extremely high (line 799). Consider the inexpensive Secchi data and other data collected by volunteers. Commercial water TP analysis is typically less than Can$45, and physical profile data (temperature, oxygen) do not require special expertise and time after an initial investments into equipment (<$5000, depending on lake depth).
On the other hand, the section on "Strategies for broad-scale data-integration efforts" (lines 807-

858) is well thought out and should help other, similar endeavours.
RESPONSE: we agree that there could be some cost savings in lakes, but then again, lake sampling also requires boats, trailers, etc that many stream sampling efforts do not. We did not include costs for secchi samples, and only include records for which a lab analysis is required. Nevertheless, as recommended, we lowered the range compared to stream samples of $1000-$4000 rather than $2000-$6000. This rough estimate is only intended to put the dataset and costs in context.

One strength of the chosen approach is the modular build. This make it possible to add potentially useful information, such as:
* Information pertaining to internal P loading, including discrete depth samples of phosphorus, iron and manganese.
* Information pertaining to cyanobacteria proliferation and blooms: Maximum chlorophyll concentration, phytoplankton species and biomass, cyanotoxins

Additional documents and files are extensive. They seem to explain and describe methods of data selection and other approaches used in detail. I believe that a potential user can find all the information needed to determine the data validity.

Detailed comments in the order of the text by line numbers follow:
105: Also indicate the number of nutrient data, especially of total phosphorus (TP).
RESPONSE: Done. We have added TP

107: Were there no data used from the published peer-reviewed scientific literature?
RESPONSE: No, we have found it sometimes too difficult to acquire the metadata for such studies, as well as the data themselves because historically, it has not been the practice to put data into data repositories. It was more efficient to get data directly from sources, and state agency datasets are larger, and contain more data than published studies typically.

140-1: A fitting reference would also be:
--Bachmann, R.W., Hoyer, M.V., and Canfield Jr, D.E. 2013. The extent that natural lakes in the United States of America have been changed by cultural eutrophication. Limnol. Oceanogr 58(3): 945-950.
RESPONSE: We have chosen not to cite this article due to the numerous responses to the article that were published questioning their conclusions.

157-160: It would be great to test this assumption of lacking metadata for the lake data (and not just citing river data and reference [16]).
RESPONSE: Yes, we agree, however, it is beyond the scope of our data paper to include this estimate. Further, we do not have any reason to expect it to differ greatly between lake and stream samples. Nevertheless, we are now working more closely with the authors of this article who are employees at the USGS for the next phase of our research to build LAGOS for the entire US by integrating more with the Water Quality Portal.

195: It would be helpful to be more specific: what time periods are usually provided (before 2012)?

RESPONSE: we agree. We have added: mostly from the late 1980’s to up until about 2012.

255: Replace "were" with "was" (grammar)
RESPONSE: done

327-331: Phosphorus retention in lakes is not usually complete (100%) so the notion of "trapping" TP in any large upstream lakes is an oversimplification. Nonetheless, retention of large and deep lakes without internal loading is usually 70-90%, so that the assumption of R=100% is more valid than R=0%.
--Brett, M.T., and Benjamin, M.M. 2008. A review and reassessment of lake phosphorus retention and the nutrient loading concept. Freshw. Biol. 53: 194-211.
--Nürnberg, G.K. 1984. The prediction of internal phosphorus load in lakes with anoxic hypolimnia. Limnol. Oceanogr. 29: 111-124.
RESPONSE: We agree, but have chosen not to add citations as this is not a major focus of this manuscript and the paper that we cite also cites these papers within it.

405: It is confusing that in Table 2: "… lakes are counted for each state in which they occur (i.e., lakes that straddle two states are counted in both states)", while in other files such lakes are counted only once.
RESPONSE: We agree, however, there is little that we can do that would not require a complete GIS analysis to reclassify lakes by state and make decisions about which border lake belongs where. Unfortunately, lakes do not follow state borders, and different table summaries make different assumptions. We felt the important part of this table was to show the relative numbers of lakes by lake type rather than the state data, so slight discrepancies due to border issues was acceptable.

476: "All data in LAGOS-NELIMNO v1.087.1 are from samples that we identified as being collected from either the lake surface or the epilimnion (the well-mixed surface layer of a thermally-stratified lake during the period of stratification)." As mentioned above, it would be useful to expand the dataset to include data that can be used to determine whether there is any sediment P release. Such data include hypolimnetic and discrete deep water samples during the stratification period in stratified lakes.
RESPONSE: We certainly agree, and in fact some of those data reside in the master LAGOS-NE database, we just have not sufficiently processed them to make them available, nor do we have the associated temperature and dissolved oxygen profiles that would make those values even more useful. However, for the next version of LAGOS-US, we will include both oxygen and temperature profiles and possibly, lake nutrients at depth.

625: "We have published 10 articles using portions of this database". Perhaps these and the 13 articles in review (if available when this ms is published), could be listed and cited in a separate table. But perhaps the subsequent paragraph already refers to these references?
RESPONSE: Correct, the later paragraph describes them and cites the published studies. We would rather not provide citations to the in prep manuscripts in a table since those will likely change in the coming months and soon be out of date. However, we have updated any manuscripts that have now been published so that there are fewer ‘in preparation’ manuscripts that we discuss in this section. Further, we have chosen not to include a table of papers because

this is not the main focus of this manuscript, and this section is intended to only show that many publications have used this database.

808: This sentence is not complete ("which" is awkward)
RESPONSE: We have fixed by adding ‘and to identify the types of datasets….

843: I think you mean "disseminate" rather than "dissemination"
RESPONSE: fixed.

Data management and R-related files: reviewed by Stefanie LaZerte

This R package is a nice way of providing access to this large dataset. The package was generally easy to install and easy to use. I wasn't able to use lagos_get() to download, as it got through most but failed on one file. It was nice that the function detected previously downloaded files and resumed. But it would be even nicer if it had the option to skip over files that couldn't be reached.
RESPONSE: Now that all files area available on EDI and we have updated to package to point to them this should not be an issue. We agree that additional flexibility would be a nice feature. We have filed an issue on the Github repository and hope to implement this for users in the future.

I was able to use the files provided in the dropbox folder, by compiling them with the 'lagos_compile()' function, although I needed to fix a couple of typos to make them work:
- '.txt' in LOCUS file needed to be renamed to '.csv'
- 'LakesLocus' should be lowercase
RESPONSE: Again, now that all files are available on EDI and we have updated to package to point to them this should not be an issue. We apologize for the earlier challenges in accessing the data.

Although not crucial, I would suggest having the compile function create individual rds files in a single directory, and then giving users the option of loading select datasets as the whole set is quite a large table.
RESPONSE: We agree that implementing additional flexibility would be a great option for users. We have filed an issue on the Github respository and we hope to implement this in the future.

The data itself was well explained and organized, but there is such a wealth of information it may become confusing. Perhaps consider making the output of ?dataset (e.g. ?county) specific to that particular dataset, so users don't have to scroll through the descriptions of all columns for all tables if they're only interested in the one.
RESPONSE: We agree that there is a very large volume of information. We hope to eventually improve the organization of the metadata to maximize ease of use, which is an ongoing effort.

The ability to select by categories is very cool, and it would be nice to have a category for sample information (i.e. sampling event, lakeid, etc.)

RESPONSE: We agree that this is a fantastic idea and we have added this to our ‘to do list’ for updating the R package in the coming months, which we view as an ongoing process. Nevertheless, the package allows full access to the database now and improves accessibility of the data to other users. We will be working towards making it increasingly user-friendly with such ideas as this one.

Also, although not related to the quality of the dataset, consider including vignettes or more in-depth tutorials, perhaps for how to merge different data sets together or how to extract and transform particular columns (see coding example below). As the data is in wide format as opposed to long (e.g., years are in different columns, as opposed to having a single year column), the data will have to be transformed before most if not all types of analysis. These transformations are not always trivial. By providing some guidance and examples, the accessibility of the data by users less familiar with R can be improved. In particular, if downloading the data separately is expected to be a common place occurrence, there should be instructions for the use of the 'lago_compile()' function.
RESPONSE: We definitely agree and have added a minimal vignette showing basic interaction with LAGOS

Overall I think this package is a convenient way of accessing both the datasets and the metadata. It is well documented and will be very useful to scientists wishing to use the data.

Minor Comments
- For imports, best to give a minimum version number, eg: dplyr (>= 0.7.0)
RESPONSE: Done

- Documentation for categories should read "waterquality" not "water.quality", also what tables does this category refer to?
RESPONSE: Fixed; It refers to the epi.nutr table. The
lagos_select() documentation has been updated to make this more clear

Coding example
```
library(tidyverse)
library(stringr)
library(LAGOS)

dt <- lagos_load(version = "1.087.1")

c <- dt$county.chag %>%
as_tibble() %>%
select(county_zoneid, matches("dep")) %>%
gather(Variable, Value, -county_zoneid) %>%
mutate(Variable = str_replace(Variable, "county_dep_", ""),
Type = str_extract(Variable, "^[^_]+"),
Year = str_extract(Variable, "[0-9]{4}"),
Stat = str_extract(Variable, "[^_]+$")) %>%
select(-Variable)
```

```
x <- c %>%
filter(county_zoneid == "County_107",
Stat != "std")

ggplot(data = x,
aes(x = Year, y = Value, group = Stat, colour = Stat)) +
geom_line() +
facet_wrap(~ Type, ncol = 1, scales = "free_y")
```

----------------------End of review------------------------

Reviewer #3:
This paper provides a valuable documentation of a geospatial database for lakes of the upper midwest and northeast United States. The value of the database is well illustrated visually in non-uniform distributions of quality (Figure 5) and hydrological variables (Figure 6). The main points - some of which could be addressed in a revision of this paper - include:

(1) [comment only] I have a few misgivings about such a large author list. There is a good justification of the authorship and no doubt, with a few self-citations, this paper will become well cited. But it still does not sit entirely comfortably with me, especially when I can still readily pick out simple typographical errors.
RESPONSE: While we agree for more typical research papers, we do not agree for data papers, in which the author list should be as long as the number of individuals who provided data. We are fixing the typographical errors.

(2) I was disappointed that the dataset extended until 2012. This is hardly a contemporary dataset and it raises a question for me about whether the database is sufficiently nimble to allow rapid incorporation of recent data and time series analysis.
RESPONSE: This is a major issue that we are now addressing in a new grant that will create LAGOS for the entire US and try to integrate with the WQX data repository for updates of newer datasets. Also, our work has shown that for many research questions, the spatial data (i.e., many lakes across broad regions) is more important than good temporal resolution.

(3) I was a little concerned about the large number of 'in prep' articles being cited in section 8. Are these all necessary. Could some be substituted or supplemented with recent published articles. Are other articles recent such as:
- Read JS, Winslow LA, Hansen GJA, Van Den Hoek J, Hanson PC, Bruce LC, Markfort CD 2014. Simulating 2368 temperate lakes reveals weak coherence in stratification phenology. Ecological Modelling 291, 142-150.
- Read EK, L Carr, L De Cicco, HA Dugan, PC Hanson, JA Hart, J Kreft, JS Read, LA Winslow. 2017. Water quality data for national-scale aquatic research: The Water Quality Portal. Water Resources Research. doi:10.1002/2016WR019993.
RESPONSE: These above articles do not use LAGOS data. This section of the manuscript, as requested by the journal, is intended to show the potential value of the dataset by showing the

types of research that has been conducted to date. Because it took a long time to complete the database, many manuscripts are still in prep. Although, now, some have been accepted, which we have updated, and in fact, a large number have been published relative to the numbers in preparation, so we have kept them in the manuscript to convey the types of research questions we are addressing with the database.

Minor points (relating mostly to minor typographical issues):
l118: Lake (case). FIXED
l141: in the same way. FIXED
l164: We created a database named LAGOS-NE... FIXED.
l184: composed should be comprised (do a global search) FIXED.
l201: future. UNCLEAR
l235: km^2 (superscript). FIXED.
l279: have FIXED.
l328: remove nutrient (or nutrients). NOT CHANGED AS THIS ALTERS THE MEANING TOO MUCH.
ll332-334: this sentence needs re-worded. DID NOT CHANGE AS WE DID NOT FIGURE OUT A DIFFERENT WAY TO SAY IT.
l383: km^2 (superscript) FIXED.
l435: they to it UNCLEAR
l450: did had? FIXED
l495: 1980s (it is plural not possessive); do global search FIXED.
l541 use [Greek] mu for micro FIXED
l580: The FIXED
l591: proportions would sum to 1 (as opposed to percentages).
RESPONSE: the land use percentages do not add up to 100% because we only include the 4 dominant types. We have added this to the table legend.
l668: are to is FIXED

l843: disseminate FIXED

l802: that cost rather than the cost FIXED

l808: re-word (related to 'which') FIXED
