# Supplementary material for: LAGOS-NE: a multi-scaled geospatial and temporal database of lake ecological context and water quality for thousands of US lakes
Source: Gigascience. 2017 Oct 19;6(12):1–22. doi: 10.1093/gigascience/gix101 (PMC5721373; doi:10.1093/gigascience/gix101)
Supplement: Response-to-Reviewer-Comments_Revision-1.pdf [file gix101_response-to-reviewer-comments_revision-1.pdf]

Author's Response To Reviewer Comments

All suggested changes have been made. Please note that some author affiliations have changed, which I updated in the word file, however, i did not see where to change/add author affiliations to the author-boxes in the online form.

I have also made some minor changes to text and figure/table legends based on an internal USGS review that i was required to do because I have numerous USGS-affiliated co-authors. If you would like to see a track-changes version of the word doc that shows those changes, I can provide that. However, they were mostly minor editorial changes.
