# Supplementary material for: LAGOS-NE: a multi-scaled geospatial and temporal database of lake ecological context and water quality for thousands of US lakes
Source: Gigascience. 2017 Oct 19;6(12):1–22. doi: 10.1093/gigascience/gix101 (PMC5721373; doi:10.1093/gigascience/gix101)
Supplement: Reviewer-1-Report-(Original-Submission).pdf [file gix101_reviewer-1-report-(original-submission).pdf]

Reviewer Report

Title: LAGOS-NE: A multi-scaled geospatial and temporal database of lake ecological context and water quality for thousands of U.S. lakes

Version: Original Submission **Date:** 7/2/2017

Reviewer name: Birgitta Koenig-Ries

Reviewer Comments to Author:

The compilation of LAGOS-NE is truely impressive work and I am really excited that the data is maded publicly available now. I am sure this is a great resource for future work by you and by others.Overall, I liked the manuscript and was impressed by the care that went into creating and describing the database.Besides a few smaller comments (see remarks below and the comments in the attached pdf), I have only one more fundamental critique:I believe that usabiity and value of the database would be considerably improved, if the concepts were linked wherever that is possible to concepts grounded in ontologies. I know that not all concepts are modeled in ontologies yet, but at least for those that are, it would be good to provide that link instead of creating your own definitions. For those that are not in ontologies yet, it would be great, if you worked towards their addition. In the long run, this would ease the integration of LAGOS-NE with other upcoming databases and would contribute to a common understanding of the domain.[I don't see this linkage as a prerequisite to publication of the paper, but would really like to see it in the future].You write in the introduction that you provide the water quality files, however, that URL is missing. I thus was not able to have a look at these files.Here are the smallish remarks:* I found a number of typos in the manuscript and marked them directly in the pdf. Please refer to the attachment.* I had a look at the datasets and found some minor issues there:* The metadata about the LAGOS-LakeID says that this is a float and a ratio. Is that really correct?* In LAGOS_LIMNO the limno definition (YYYY-MM-DD) and Format: mm/DD/YYYY of sample date are inconsistent.* In LAGOS_GEO both min and max values of county_pct_in_nwi are 100%. Is that correct?

Level of Interest

Please indicate how interesting you found the manuscript: An exceptional article

Quality of Written English

Please indicate the quality of language in the manuscript: Needs some language corrections before being published

Declaration of Competing Interests

Please complete a declaration of competing interests, considering the following questions:

- Have you in the past five years received reimbursements, fees, funding, or salary from an organisation that may in any way gain or lose financially from the publication of this manuscript, either now or in the future?
- Do you hold any stocks or shares in an organisation that may in any way gain or lose financially from the publication of this manuscript, either now or in the future?
- Do you hold or are you currently applying for any patents relating to the content of the manuscript?
- Have you received reimbursements, fees, funding, or salary from an organization that holds or has applied for patents relating to the content of the manuscript?
- Do you have any other financial competing interests?
- Do you have any non-financial competing interests in relation to this paper?

If you can answer no to all of the above, write 'I declare that I have no competing interests' below. If your reply is yes to any, please give details below.

I declare that I have no competing interests.

I agree to the open peer review policy of the journal. I understand that my name will be included on my report to the authors and, if the manuscript is accepted for publication, my named report including any attachments I upload will be posted on the website along with the authors' responses. I agree for my report to be made available under an Open Access Creative Commons CC-BY license (http://creativecommons.org/licenses/by/4.0/). I understand that any comments which I do not wish to be included in my named report can be included as confidential comments to the editors, which will not be published.

I agree to the open peer review policy of the journal
