# Supplementary material for: LAGOS-NE: a multi-scaled geospatial and temporal database of lake ecological context and water quality for thousands of US lakes
Source: Gigascience. 2017 Oct 19;6(12):1–22. doi: 10.1093/gigascience/gix101 (PMC5721373; doi:10.1093/gigascience/gix101)
Supplement: Reviewer-2-Report-(Original-Submission).pdf [file gix101_reviewer-2-report-(original-submission).pdf]

Reviewer Report

Title: LAGOS-NE: A multi-scaled geospatial and temporal database of lake ecological context and water quality for thousands of U.S. lakes

Version: Original Submission **Date:** 7/6/2017

Reviewer name: Gertrud Nurnberg

Reviewer Comments to Author:

Reviewers:Review pertaining to general limnological information, manuscript text, and data sets: Gertrud Nürnberg, Ph.D., Freshwater Research (www.fwr.ca)Review pertaining to data management and R-related files: Stefanie LaZerte, Ph.D. (steffilazerte.ca)Not reviewed: GIS related informationThis manuscript describes and publishes the data files that were used in a previously published paper about methods of data base creation [17]. The authors are to be commended on this effort of making their large data set not only accessible, but also describing data quality/control/variability and providing data management tools for easy access and analysis. Such efforts should be supported and definitely warrant publication in a platform like GigaScience.This manuscript repeats some of the general information published previously [17] in the introduction, general purpose, etc., which could be deleted. However, the text as assembled here may help the data file user to find much information without major searches in the previous paper; I therefore leave it up to the authors and the editors to decide whether shortening of the text is possible and necessary.While some sections are repeated from the previous paper, an interesting and useful new section is provided at the end in Section 9. "Challenges and recommendations for creating large, integrated, and heterogeneous databases".But I find costs provided in "The economic value of water quality data in an integrated database" (791-805) out of proportion. The cost estimate of a single lake sample of $2000-6000, based on stream sampling, seems extremely high (line 799). Consider the inexpensive Secchi data and other data collected by volunteers. Commercial water TP analysis is typically less than Can$45, and physical profile data (temperature, oxygen) do not require special expertise and time after an initial investments into equipment (<$5000, depending on lake depth).On the other hand, the section on "Strategies for broad-scale data-integration efforts" (lines 807-858) is well thought out and should help other, similar endeavours.One strength of the chosen approach is the modular build. This make it possible to add potentially useful information, such as:* Information pertaining to internal P loading, including discrete depth samples of phosphorus, iron and manganese.* Information pertaining to cyanobacteria proliferation and blooms: Maximum chlorophyll concentration, phytoplankton species and biomass, cyanotoxinsAdditional documents and files are extensive. They seem to explain and describe methods of data selection and other approaches used in detail. I believe that a potential user can find all the information needed to determine the data validity.Detailed comments in the order of the text by line numbers follow:105: Also indicate the number of nutrient data, especially of total phosphorus (TP).107: Were there no data used from the published peer-reviewed scientific literature?140-1: A fitting reference would also be:Bachmann, R.W., Hoyer, M.V., and Canfield Jr, D.E. 2013. The extent that natural lakes in the United States of America have been changed by cultural eutrophication. Limnol. Oceanogr 58(3): 945-950.157-160: It would be

great to test this assumption of lacking metadata for the lake data (and not just citing river data and reference [16]).195: It would be helpful to be more specific: what time periods are usually provided (before 2012)?255: Replace "were" with "was" (grammar)327-331: Phosphorus retention in lakes is not usually complete (100%) so the notion of "trapping" TP in any large upstream lakes is an oversimplification. Nonetheless, retention of large and deep lakes without internal loading is usually 70-90%, so that the assumption of R=100% is more valid than R=0%.Brett, M.T., and Benjamin, M.M. 2008. A review and reassessment of lake phosphorus retention and the nutrient loading concept. Freshw. Biol. 53: 194-211.Nürnberg, G.K. 1984. The prediction of internal phosphorus load in lakes with anoxic hypolimnia. Limnol. Oceanogr. 29: 111-124.405: It is confusing that in Table 2: "… lakes are counted for each state in which they occur (i.e., lakes that straddle two states are counted in both states)", while in other files such lakes are counted only once.476: "All data in LAGOS-NELIMNO v1.087.1 are from samples that we identified as being collected from either the lake surface or the epilimnion (the well-mixed surface layer of a thermally-stratified lake during the period of stratification)." As mentioned above, it would be useful to expand the dataset to include data that can be used to determine whether there is any sediment P release. Such data include hypolimnetic and discrete deep water samples during the stratification period in stratified lakes.625: "We have published 10 articles using portions of this database". Perhaps these and the 13 articles in review (if available when this ms is published), could be listed and cited in a separate table. But perhaps the subsequent paragraph already refers to these references?808: This sentence is not complete ("which" is awkward)843: I think you mean "disseminate" rather than "dissemination"----------------------------------Data management and R-related files: reviewed by Stefanie LaZerteThis R package is a nice way of providing access to this large dataset. The package was generally easy to install and easy to use. I wasn't able to use lagos_get() to download, as it got through most but failed on one file. It was nice that the function detected previously downloaded files and resumed. But it would be even nicer if it had the option to skip over files that couldn't be reached.I was able to use the files provided in the dropbox folder, by compiling them with the 'lagos_compile()' function, although I needed to fix a couple of typos to make them work:- '.txt' in LOCUS file needed to be renamed to '.csv'- 'LakesLocus' should be lowercaseAlthough not crucial, I would suggest having the compile function create individual rds files in a single directory, and then giving users the option of loading select datasets as the whole set is quite a large table.The data itself was well explained and organized, but there is such a wealth of information it may become confusing. Perhaps consider making the output of ?dataset (e.g. ?county) specific to that particular dataset, so users don't have to scroll through the descriptions of all columns for all tables if they're only interested in the one.The ability to select by categories is very cool, and it would be nice to have a category for sample information (i.e. sampling event, lakeid, etc.)Also, although not related to the quality of the dataset, consider including vignettes or more in-depth tutorials, perhaps for how to merge different data sets together or how to extract and transform particular columns (see coding example below). As the data is in wide format as opposed to long (e.g., years are in different columns, as opposed to having a single year column), the data will have to be transformed before most if not all types of analysis. These transformations are not always trivial. By providing some guidance and examples, the accessibility of the data by users less familiar with R can be improved. In particular, if downloading the data separately is expected to be a common place occurrence, there should be instructions for the use of the 'lago_compile()' function.Overall I think this package is a convenient way of accessing both the datasets and the

metadata. It is well documented and will be very useful to scientists wishing to use the data.Minor Comments- For imports, best to give a minimum version number, eg: dplyr (>= 0.7.0)- Documentation for categories should read "waterquality" not "water.quality", also what tables does this category refer to?Coding examplelibrary(tidyverse)library(stringr)library(LAGOS)dt <- lagos_load(version = "1.087.1")c <- dt$county.chag %>%as_tibble() %>%select(county_zoneid, matches("dep")) %>%gather(Variable, Value, -county_zoneid) %>%mutate(Variable = str_replace(Variable, "county_dep_", ""),Type = str_extract(Variable, "^[^_]+"),Year = str_extract(Variable, "[0-9]{4}"),Stat = str_extract(Variable, "[^_]+$")) %>%select(-Variable)x <- c %>%filter(county_zoneid == "County_107",Stat != "std")ggplot(data = x,aes(x = Year, y = Value, group = Stat, colour = Stat)) +geom_line() +facet_wrap(~ Type, ncol = 1, scales = "free_y")----------------------End of review------------------------

Level of Interest

Please indicate how interesting you found the manuscript: An exceptional article

Quality of Written English

Please indicate the quality of language in the manuscript: Acceptable

Declaration of Competing Interests

Please complete a declaration of competing interests, considering the following questions:

- Have you in the past five years received reimbursements, fees, funding, or salary from an organisation that may in any way gain or lose financially from the publication of this manuscript, either now or in the future?
- Do you hold any stocks or shares in an organisation that may in any way gain or lose financially from the publication of this manuscript, either now or in the future?
- Do you hold or are you currently applying for any patents relating to the content of the manuscript?
- Have you received reimbursements, fees, funding, or salary from an organization that holds or has applied for patents relating to the content of the manuscript?
- Do you have any other financial competing interests?
- Do you have any non-financial competing interests in relation to this paper?

If you can answer no to all of the above, write 'I declare that I have no competing interests' below. If your reply is yes to any, please give details below.

I declare that I have no competing interests'

I agree to the open peer review policy of the journal. I understand that my name will be included on my report to the authors and, if the manuscript is accepted for publication, my named report including any

attachments I upload will be posted on the website along with the authors' responses. I agree for my report to be made available under an Open Access Creative Commons CC-BY license (http://creativecommons.org/licenses/by/4.0/). I understand that any comments which I do not wish to be included in my named report can be included as confidential comments to the editors, which will not be published.

I agree to the open peer review policy of the journal
