# Supplementary material for: LAGOS-NE: a multi-scaled geospatial and temporal database of lake ecological context and water quality for thousands of US lakes
Source: Gigascience. 2017 Oct 19;6(12):1–22. doi: 10.1093/gigascience/gix101 (PMC5721373; doi:10.1093/gigascience/gix101)
Supplement: Reviewer-3-Report-(Original-Submission).pdf [file gix101_reviewer-3-report-(original-submission).pdf]

Reviewer Report

Title: LAGOS-NE: A multi-scaled geospatial and temporal database of lake ecological context and water quality for thousands of U.S. lakes

Version: Original Submission **Date:** 7/10/2017

Reviewer name: David Hamilton

Reviewer Comments to Author:

This paper provides a valuable documentation of a geospatial database for lakes of the upper midwest and northeast United States. The value of the database is well illustrated visually in non-uniform distributions of quality (Figure 5) and hydrological variables (Figure 6). The main points - some of which could be addressed in a revision of this paper - include:(1) [comment only] I have a few misgivings about such a large author list. There is a good justification of the authorship and no doubt, with a few self-citations, this paper will become well cited. But it still does not sit entirely comfortably with me, especially when I can still readily pick out simple typographical errors.(2) I was disappointed that the dataset extended until 2012. This is hardly a contemporary dataset and it raises a question for me about whether the database is sufficiently nimble to allow rapid incorporation of recent data and time series analysis.(3) I was a little concerned about the large number of 'in prep' articles being cited in section 8. Are these all necessary. Could some be substituted or supplemented with recent published articles. Are other articles recent such as:- Read JS, Winslow LA, Hansen GJA, Van Den Hoek J, Hanson PC, Bruce LC, Markfort CD 2014. Simulating 2368 temperate lakes reveals weak coherence in stratification phenology. Ecological Modelling 291, 142-150.- Read EK, L Carr, L De Cicco, HA Dugan, PC Hanson, JA Hart, J Kreft, JS Read, LA Winslow. 2017. Water quality data for national-scale aquatic research: The Water Quality Portal. Water Resources Research. doi:10.1002/2016WR019993.Minor points (relating mostly to minor typographical issues):l118: Lake (case)l141: in the same wayl164: We created a database named LAGOS-NE...l184: composed should be comprised (do a global search)l201: futurel235: km^2 (superscript)l279: havel328: remove nutrient (or nutrients)ll332-334: this sentence needs re-wordedl383: km^2 (superscript)l435: they to itl450: did had?l495: 1980s (it is plural not possessive); do global searchl541 use [Greek] mu for microl580: Thel591: proportions would sum to 1 (as opposed to percentages)l668: are to isl843: disseminatel802: that cost rather than the costl808: re-word (related to 'which')

Level of Interest

Please indicate how interesting you found the manuscript: An article of importance in its field

Quality of Written English

Please indicate the quality of language in the manuscript: Needs some language corrections before being published

Declaration of Competing Interests

Please complete a declaration of competing interests, considering the following questions:

- Have you in the past five years received reimbursements, fees, funding, or salary from an organisation that may in any way gain or lose financially from the publication of this manuscript, either now or in the future?
- Do you hold any stocks or shares in an organisation that may in any way gain or lose financially from the publication of this manuscript, either now or in the future?
- Do you hold or are you currently applying for any patents relating to the content of the manuscript?
- Have you received reimbursements, fees, funding, or salary from an organization that holds or has applied for patents relating to the content of the manuscript?
- Do you have any other financial competing interests?
- Do you have any non-financial competing interests in relation to this paper?

If you can answer no to all of the above, write 'I declare that I have no competing interests' below. If your reply is yes to any, please give details below.

I declare that I have no competing interests.

I agree to the open peer review policy of the journal. I understand that my name will be included on my report to the authors and, if the manuscript is accepted for publication, my named report including any attachments I upload will be posted on the website along with the authors' responses. I agree for my report to be made available under an Open Access Creative Commons CC-BY license (http://creativecommons.org/licenses/by/4.0/). I understand that any comments which I do not wish to be included in my named report can be included as confidential comments to the editors, which will not be published.

I agree to the open peer review policy of the journal
